# Improved characterization of medically relevant fungi in the human respiratory tract using next-generation sequencing

**DOI:** 10.1186/s13059-014-0487-y

**Published:** 2014-10-25

**Authors:** Kyle Bittinger, Emily S Charlson, Elizabeth Loy, David J Shirley, Andrew R Haas, Alice Laughlin, Yanjie Yi, Gary D Wu, James D Lewis, Ian Frank, Edward Cantu, Joshua M Diamond, Jason D Christie, Ronald G Collman, Frederic D Bushman

**Affiliations:** Department of Microbiology, University of Pennsylvania School of Medicine, 426 Johnson Pavilion, 3610 Hamilton Walk, Philadelphia, PA 19104 USA; Pulmonary, Allergy and Critical Care Division, University of Pennsylvania School of Medicine, 522 Johnson Pavilion, 3610 Hamilton Walk, Philadelphia, PA 19104 USA; Gastroenterology Division, University of Pennsylvania School of Medicine, 522 Johnson Pavilion, 3610 Hamilton Walk, Philadelphia, PA 19104 USA; Infectious Diseases Division, Department of Medicine, University of Pennsylvania School of Medicine, 522 Johnson Pavilion, 3610 Hamilton Walk, Philadelphia, PA 19104 USA; Department of Cardiovascular Surgery, University of Pennsylvania School of Medicine, Philadelphia, PA USA

## Abstract

**Background:**

Fungi are important pathogens but challenging to enumerate using next-generation sequencing because of low absolute abundance in many samples and high levels of fungal DNA from contaminating sources.

**Results:**

Here, we analyze fungal lineages present in the human airway using an improved method for contamination filtering. We use DNA quantification data, which are routinely acquired during DNA library preparation, to annotate output sequence data, and improve the identification and filtering of contaminants. We compare fungal communities and bacterial communities from healthy subjects, HIV+ subjects, and lung transplant recipients, providing a gradient of increasing lung impairment for comparison. We use deep sequencing to characterize ribosomal rRNA gene segments from fungi and bacteria in DNA extracted from bronchiolar lavage samples and oropharyngeal wash. Comparison to clinical culture data documents improved detection after applying the filtering procedure.

**Conclusions:**

We find increased representation of medically relevant organisms, including *Candida*, *Cryptococcus*, and *Aspergillus*, in subjects with increasingly severe pulmonary and immunologic deficits. We analyze covariation of fungal and bacterial taxa, and find that oropharyngeal communities rich in *Candida* are also rich in *mitis* group *Streptococci*, a community pattern associated with pathogenic polymicrobial biofilms. Thus, using this approach, it is possible to characterize fungal communities in the human respiratory tract more accurately and explore their interactions with bacterial communities in health and disease.

**Electronic supplementary material:**

The online version of this article (doi:10.1186/s13059-014-0487-y) contains supplementary material, which is available to authorized users.

## Background

Next-generation sequencing can provide unprecedented data on the composition of mixed microbial communities, including those important in clinical infections, but admixture of contaminating DNAs into low biomass samples provides a considerable challenge to analysis. For example, fungi can result in both colonization and severe infections of the human airway, but are often present in environmental specimens in low abundance, complicating distinguishing authentic community members from background. Here we introduce a method for abundance-weighting that improves detection of fungi, and use the method to: (1) identify fungi in clinical airway samples; and (2) quantify covariation of fungal and bacterial lineages.

Many examples of medically important interactions between individual bacteria and fungi have been reported [1-4]. For example, the fungus *Candida albicans* is known to interact with several bacteria in disease states, including with *Pseudomonas* in wound infections, with *Staphylococci* in systemic infections, and with *Streptococci* in dental caries. However, fungal-bacterial interactions have not been studied extensively using culture-independent sequence-based methods (for a few examples see [5-9]).

Here we compare a series of samples from the airway of subjects with progressively more severe immunodeficiency and/or lung disease, consisting of healthy controls, HIV+ subjects, subjects with mixed pulmonary diseases, and lung transplant recipients. Transplant requires intensive treatment with immunosuppressive drugs, and mechanical defense mechanisms are also compromised due to defective cough reflex from vagal denervation, mucociliary clearance dysfunction, and anastomotic site barriers [10-19]. The HIV+ subjects included individuals both on and off antiretroviral therapy, but most had relatively preserved immune function based on CD4 T cell counts, providing a group intermediate between healthy controls and lung transplant recipients.

In previous studies, we investigated the microbiota of the healthy lung [20] and of the lung allograft from lung transplantation subjects [21], using samples obtained by bronchoalveolar lavage (BAL). Upper respiratory communities were sampled concurrently by oropharyngeal wash (OW). We used targeted PCR amplification of 16S rDNA sequence tags to characterize bacteria and ITS1 rDNA sequence tags to characterize fungi. In healthy subjects, bacterial communities in lung are quite sparse, and dominated by lineages from the densely colonized upper respiratory tract (URT). Oropharyngeal lineages in BAL may be authentically present as a result of aspiration, but may also be introduced into BAL samples as contaminants during trans-oral bronchoscopy [20]. In lung transplant recipients, however, the lung allograft contains a higher microbial biomass, and BAL samples contain a greater proportion of distinctive lineages likely representing bacteria authentically replicating in lung [21]. BAL samples from individual transplant recipients commonly showed high levels of specific bacterial taxa including *Pseudomonas* [22], *Staphylococcus* [23], or *Achromobacter* [24], as well as the little known anaerobic lineage *Sneathia* [25]. The HIV+ subjects studied here had relatively preserved immune function, and levels of bacterial DNA were comparable to healthy controls.

A significant problem in sampling fungi is the relatively modest numbers of organisms in typical samples and the high representation of contaminating lineages in blank controls, reflecting admixture of fungal DNA from environmental sources during sample acquisition or DNA contamination in reagents. Here we introduce a method for using information on the efficiency of PCR reactions (PicoGreen quantification prior to amplicon pooling) to improve detection of authentically present fungi. An advantage of this approach is that sequencing laboratories necessarily collect the PicoGreen data as a step in sequence library preparation, so acquisition of these data requires no extra effort. Using the corrected abundances, we queried the data from healthy subjects, HIV+ subjects, and lung transplant recipients for evidence of authentic respiratory tract fungi, and for covariation of bacterial and fungal lineages. We: (1) identified multiple lineages of clinical interest enriched in the more immunocompromised subjects; and (2) found a significant covariation of *Candida* and *Streptococcus*, an interaction which has been proposed to be important in formation of medically important biofilms [26], and which we characterize in more detail here.

## Results

### Fungal diversity and community composition

Samples were obtained from healthy control subjects (n = 12), HIV+ subjects (n = 19), subjects undergoing bronchoscopy for a variety of clinical indications (n = 13), and lung transplant recipients (n = 42). Healthy and HIV+ subjects were without respiratory symptoms and had not used recent antibiotics. HIV+ subjects included untreated viremic (n = 11) and antiretroviral-treated, virologically-suppressed individuals (n = 8), with median CD4 T cell counts of 591 and 674, respectively. Lung transplant subjects were all undergoing treatment with multiple immunosuppressive drugs and combinations of antibiotics. The study groups and sampling protocols are listed in Table 1 and Additional file 1.

**Table 1:** **Groups of subjects appearing in this study**

**Group**	**Subjects (n)**	**Description**	**Median CD4 count**	**Bronchoscopy procedure**	**Reference**
1A	8	HIV+ off ART, CD4 > 400	769	Two scope	This study
1B	3	HIV+ off ART, CD4 < 400	315	Two scope	This study
2B	8	HIV+ on ART, no lung disease	674	Two scope	This study
3B	6	HIV-, no lung disease		Two scope	16S data [20], ITS data [21]
3C	4	HIV-, no lung disease		None	This study
3D	6^a^	HIV-, no lung disease		Single scope	This study
Pulm	13	HIV-, mixed lung disease		Single scope	This study
Transplant	42	Lung transplant recipients		Single scope	Time points with paired BAL and OW samples [21]^b^

^a^Four subjects in group 3D were also sampled in group 3B. In each case, the group 3D sample was taken more than 1 year after the 3B sample (see Additional file 1 for details).

^b^Sample sets Tx34B and Tx41B appear as Tx34 and Tx41, respectively, in Charlson 2012. The initial time points were not included in the previous publication because OW samples were not collected at the time of sampling.

We profiled the fungal populations of 69 oropharyngeal wash and 149 BAL samples using 454 pyrosequencing of rRNA ITS regions. The bacterial community composition was also characterized by amplifying and sequencing 16 s rRNA V1V2 regions (data for healthy subjects in group 2B and transplant recipients with paired BAL and OW samples was published previously [20,21], data for HIV+ subjects is new here). A set of 132 contamination control samples were also analyzed to enumerate the fungal DNA present in lab water (21 samples), saline solution used for OW and BAL sampling (9 samples), within the suction channel of a bronchoscope (86 samples), sterile swabs (8 samples), and lab tabletop surfaces (8 samples). The sources of contamination control samples are more fully described in Additional file 2. Analysis of contamination in 16S data is described in [20].

We recovered a median value of 798 ITS reads per sample after removal of low-quality reads. The number of reads per sample is somewhat low for deep sequencing studies, but as discussed below relatively few fungi are typically present in samples, so the read numbers are generally adequate for detection, as seen in earlier studies [21]. The number of reads per sample was significantly lower in contamination control samples compared to BAL/OW samples (Mann–Whitney test, *P* <0.001). After clustering at 97% similarity, we obtained a total of 1801 fungal operational taxonomic units (OTUs). Following taxonomic assignment, we identified 153 OTUs arising from non-fungal sources, including human, plants, and bacteria. Additionally, we flagged 77 OTUs as potential chimeras. These were removed prior to subsequent analysis.

**Figure 1:**
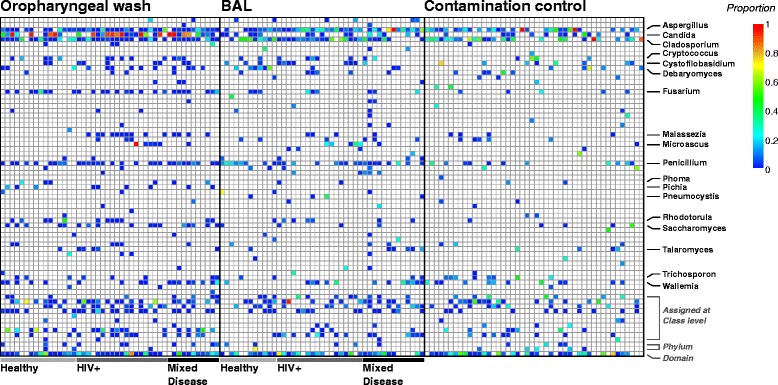
**Proportional abundance of fungal genera in oropharyngeal wash, bronchoalveolar lavage, and contamination control samples.** Many genera with high proportional abundance appear in only a few samples. For simplicity, only genera that were detected in 10 OW or 10 BAL samples are identified by name (full data in Additional file 2).

Figure 1 shows a representative heatmap of fungal proportions for all non-transplant samples in the study (data for transplant samples are in [21]). A total of 61 fungal genera are displayed, each represented by more than 100 reads counting across all samples in this set. The genus could be assigned for 65% of the OTUs, 83% could be assigned at the class level, and 90% at the phylum level.

Although the total number of fungal OTUs detected is large, the number of OTUs present in any one sample is low. At a sequencing depth of 300 reads, the average richness or number of OTUs detected in each OW/BAL sample is only 14. In contrast, bacterial communities from similar samples have a richness of about 130 OTUs at the same sequencing depth. The number of abundant fungal OTUs is also low, as indicated by the sum of the log-weighted proportions, or Shannon diversity. Shannon diversity was only weakly dependent on the total number of reads (Additional file 2), and had a mean value of 1.5 for the fungal communities in oropharyngeal and BAL samples. Comparable bacterial communities have a Shannon diversity of 3 to 4 [21].

Disturbingly, the composition of fungi in BAL samples was generally similar to that in contamination controls (Figure 1, right side). A PERMANOVA test of pairwise Jaccard or Bray-Curtis distances yielded no significant difference between BAL and contamination control samples in healthy subjects (Additional file 2) [27]. The oropharyngeal wash samples were distinguishable from contamination, largely on the basis of high *Candida* abundance, which is consistent with the well-recognized tropism of *Candida* for the oropharyngeal region [28]. Thus it is difficult to determine whether any given detection of fungal DNA in a specific sample represents authentic presence of the organism or contamination.

A further problem is that the reproducibility of fungal proportions measured in oropharyngeal and BAL samples is poor between repeat extractions from the same source. To examine this, we carried out repeated DNA extractions and ITS sequencing for bronchoscope pre-wash, OW, and BAL samples from six subjects in the Pulm and Transplant groups. In two OW and two BAL samples, a fungal OTU was observed with over 50% proportion in one replicate, but was absent from another (Additional file 2). Fungal OTUs with proportions of 1% to 50% in general appeared sporadically between replicates (further details in Additional file 2). Bacterial proportions, by contrast, are recovered with reasonable accuracy down to proportions of 10^−4^ for comparable sample types [29]. The poor reproducibility of fungal proportions would be expected for stochastic sampling of low abundance taxa derived from a combination of contaminants and authentically present lineages.

### Use of DNA quantification after ITS PCR for improved accuracy

We investigated whether absolute abundance measurements could be used to supplement the relative abundance data from pyrosequencing and address some of the above problems. Fungal quantification by qPCR primers directed at the 18S rRNA gene was impossible because the overwhelming proportion of human DNA in BAL and OW samples resulted primarily in amplification of human 18S DNA, even with fungal-targeted 18S primers (data not shown). We thus quantified fungal DNA using the ITS-specific primers used in preparing pyrosequencing libraries.

To quantify fungal templates present in a sample, we took advantage of DNA quantification data generated during routine preparation of sequencing libraries. In a typical experiment, individual airway DNA specimens are amplified by PCR using ITS primers, DNA products purified, and the PCR yield quantified using the PicoGreen dye-binding assay. Equal amounts of DNA from each sample are then pooled and introduced into the sequencing instrument. Here we used the post-PCR PicoGreen data to estimate the number of ITS templates present in the initial DNA sample prior to PCR, which could then be used to annotate the sequence proportions, yielding estimates of OTU abundance that take account of template abundance in the original airway DNA specimen. It would be possible to substitute ITS QPCR at this step with improved accuracy, but fungal ITS sequences are of variable lengths, complicating quantification, and we sought to explore what was possible using PicoGreen data on end point PCR because of the ready availability.

**Figure 2:**
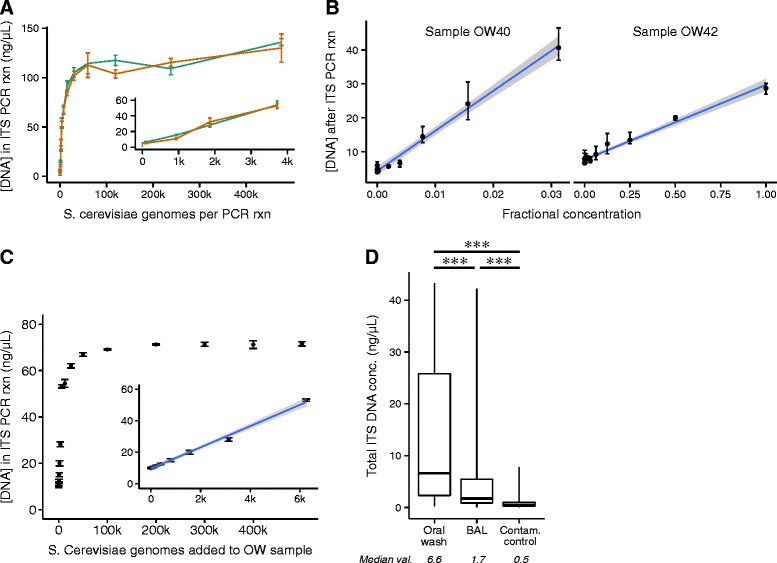
**Quantification of post-PCR ITS DNA is linear in the range of oropharyngeal, lung, and contamination control samples. (A)** Two serial dilutions of a standard sample of *S. cerevisiae* showed a linear response between input and post-PCR PicoGreen-quantification in the range yielding 0 to 60 ng/μL of ITS product. **(B)** Serial dilutions of two oropharyngeal wash samples from lung transplant recipients also showed a linear response in this range. **(C)** Genomic DNA from *S. cerevisiae* spiked into an oropharyngeal wash sample resulted in a linear increase in total DNA concentration within the 0 to 60 ng/μL range, as measured by post-PCR PicoGreen quantification. **(D)** Although the total concentration of post-PCR ITS DNA differed between sample types, the median concentration of contamination control samples was about 10% of the concentration in oropharyngeal wash. The median concentration of lung samples was only a few times that of contamination controls.

To evaluate PicoGreen quantification, we first quantified PCR product formation when dilutions of *S. cerevisiae* genomic DNA were used as amplification template (Figure 2A). Above 100 ng/μL of ITS amplicon, the PicoGreen assay was saturated. However, below values of 60 ng/μL, the response of the PicoGreen assay was linear with the number of input genomes (R^2^ = 0.98), suggesting that post-PCR ITS PicoGreen values report the amount of starting ITS templates within this range.

We next tested oropharyngeal wash samples containing biologically relevant fungal communities. Serial dilutions were performed for OW samples from two transplant subjects, followed by amplification and PicoGreen quantification. The samples were selected for study because they yielded levels of ITS product in prior analysis that were among the highest of any of our respiratory tract samples, suggesting authentic detection of fungi and allowing us to dilute through a broad range of concentrations [21]. The relationship between OW input and PicoGreen-quantified ITS product was linear in the expected range of 0 to 60 ng/μL (R^2^ > 0.97 for both samples, Figure 2B). We also measured the post-PCR ITS DNA concentration in OW samples after spiking in DNA from *S. cerevisiae*. As expected, the PicoGreen quantification was linear at concentrations below 60 ng/μL (R^2^ > 0.99, Figure 2C). Thus the ITS PicoGreen quantification is linear across a range that would be present in many respiratory tract samples.

### Quantification of ITS PCR product generated by amplifying OW, BAL, and contamination control samples

The median amount of ITS DNA produced by PCR was different for OW, BAL, and contamination control samples (Figure 2C, *P* <0.001, Kruskal-Wallis). The median for oropharyngeal wash samples was 5.6 ng/μL compared to 1.3 ng/μL for BAL samples (*P* <0.001, Mann–Whitney). The median ITS PCR product of contamination control samples, 0.69 ng/μL, was smaller than that of BAL and oropharyngeal wash samples (*P* <0.001, Mann–Whitney). Although significantly smaller, the concentration of fungal ITS DNA in contamination controls was within an order of magnitude for OW and within a factor of 4 for BAL samples. Thus a substantial fraction of ITS reads in the experimental samples likely arose from contamination sources.

### Analysis of PicoGreen-corrected OTU abundances

**Figure 3:**
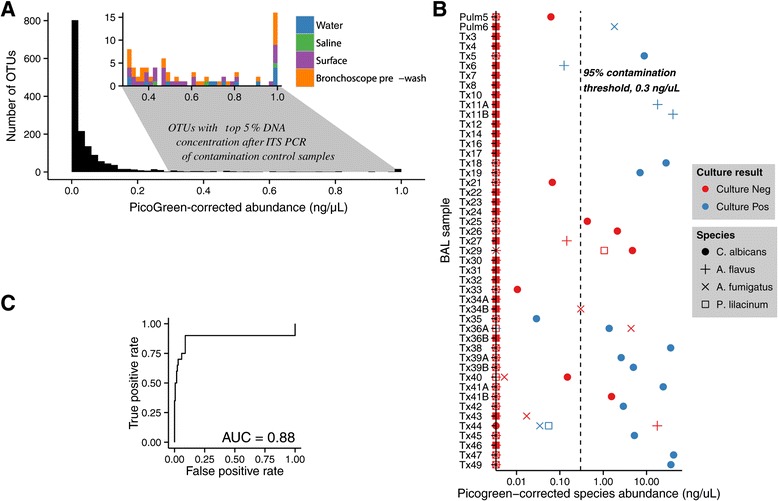
**A global threshold for PicoGreen-corrected OTU abundance identifies genera in experimental samples that are extremely unlikely to arise from contamination sources. (A)** Histogram of PicoGreen-corrected OTU abundances in control samples. The inset plot shows abundances above the 95% limit of the distribution, colored by contamination sample type. **(B)** Agreement between the PicoGreen-corrected abundance of fungi computed from ITS sequencing and clinical culture results in BAL samples. **(C)** ROC curve of post-PCR ITS abundance vs. culture results for all cultured fungi. The sequencing method is a good predictor of culture results (AUC = 0.93).

We next used the abundance data from PicoGreen quantification to transform the relative proportions determined from the pyrosequencing data. The corrected abundance of each OTU was computed by multiplying the total post-PCR ITS DNA concentration for the sample by the proportion of each OTU in the sample. To establish a global threshold for concentration of fungal OTUs, an empirical cumulative distribution function was generated from the PicoGreen-corrected OTU abundances in contamination control samples. A histogram of the abundance distribution in contamination controls is shown in Figure 3A. The 95% limit of the abundance distribution for control samples was 0.3 ng/μL. An inset plot shows the control OTUs with abundance above the 95% limit. No single contamination source dominated this set of high-abundance OTUs.

A 95% confidence threshold for abundance is somewhat conservative. We judged it to be suitable for the set of samples presented here, where we are interested in describing the set of fungi that are unlikely to arise from contamination sources. For other applications, a different threshold may be used simply by selecting a different percentile value from the distribution of OTU abundances in contamination samples.

A potential issue with the use of a global abundance threshold is that the abundance distribution of some fungal genera may differ from the global distribution. To check this, we selected all fungal genera appearing in 10 or more contamination control samples and estimated a genus-specific abundance threshold. In every case save one, the global abundance threshold fell within the 95% confidence limits of the genus-specific threshold, indicating that the genus-specific thresholds are compatible with the global threshold. The sole exception is *Cladosporium*, the most commonly occurring fungal genus in contamination control samples. We estimated a genus-specific threshold of 0.81, about three times higher than the global threshold. Thus apart from *Cladosporium*, any experimental OTU with abundance greater than 0.3 ng/μL is unlikely to arise from contamination sources.

### Application of PicoGreen-corrected OTU abundances to clinical samples

To verify that a global abundance threshold would work as intended, we first applied the global abundance threshold technique to a set of 47 BAL samples for which we had clinical culture results. The samples came from lung transplant recipients who are heavily immunosuppressed and highly susceptible to fungal colonization and infection. BAL samples were cultured using standard clinical methods. Cultures from 18 samples were positive for fungi including *Candida albicans*, *Aspergillus flavus*, *A. fumigatus*, and *Purpureocillium lilacinum* (syn. *Paecilomyces lilacinus*). After matching the OTU assignments to the cultured species, we recovered 14 of 20 positive culture results with our method (Figure 3B). Of the remaining six, two samples revealed *Aspergillus* by both ITS and culture but differed in the species identified (*A. fumigatus* by culture and *A. flavus* by ITS assignment in sample 44, and vice versa in sample 36A). In samples Tx6, Tx35, and Tx44, we detected the cultured fungus by sequencing (*A. flavus* in Tx6, *C. albicans* in Tx35, *A. fumigatus* and *P. lilacinum* in Tx44), but the PicoGreen-corrected quantity was below the contamination threshold. Sample Tx5 cultured positive for *C. albicans*, but we detected high levels of *C. dubliniensis*, a closely related species that is often not distinguished from *C. albicans* in culture [30]. We counted this as a positive result. Our method also identified four samples with high concentrations of *C. albicans* DNA that were not detected by culture.

We evaluated our ITS fungal detection method across all possible threshold values by plotting the receiver operating characteristic (ROC) curve, which displays the rate of true positives (from culture results) against the rate of false positives (Figure 3C). The area under the curve was 0.93, indicating that our method was able to detect most culture positive fungi with few false positives. We also assessed the consistency between culture and sequencing results using Cohen’s Kappa coefficient, which measures the level of agreement above that expected by chance. Our DNA sequencing and culture results had a Kappa of 0.65, indicating good agreement. This level of agreement is slightly higher than those reported in a study of bacterial culture vs. DNA sequencing methods [31]. The value of Kappa was not sensitive to the abundance threshold used (Additional file 2).

Returning to the issue of reproducibility, we re-evaluated our set of replicate samples using PicoGreen-corrected abundances. We found that use of an absolute abundance threshold improved the level of agreement between replicate samples extracted from the same source material, especially when the PicoGreen-corrected abundance is greater than 1 ng/μL (details in Additional file 2).

### Identifying fungi in oropharyngeal and BAL samples that were unlikely to originate from contamination

**Figure 4:**
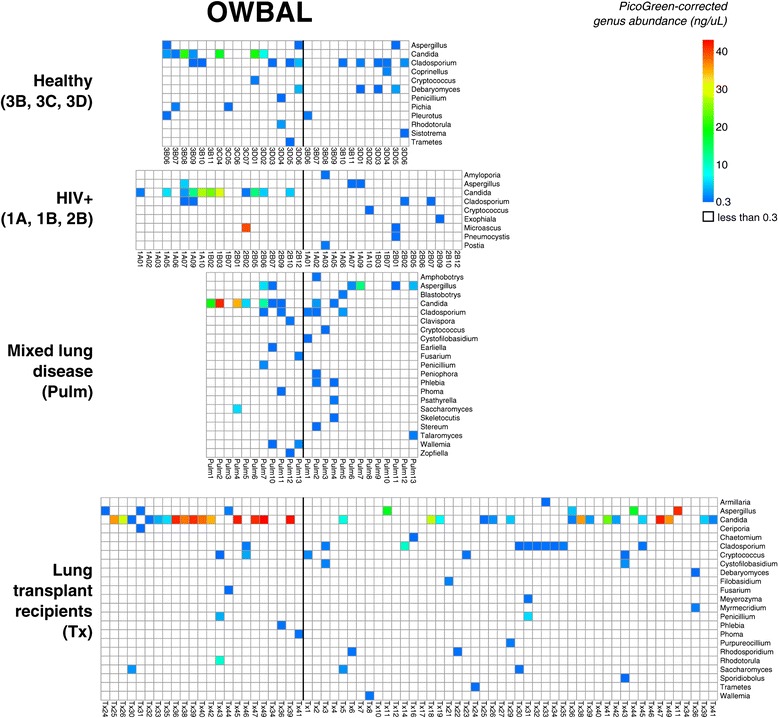
**Heatmap of fungal genera identified in a matched set of oropharyngeal and lung samples following curation by ITS abundance.**

We next applied a global abundance threshold to the OW and BAL samples from subjects in our study. Using our abundance threshold approach, we eliminated OTUs in each sample with PicoGreen-corrected abundance <0.3 ng/μL. The remaining OTUs, shown in Figure 4, are unlikely to arise from contamination sources. A more detailed set of plots is available in Additional file 2.

We identified *Candida* in an OW sample of approximately half of the healthy individuals in the study (samples 3D03-3D06 are repeat samples from individuals in group 3B, see Additional file 1). *Candida* was not present in any BAL samples from healthy individuals. The fungi identified in BAL samples from healthy subjects were taxa of little clinical importance, with the exception of *Aspergillus* in subject 3D05.

The representation of *Candida* in OW and BAL samples from HIV+ individuals was similar to that of healthy subjects, with detection in OW but not BAL, which is concordant with the frequency of clinical candidiasis in the upper respiratory tract in this population but rarity of candidiasis in the lower respiratory tract [32]. HIV+ individuals are particularly susceptible to pulmonary infection with fungi (notably *Pneumocystis, Cryptococcus, Histoplasmosis,* and *Aspergillus*) [33], though the subjects studied here were without any respiratory symptoms and had relatively well-preserved immune function (mean CD4+ count 644 ± 299). In BAL of HIV+ subjects, we detected clinical pathogens *Pneumocystis, Cryptocossus,* and *Aspergillus*. One HIV+ subject, 2B02, was found to have high levels of *Microascus* in oropharyngeal wash.

Subjects in the Pulm group were sampled while undergoing clinical bronchoscopy for a variety of indications (details in Additional file 1), and represent a varied assortment of patients with lung disease. Here, we observed *Aspergillus* in the BAL samples of four subjects, and *Cryptococcus* in another. *Candida* was observed concurrently in both OW and BAL samples of subjects Pulm2 and Pulm4.

For lung transplant recipients, also sampled during clinical bronchoscopy (data in part from [21] and in part new here), we detected *Candida* in BAL samples for 14 of the subjects, a significantly higher proportion than in non-transplant individuals (*P* <0.001, Fisher’s exact test). The abundance of *Candida* in transplant BAL samples was higher than that observed in the Pulm group, exceeding 20 ng/μL (PCR product measured by PicoGreen) in five cases (Tx18, Tx38, Tx41A, Tx47, and Tx49). All transplant subjects with *Candida* in BAL who had OW available also had *Candida* in OW, except for one (Tx41B). *Aspergillus* was detected in the BAL samples of four transplant subjects, and had an abundance in excess of 20 ng/μL in samples Tx11A, Tx11B, Tx44. The abundance of *Cryptococcus* in transplant BAL samples was similar to that observed in the Pulm group. *Cryptococcus* was observed in both OW and BAL for one subject (Tx43).

### Fungal occurrence in oropharyngeal and BAL samples relative to contamination controls

**Figure 5:**
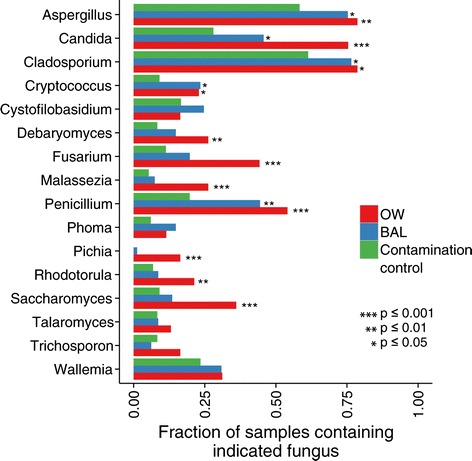
**Presence-absence analysis identifies fungal genera present more often in experimental samples relative to contamination controls.** *Aspergillus* and *Penicillium* are present significantly more often in oropharyngeal and lung samples relative to controls, while *Pichia* and *Saccharomyces* are present more often only in OW relative to controls. Conversely, *Wallemia* likely derives solely from contamination sources.

Because so many OTUs were eliminated by the application of a global abundance threshold (many of which may be authentic community members but cannot be definitively distinguished from environmental admixture), we wondered whether other methods might be able to identify less abundant genera legitimately present in OW or BAL samples. For genera where the PicoGreen-corrected abundance is not enough to call individual OTUs as authentically present in a particular sample, the frequency of occurrence can nevertheless reveal patterns across sample types. Figure 5 shows the frequency of occurrence in OW, BAL, and contamination control samples for genera present in at least 10 OW or BAL samples. An expanded plot is included in Additional file 2.

To assess differences in overall frequency of occurrence between sample types, we used a Fisher’s exact test. If the sample types were found to be different for a genus, follow-up tests were performed to determine whether the rate of occurrence in OW and BAL samples exceeded that in contamination controls. In each series of tests, we used the method of Benjamini and Hochberg to control for a false discovery rate of 5% [34]. *Aspergillus, Cladosporium, Cryptococcus*, and *Penicillium* were present more often in oropharyngeal wash and BAL samples than in control samples. *Debaryomyces, Fusarium, Malassezia, Pichia, Rohodotorula,* and *Saccharomyces* appeared more often in oropharyngeal wash samples than in controls, but did not occur significantly more often in BAL samples relative to contamination sources. *Pichia* was the sole genus present in more than 10 OW/BAL samples not to appear in any contamination control samples. The frequencies of *Cystofiloblasidum, Phoma, Talaromyces, Trichosporon*, and *Wallemia* were about the same in OW, BAL, and contamination control samples.

### Fungal covariation with bacteria

To investigate possible interactions in the human respiratory tract between microbes from different domains of life, we investigated covariation between fungi and bacteria in our samples. For this we used 16S V1V2 tag sequence data to characterize bacteria from samples used in the analysis of fungal populations described above. Bacterial 16S rRNA gene data for group 3B and some of the lung transplant samples have been published [20,21] (see Table 1 for a complete listing of data sources). The 16S rRNA tag data for bacterial communities from HIV+ subjects are newly presented here. An analysis of the HIV+ 16S rRNA tag data is presented in Additional file 3. The 16S sample set included 151 contamination control samples, which harbored bacterial communities distinct from BAL/OW samples, as expected [20]. Our analysis showed modest but statistically significant differences between HIV+ and healthy controls in OW but not BAL samples. A previously published study also showed an altered bacterial microbiome in lingual samples from HIV+ subjects [35], an oral cavity surface that closely resembles saliva [36].

**Figure 6:**
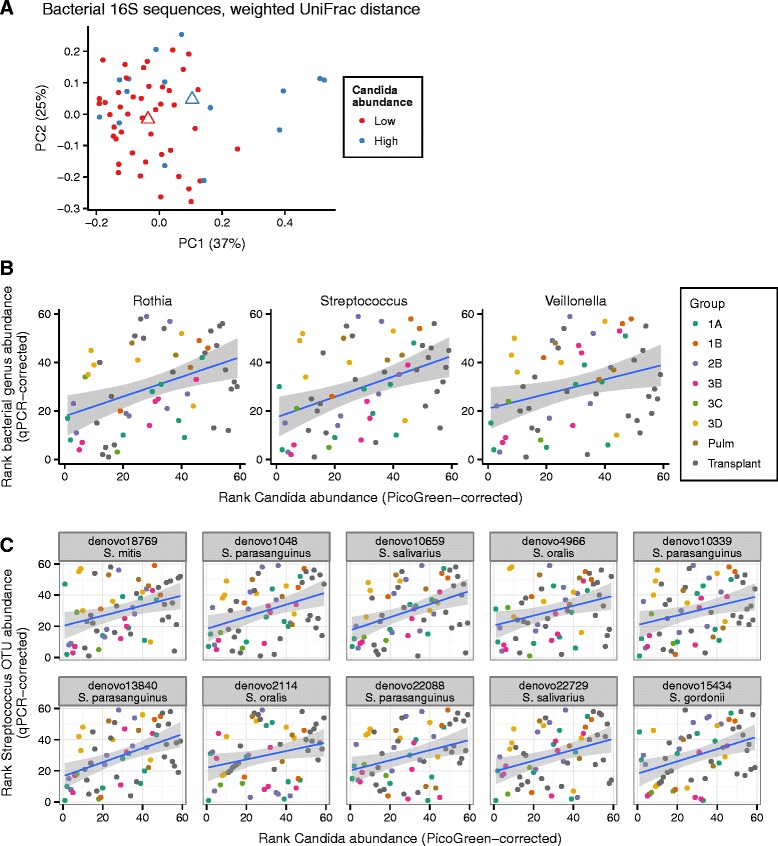
**Bacterial covariation with** ***Candida*** **in OW samples. (A)** Principal coordinates analysis of weighted UniFrac distance between bacterial communities for oropharyngeal wash samples. Group centroids are shown with open triangles. The PicoGreen-corrected abundance of *Candida* has a significant effect on bacterial community composition (PERMANOVA *P* = 0.004, F = 3.8). **(B)** The QPCR-corrected abundance of *Streptococcus*, *Rothia*, and *Veillonella* increased with the abundance of *Candida* (Spearman correlation, *P* <0.05 after FDR correction). **(C)** Ten of the top 20 most abundant *Streptococcus* OTUs were found to increase with *Candida* abundance (Spearman correlation, *P* <0.05 after FDR correction).

To assess covariation, we focused on *Candida*, which was the most commonly detected fungus in the ITS data and so provided the power needed for comparison. We analyzed the OW samples and not BAL because a greater proportion of OW samples were positive for *Candida*. We first tested for an effect of PicoGreen-corrected abundance of *Candida* on overall bacterial community composition in OW. Using an ADONIS test of pairwise UniFrac distances, we found a significant association of *Candida* PicoGreen-corrected abundance with bacterial community composition (weighted UniFrac *P* = 0.003, R^2^ = 0.06; unweighted UniFrac *P* = 0.0003, R^2^ = 0.03). Bacterial communities also varied significantly in association with high or low abundance of *Candida*, dividing at the third quartile of *Candida* abundance (Figure 6A; weighted UniFrac *P* =0.004, R^2^ = 0.05; unweighted UniFrac *P* = 0.0003, R^2^ = 0.03).

Previous culture-based studies have suggested that *Candida* and *Streptococcus* are tightly associated in the human oropharyngeal cavity [26], providing a specific hypothesis to investigate in the metagenomic data. To compare the abundance of *Candida* and *Streptococcus*, we multiplied the bacterial OTU proportions by the total 16S DNA abundance obtained by QPCR. The abundance of *Streptococcus* increased with that of *Candida* (Spearman correlation ρ = 0.41, *P* <0.001). Figure 6B shows the abundance of *Streptococcus* in each OW sample after transformation by ranks, plotted against rank-transformed *Candida* abundance. We investigated the top 20 most abundant *Streptococcus* OTUs to look for trends at the species level. Allowing for a false discovery rate of 5%, we found that 10 of the 20 increased with *Candida* abundance in a one-sided test of Spearman correlation. Eight were identified as *mitis* group Streptococci, and two were identified as *S. salivarius* (Additional file 3). One *Streptococcus* (*S. gordonii*, a member of the *mitis* lineage) showed a positive association with *Candida* and was enriched in HIV+ OW samples compared to OW from healthy controls. An exploratory analysis of effects on other bacteria showed that *Rothia* and *Veillonella* increased significantly with *Candida* abundance after controlling for a false discovery rate of 5%.

## Discussion

Microbial community analysis by deep sequencing reports relative proportions of microbes present in a sample regardless of absolute abundance. Problems arise when samples contain varying amounts of authentic organisms and a substantial background of contaminating sequences, as is commonly the case in respiratory tract samples [20]. Here, we report that the majority of fungal OTUs observed in sequence data from OW and BAL samples cannot be definitively established as authentic sample constituents because of the admixture of fungal DNA from contaminating sources. However, OTUs authentically present in experimental samples may be identified more accurately by using data on PCR amplification efficiency to annotate OTU proportions by absolute abundance. This was validated by comparing OTU calls to clinical culture results, showing improved correlation after abundance-based correction. Abundance-based correction also greatly improved agreement between technical replicates from single samples. Thus, use of an absolute abundance threshold provides a way to go beyond statistical, population-level approaches into a more diagnostic framework, allowing identification of specific fungi in a single sample with known confidence.

Our data show a progression of colonization by clinically relevant fungi in BAL across the four cohorts, from the least colonization in healthy controls, through increasing colonization from well suppressed HIV+, to mixed lung diseases (Pulm), to lung transplant recipients. In the healthy controls, only low levels of fungal reads were detected, and only one was a candidate for clinically significant colonization (*Aspergillus*). In the HIV+ group, levels were relatively low, but identification of organisms of clinical importance in BAL was greater (4 out of 18 subjects; two *Aspergillus*, one *Cryptococcus*, and one *Pneumocystis*). For the mixed lung disease group (Pulm), fungi appeared at higher levels in some cases, and organisms of potential clinical import were detected in seven out of 13 subjects (four *Aspergillus*, two *Candida*, and one *Cryptococcus*). For lung transplant recipients, the inferred abundance levels were far greater in some specimens, and 21 out of 42 individuals showed organisms of potential importance (4 *Aspergillus*, 14 *Candida*, 3 *Cryptococcus* and 1 *Purpureocillium*; one sample contained two of these). These data, together with the positive correlation with clinical diagnostic data, support the use of the abundance-weighted method for identification of fungi of clinical interest. Although this approach can establish confidence that an organism is genuinely present in a sample and does not derive from environmental admixture, it cannot distinguish possible community mixing, such as upper respiratory admixture with lung samples, which must be addressed with other measures [20,21].

The fungal sequence data could be analyzed either by scanning for authentic colonization using the abundance-weighed method, or by the use of a more sensitive but more contamination prone presence-absence approach. The advantage of the presence-absence approach is that less stringent thresholds can be used for calling enrichment in one sample type over another, allowing more frequent calling of positive samples. However, given substantial contamination, one does not know which detections in experimental samples are authentic and which represent contaminants. *Aspergillus*, for example, was found in over half of the contamination control samples, but approximately 75% of experimental samples. Thus it is likely that *Aspergillus* was authentically present in many experimental samples, but it is usually not possible to determine which ones except in those few cases where levels were particularly high. Using the presence-absence method, a total of 11 fungal genera were found to be significantly more common in either OW or BAL than in contamination controls.

Several of the fungi studied were enriched in OW samples but not BAL, which contrasts with findings for bacteria. Published comparisons of bacteria in OW and BAL in healthy controls showed that communities in the two body sites were mostly indistinguishable (once the greater representation of contamination in lung samples was properly accounted for) [20]. Samples from lung transplant recipients differed, with many examples of selective bacterial lung colonization [21]. For the fungi studied here, multiple lineages were notably enriched in OW compared to BAL, including *Candida, Debaryomyces, Fusarium, Pichia, Rhodotorula*, and *Saccharomyces*, suggesting more ‘localized’ colonization by these organisms in the oral cavity, possibly reflecting a more surface-associated lifestyle.

Comparison of abundance-corrected fungal ITS data and bacterial 16S data enabled us to assess covariation of the two. Only upper respiratory tract samples had sufficient fungal detection to allow statistical analysis, and only *Candida* was sufficiently abundant for study. Previous literature documents a variety of interactions between *Candida* and *Streptococci* in the *mitis* group: (1) *S. oralis* and *S. parasanguinis* were reported to grow together with *C. albicans* in oral biofilms [26]; (2) *S. oralis* was recently shown to enhance the virulence of *C. abicans* in a mouse model by increasing the mucosal inflammatory response [37]; and (3) differences in salivary flow were reported to change the ratio of *Candida* to *Streptococcus* in the oral cavity [38]. Using the method of PicoGreen-corrected abundance for fungal species, we were able to detect association between *Candida* and bacteria in the *S. mitis* group in our deep sequencing data. We also found linkage between *Candida* and another *Streptococcus* species outside of the *mitis* group, *S. salivarius*. A recent culture-based study observed *S. salivarius*-*C. albicans* association and found that *S. salivarius* may interfere with the ability of *C. albicans* to adhere to surfaces, thereby reducing the potential for development of candidiasis [39]. One Streptococcus lineage was found to be both enriched in OW in HIV+ versus healthy controls and positively associated with *Candida*, suggesting a possible specific interaction in the setting of HIV infection. We also found that *Rothia* and *Veillonella* covaried with *Candida* at the genus level, helping to specify a larger community pattern associated with high *Candida* abundance.

## Conclusions

The abundance-weighted sequence-based approach has several advantages compared to traditional culture based methods used in clinical diagnosis. The sequence-based method provides an overview of all community members in a single assay, without any requirement for assaying organisms individually. The abundance-weighted sequence-based method is potentially quite rapid given sufficient optimization at each analytical step, though some technology improvements are still needed. To use this approach, every laboratory must establish its own empirical contamination control threshold, since the efficiency of amplification will be affected by many laboratory-specific variables. Thus for these and other reasons collection of extensive contamination control data is essential. These data taken together indicate that the use of abundance-weighted sequence-based identification may be attractive for use in clinical diagnosis of fungi.

## Materials and methods

### Samples

Subjects studied are enumerated in Table 1 and Additional file 1. Healthy volunteers and asymptomatic HIV+ subjects who were without lung disease, acute respiratory symptoms, or recent antibiotic use underwent upper respiratory tract sampling by oropharyngeal wash (OW) followed by bronchoscopy with bronchoalveolar lavage (BAL). Sampling of healthy subjects has been previously described [20], and HIV+ volunteers were sampled in an identical manner. For subjects where multiple BAL samples were available [20], the BAL A second return sample was used. Healthy volunteers in group 3C were sampled by OW only. Healthy volunteers in group 3D were sampled in the same manner as other healthy subjects [20], but a single bronchoscope was used for the procedure. Lung transplant recipients routinely undergo surveillance bronchoscopy and BAL, and a portion of clinical BAL fluid as well as OW obtained immediately prior to bronchoscopy were collected as described [21]. Subjects in the Pulm group were sampled during clinically-indicated bronchoscopies in the same manner as lung transplant subjects. Environmental contamination controls included sterile saline used for OW and BAL sampling, saline washed through the channel of sterile bronchoscopes, and lab water mock-purified as previously described [20,21].

Written informed consent was granted by all subjects in the study. Protocols were approved by the Institutional Review Board of the University of Pennsylvania (protocols #810501 and 812748).

### 454 sequencing

To profile fungal communities, genomic DNA was extracted from whole neat BAL or OW using the PowerSoil DNA isolation kit (MoBio, Carlsbad, CA, USA) as previously described [40]. ITS genes were amplified in triplicate using barcoded ITS1F/ITS2 primers [40]. ITS amplicons were purified using Agencourt AmPure XP beads (Beckman-Colter, Brea, CA, USA) to remove primer dimers and short amplicons before PicoGreen quantification. Following this, 454 pyrosequencing was performed as previously described [40].

### Absolute DNA quantification

A PicoGreen assay was used to quantify all PCR products prior pooling for pyrosequencing. Following the protocols for DNA extraction and PCR amplification [40], 10 uL of PCR product was used for the quantification.

To determine the relationship between fungal DNA concentration in PCR reactions and PicoGreen fluorescence measured on PCR products, we assayed a dilution series of *S. cerevisiae* genomic DNA where each dilution was PCR amplified in triplicate and quantified with PicoGreen. The starting concentration of *S. cerevisiae* genomic DNA was 0.625 ng/μL, as measured by PicoGreen. Dilutions from the stock concentration were made in steps of 1:2, down to a 1:1,024 dilution. Following that, a 1:10 dilution series was made from 1:10 k to 1:1 M. Each dilution was PCR amplified in triplicate using Accuprime PCR reagents and the ITS primer pair described above. The resulting PCR products were quantified using PicoGreen at a 1:100 dilution. Two such dilution series were made to examine reproducibility. We then chose two oropharyngeal wash samples that contained high levels of ITS amplicon by PicoGreen and followed the same protocol for serial dilution and PCR amplification for each dilution. The resulting PCR products were quantified using PicoGreen at a 1:20 or 1:50 dilution. We also conducted a spiking experiment where increasing amounts of purified *S. cerevisiae* genomic DNA were added to an oropharyngeal wash sample.

### Bioinformatic analysis

Bioinformatic analysis of sequence tag data was carried out using the QIIME pipeline ver. 1.8 [41], followed by statistical analysis in the R programming language [42].

For the ITS amplicon, reads were filtered to remove sequences shorter than 130 bp, sequences with a homopolymer run exceeding 6 bp, and sequences with more than two ambiguous base calls (Ns). To be included in the analysis, reads were also required to match the sample-specific barcode sequence and the leading ITS primer sequence exactly. The reads were clustered by similarity at the 97% level using UCLUST [43]. Taxonomic assignments were generated by BROCC [40], after BLASTing representative ITS sequences to the nt database from NCBI (maximum e-value of 10^−5^, maximum 100 results per query).

For the 16S amplicon, reads were similarly filtered, but a minimum length of 200 bp was required. Taxonomic assignments were generated with the UCLUST-based consensus assignment method in QIIME ver. 1.8, using the GreenGenes taxonomy rev. 13_8 [44,45]. To compute pairwise UniFrac distances, the 16S reads were aligned with PyNAST [46] and a phylogenetic tree was estimated with FastTree 2 [47].

### Data availability

All sequence data in this paper are deposited in the NCBI Sequence Read Archive (SRA), under accession numbers SRA: SRP044188, SRA: SRP014819, and SRA: SRP014803. Data and programs used to generate the results are available under an open-source license at https://github.com/kylebittinger/airway-fungi-paper.

## Additional files

Additional file 1:
**Detailed listing of subjects, including disease status, medications, and bacterial/fungal culture results.**
Additional file 2:
**Analysis of fungal communities.**
Additional file 3:
**Analysis of bacterial communities, covariation with** ***Candida*** **.**

## Abbreviations

BAL: Bronchoalveolar lavage
OTU: Operational taxonomic unit
OW: Oropharyngeal wash
ROC: Receiver operating characteristic
URT: Upper respiratory tract

## References

[1] de Macedo JL, Santos JB (2005). Bacterial and fungal colonization of burn wounds. Mem Inst Oswaldo Cruz.

[2] Klotz SA, Chasin BS, Powell B, Gaur NK, Lipke PN (2007). Polymicrobial bloodstream infections involving Candida species: analysis of patients and review of the literature. Diagn Microbiol Infect Dis.

[3] de Carvalho FG, Silva DS, Hebling J, Spolidorio LC, Spolidorio DM (2006). Presence of mutans streptococci and Candida spp. in dental plaque/dentine of carious teeth and early childhood caries. Arch Oral Biol.

[4] Mason KL, Erb Downward JR, Mason KD, Falkowski NR, Eaton KA, Kao JY, Young VB, Huffnagle GB (2012). Candida albicans and bacterial microbiota interactions in the cecum during recolonization following broad-spectrum antibiotic therapy. Infect Immun.

[5] Barott KL, Rodriguez-Brito B, Janouskovec J, Marhaver KL, Smith JE, Keeling P, Rohwer FL (2011). Microbial diversity associated with four functional groups of benthic reef algae and the reef-building coral Montastraea annularis. Environ Microbiol.

[6] Vega Thurber R, Willner-Hall D, Rodriguez-Mueller B, Desnues C, Edwards RA, Angly F, Dinsdale E, Kelly L, Rohwer F (2009). Metagenomic analysis of stressed coral holobionts. Environ Microbiol.

[7] Dollive S, Chen YY, Grunberg S, Bittinger K, Hoffmann C, Vandivier L, Cuff C, Lewis JD, Wu GD, Bushman FD (2013). Fungi of the murine Gut: episodic variation and proliferation during antibiotic treatment. PLoS One.

[8] Hoffmann C, Dollive S, Grunberg S, Chen J, Li H, Wu GD, Lewis JD, Bushman FD (2013). Archaea and fungi of the human gut microbiome: correlations with diet and bacterial residents. PLoS One.

[9] Mukherjee PK, Chandra J, Retuerto M, Sikaroodi M, Brown RE, Jurevic R, Salata RA, Lederman MM, Gillevet PM, Ghannoum MA (2014). Oral mycobiome analysis of HIV-infected patients: identification of Pichia as an antagonist of opportunistic fungi. PLoS Pathog.

[10] Atkins BZ, Trachtenberg MS, Prince-Petersen R, Vess G, Bush EL, Balsara KR, Lin SS, Davis RD (2007). Assessing oropharyngeal dysphagia after lung transplantation: altered swallowing mechanisms and increased morbidity. J Heart Lung Transplant.

[11] Robertson AG, Griffin SM, Murphy DM, Pearson JP, Forrest IA, Dark JH, Corris PA, Ward C (2009). Targeting allograft injury and inflammation in the management of post-lung transplant bronchiolitis obliterans syndrome. Am J Transplant.

[12] Botha P, Archer L, Anderson RL, Lordan J, Dark JH, Corris PA, Gould K, Fisher AJ (2008). Pseudomonas aeruginosa colonization of the allograft after lung transplantation and the risk of bronchiolitis obliterans syndrome. Transplantation.

[13] Gottlieb J, Mattner F, Weissbrodt H, Dierich M, Fuehner T, Strueber M, Simon A, Welte T (2009). Impact of graft colonization with gram-negative bacteria after lung transplantation on the development of bronchiolitis obliterans syndrome in recipients with cystic fibrosis. Respir Med.

[14] Gottlieb J, Schulz TF, Welte T, Fuehner T, Dierich M, Simon AR, Engelmann I (2009). Community-acquired respiratory viral infections in lung transplant recipients: a single season cohort study. Transplantation.

[15] Khalifah AP, Hachem RR, Chakinala MM, Schechtman KB, Patterson GA, Schuster DP, Mohanakumar T, Trulock EP, Walter MJ (2004). Respiratory viral infections are a distinct risk for bronchiolitis obliterans syndrome and death. Am J Respir Crit Care Med.

[16] Kotsimbos TC, Snell GI, Levvey B, Spelman DW, Fuller AJ, Wesselingh SL, Williams TJ, Ostergaard L (2005). Chlamydia pneumoniae serology in donors and recipients and the risk of bronchiolitis obliterans syndrome after lung transplantation. Transplantation.

[17] Vos R, Vanaudenaerde BM, De Vleeschauwer SI, Van Raemdonck DE, Dupont LJ, Verleden GM (2008). De novo or persistent pseudomonal airway colonization after lung transplantation: importance for bronchiolitis obliterans syndrome?. Transplantation.

[18] Weigt SS, Elashoff RM, Huang C, Ardehali A, Gregson AL, Kubak B, Fishbein MC, Saggar R, Keane MP, Lynch JP, Zisman DA, Ross DJ, Belperio JA (2009). Aspergillus colonization of the lung allograft is a risk factor for bronchiolitis obliterans syndrome. Am J Transplant.

[19] Christie JD, Edwards LB, Kucheryavaya AY, Benden C, Dobbels F, Kirk R, Rahmel AO, Stehlik J, Hertz MI (2011). The Registry of the International Society for Heart and Lung Transplantation: Twenty-eighth Adult Lung and Heart-Lung Transplant Report–2011. J Heart Lung Transplant.

[20] Charlson ES, Bittinger K, Haas AR, Fitzgerald AS, Frank I, Yadav A, Bushman FD, Collman RG (2011). Topographical continuity of bacterial populations in the healthy human respiratory tract. Am J Respir Crit Care Med.

[21] Charlson ES, Diamond JM, Bittinger K, Fitzgerald AS, Yadav A, Haas AR, Bushman FD, Collman RG (2012). Lung-enriched organisms and aberrant bacterial and fungal respiratory microbiota after lung transplant. Am J Respir Crit Care Med.

[22] Williams BJ, Dehnbostel J, Blackwell TS (2010). Pseudomonas aeruginosa: host defence in lung diseases. Respirology.

[23] Tacconelli E, De Angelis G (2009). Pneumonia due to methicillin-resistant Staphylococcus aureus: clinical features, diagnosis and management. Curr Opin Pulm Med.

[24] De Baets F, Schelstraete P, Van Daele S, Haerynck F, Vaneechoutte M (2007). Achromobacter xylosoxidans in cystic fibrosis: prevalence and clinical relevance. J Cyst Fibros.

[25] Collins MD, Hoyles L, Tornqvist E, von Essen R, Falsen E (2001). Characterization of some strains from human clinical sources which resemble “Leptotrichia sanguinegens”: description of Sneathia sanguinegens sp. nov., gen. nov. Syst Appl Microbiol.

[26] Metwalli KH, Khan SA, Krom BP, Jabra-Rizk MA (2013). Streptococcus mutans, Candida albicans, and the human mouth: a sticky situation. PLoS Pathog.

[27] Anderson MJ (2001). A new method for non-parametric multivariate analysis of variance. Austral Ecol.

[28] Gorbach SL, Bartlett JG, Blacklow NR (1998). Infectious Diseases.

[29] Charlson ES, Bittinger K, Chen J, Diamond JM, Li H, Collman RG, Bushman FD (2012). Assessing bacterial populations in the lung by replicate analysis of samples from the upper and lower respiratory tracts. PLoS One.

[30] Coronado-Castellote L, Jiménez-Soriano Y (2013). Clinical and microbiological diagnosis of oral candidiasis. J Clin Exp Dent.

[31] Lindsay B, Pop M, Antonio M, Walker AW, Mai V, Ahmed D, Oundo J, Tamboura B, Panchalingam S, Levine MM, Kotloff K, Li S, Magder LS, Paulson JN, Liu B, Ikumapayi U, Ebruke C, Dione M, Adeyemi M, Rance R, Stares MD, Ukhanova M, Barnes B, Lewis I, Ahmed F, Alam MT, Amin R, Siddiqui S, Ochieng JB, Ouma E (2013). Survey of culture, goldengate assay, universal biosensor assay, and 16S rRNA Gene sequencing as alternative methods of bacterial pathogen detection. J Clin Microbiol.

[32] Greenspan D, Greenspan J (1996). HIV-related oral disease. Lancet.

[33] Benito N, Moreno A, Miro J, Torres A (2012). Pulmonary infections in HIV-infected patients: an update in the 21st century. Eur Respir J.

[34] Benjamini Y, Hochberg Y (1995). Controlling the false discovery rate: a practical and powerful approach to multiple testing. J R Stat Soc B.

[35] Dang AT, Cotton S, Sankaran-Walters S, Li CS, Lee CY, Dandekar S, Paster BJ, George MD (2012). Evidence of an increased pathogenic footprint in the lingual microbiome of untreated HIV infected patients. BMC Microbiol.

[36] Segata N, Haake SK, Mannon P, Lemon KP, Waldron L, Gevers D, Huttenhower C, Izard J (2012). Composition of the adult digestive tract bacterial microbiome based on seven mouth surfaces, tonsils, throat and stool samples. Genome Biol.

[37] Xu H, Sobue T, Thompson A, Xie Z, Poon K, Ricker A, Cervantes J, Diaz PI, Dongari-Bagtzoglou A (2013). Streptococcal co-infection augments Candida pathogenicity by amplifying the mucosal inflammatory response. Cell Microbiol.

[38] Areias C, Sampaio-Maia B, Pereira ML, Azevedo A, Melo P, Andrade C, Scully C (2012). Reduced salivary flow and colonization by mutans streptococci in children with Down syndrome. Clinics.

[39] Ishijima SA, Hayama K, Burton JP, Reid G, Okada M, Matsushita Y, Abe S (2012). Effect of Streptococcus salivarius K12 on the in vitro growth of Candida albicans and its protective effect in an oral candidiasis model. Appl Environ Microbiol.

[40] Dollive S, Peterfreund GL, Sherrill-Mix S, Bittinger K, Sinha R, Hoffmann C, Nabel CS, Hill DA, Artis D, Bachman MA, Custers-Allen R, Grunberg S, Wu GD, Lewis JD, Bushman FD (2012). A tool kit for quantifying eukaryotic rRNA gene sequences from human microbiome samples. Genome Biol.

[41] Caporaso JG, Kuczynski J, Stombaugh J, Bittinger K, Bushman FD, Costello EK, Fierer N, Pena AG, Goodrich JK, Gordon JI, Huttley GA, Kelley ST, Knights D, Koenig JE, Ley RE, Lozupone CA, McDonald D, Muegge BD, Pirrung M, Reeder J, Sevinsky JR, Turnbaugh PJ, Walters WA, Widmann J, Yatsunenko T, Zaneveld J, Knight R (2010). QIIME allows analysis of high-throughput community sequencing data. Nat Methods.

[42] R Core Team (2013). R: A Language and Environment for Statistical Computing.

[43] Edgar RC (2010). Search and clustering orders of magnitude faster than BLAST. Bioinformatics.

[44] Werner JJ, Koren O, Hugenholtz P, DeSantis TZ, Walters WA, Caporaso JG, Angenent LT, Knight R, Ley RE (2012). Impact of training sets on classification of high-throughput bacterial 16 s rRNA gene surveys. ISME J.

[45] McDonald D, Price M, Goodrich J, Nawrocki E, DeSantis T, Probst A, Andersen G, Knight R, Hugenholtz P (2012). An improved Greengenes taxonomy with explicit ranks for ecological and evolutionary analyses of bacteria and archaea. ISME J.

[46] Caporaso JG, Bittinger K, Bushman FD, DeSantis TZ, Andersen GL, Knight R (2010). PyNAST: a flexible tool for aligning sequences to a template alignment. Bioinformatics.

[47] Price M, Dehal P, Arkin A (2010). FastTree 2-approximately maximum-likelihood trees for large alignments. PLoS One.

