# Supplementary material for: Improved characterization of medically relevant fungi in the human respiratory tract using next-generation sequencing
Source: Genome Biol. 2014 Oct 25;15(10):487. doi: 10.1186/s13059-014-0487-y (PMC4232682; doi:10.1186/s13059-014-0487-y)
Supplement: Additional file 1: — Detailed listing of subjects, including disease status, medications, and bacterial/fungal culture results. [file 13059_2014_487_MOESM1_ESM.pdf]

Table S1: Detailed listing of subjects for groups 1A, 1B, 2B, 3B, 3C, and 3D.

Group	Subject	Age	Sex	Smoking status	CD4 count	Culture results from oropharyngeal swab	Culture results from BAL fluid	Culture results from BAL PSB	Antiretroviral medications	Antibiotics, past 3 months	Inhaler use, current	Inhaler use, past 3 months	Previous subject ID
1A	1A01	48	Male	Smoker	985	Normal flora	Normal flora	Normal flora					
	1A02	43	Female	Smoker	1192	Normal flora	Normal flora	NA					
	1A03	43	Male	Nonsmoker	682	Normal flora	No growth	NA					
	1A05	41	Female	Smoker	856	Normal flora	NA	Normal flora					
	1A06	59	Female	Smoker	461	Enteric gram negative rods	Normal flora	Enteric gram negative rods					
	1A07	32	Male	Nonsmoker	948	Group A Streptococcus, normal flora	Group A Streptococcus, normal flora	Group A Streptococcus, normal flora					
	1A09	60	Male	Smoker	591	Normal flora	Pseudomonas aeruginosa, normal flora	Normal flora					
	1A10	39	Female	Nonsmoker	464	Normal flora	NA	NA			Advair	Advair	
1B	1B02	23	Male	Smoker	315	Enterobacteraceae, normal flora	NA	NA					
	1B03	26	Female	Nonsmoker	301	Saccharomyces cerevisiae	Normal flora	Beta-hemolytic Streptococcus, normal flora					
	1B07	25	Female	Nonsmoker	321	Staphylococcus aureus, normal flora	No growth	No growth		Clindamycin		Yes, medication unknown	
2B	2B01	43	Male	Smoker	609	Normal flora	NA	NA	Abacavir/Lamivudine, Ritonavir, Atazanavir				
	2B02	41	Female	Smoker	739	Normal flora	NA	NA	Emtricitabine/Tenofovir, Ritonavir, Atazanavir				
	2B05	52	Female	Smoker	868	Normal flora	Normal flora	NA	Emtricitabine/Tenofovir, Ritonavir, Atazanavir				
	2B06	42	Female	Smoker	1138	Normal flora	Normal flora	Normal flora	Abacavir/Lamivudine, Lopinavir/Ritonavir				
	2B07	47	Male	Smoker	224	Normal flora	Normal flora	Normal flora	Emtricitabine/Tenofovir, Lopinavir/Ritonavir				
	2B09	44	Male	Smoker	510	Actinomyces, Streptococcus, normal flora	Group B Streptococcus, normal flora	Normal flora	Emtricitabine/Tenofovir, Raltegravir				
	2B10	55	Male	Smoker	771	Normal flora	Streptococcus pneumoniae, normal flora	Streptococcus pneumoniae, normal flora	Abacavir/Lamivudine, Efavirenz				
	2B12	52	Female	Nonsmoker	269	Enteric gram negative rods	Normal flora	Normal flora	Efavirenz/Emtricitabine/ Tenofovir				
3B	3B06	40	Male	Smoker		Normal flora	Normal flora	Normal flora					
	3B07	25	Female	Nonsmoker		Enterobacteracae, Haemophilus, S. aureus, normal flora	Normal flora	Normal flora					
	3B08	32	Male	Nonsmoker		Haemophilus, normal flora	Normal flora	Normal flora					
	3B09	43	Male	Smoker		Group B Streptococcus	Normal flora	NA					
	3B10	66	Male	Nonsmoker		Normal flora	Normal flora	Normal flora					
	3B11	51	Female	Smoker		Normal flora	No growth	No growth					
3C	3C04	NA	Male	Nonsmoker		Streptococcus, normal flora	NA	NA					
	3C05	NA	Male	Nonsmoker		S. aureus, normal flora	NA	NA					
	3C06	NA	Male	Nonsmoker		Normal flora	NA	NA					
	3C07	NA	Male	Nonsmoker		Normal flora	NA	NA					
3D	3D01	48	Female	Smoker		S. aureus, normal flora	No growth	S. aureus, normal flora					
	3D02	38	Female	Nonsmoker		No growth	Normal flora	Normal flora					
	3D03	52	Female	Smoker		Group A Streptococcus, normal flora	Normal flora	Normal flora					3B11
	3D04	33	Male	Nonsmoker		Normal flora	Normal flora	No growth					3B08
	3D05	67	Male	Nonsmoker		Normal flora	Normal flora	No growth					3B10
	3D06	42	Male	Smoker		Normal flora	No growth	Normal flora				Albuterol	3B06

Table S2: Detailed listing of subjects in the Pulm group.

Subject	Age	Sex	Disease status	Culture results from BAL fluid	Antivirals	Antimicrobials	Immunosuppression	Notes
Pulm 1	48	F	Sarcoid	None	None	None	None	Low 16S qPCR
Pulm 2	69	M	Heart transplant	Pseudomonas, Candida albicans	None	TMP/S	Pred, Tac, MTX	
Pulm 3	48	M	Lung adenocarcinoma	None	None	None	None	
Pulm 4	54	F	BOOP	Staphylococcus aureus, Proteus mirabilis	None	None	None	
Pulm 5	89	F	Pneumonia	Pseudomonas, Haemophilus influenzae	None	None	None	
Pulm 6	91	F	Pneumonia	Aspergillus fumigatus	None	None	None	
Pulm 7	60	F	Bronchiectasis	Streptococcus pneumoniae, Aspergillus terreus	Valacyclovir	None	None	
Pulm 8	55	M	Beryllium exposure		None	None	None	
Pulm 9	50	F	Sarcoid		None	None	None	
Pulm 10	67	F	Sarcoid		None	None	None	
Pulm 11	50	M	Sarcoid	Methicillin-resistant S. aureus	None	None	None	
Pulm 12	48	F	Sarcoid		None	None	Etanercept, MTX	
Pulm 13	61	M	Hypersensitivity pneumonitis	Klebsiella oxytoca	None	Minocycline	None	

Table S3: Detailed listing of subjects in the Transplant group.

Subject	Sample	Sex	Pretransplant disease	CMV status (D/R)	Transplant type	Months after transplant	A grade at bronch	Age at bronch	Bronch purpose	Immunosuppresion	Antimicrobials	BAL bacterial culture	BAL fungal culture
Tx1*	1	--	--	--	--	--	--	--	--	--	--	--	--
Tx2*	2	--	--	--	--	--	--	--	--	--	--	--	--
Tx3	3	F	CF	P/N	B	2	0	48	Clinical	Tac, Pred, Aza	Valgan, Doxycycline, TMP/S, Meropenem	Pseudomonas	
Tx4	4	M	IPF	P/P	S-R	7	0	66	Routine	Cellcept, Tac, Pred	Nystatin, TMP/S		
Tx5	5	M	A1AT	P/N	B	3	0	49	Routine	Aza, Pred, Tac	Atovaquone, Valgan	Staphylococcus aureus	Candida albicans
Tx6	6	M	RA-ILD	P/N	B	3	2	54	Routine	Aza, Pred, Tac	Nystatin, TMP/S, Valgan		Aspergillus flavus
Tx7	7	M	CF	P/P	B	13	0	41	Routine	Cellcept, Pred, Tac	Azithro, TMP/S		
Tx8	8	M	IPF	N/N	S-L	1	2	61	Routine	Cellcept, Pred, Tac	TMP/S, Valcyc, Nystatin	S. aureus	
Tx10	10	M	IPF	N/P	S-L	13	0	64	Routine	Aza, Pred, Tac	TMP/S		
Tx11	11A	M	COPD	N/N	B	1	0	57	Routine	Aza, Pred, Tac	Valcyc, TMP/S, Nystatin		A. flavus
	11B			N/N		2	0	57	Routine	Aza, Pred, Tac	Vori, Valcyc, TMP/S		A. flavus
Tx12	12	F	Bronchiectasis	N/P	B	21	0	47	Routine / Clinical	Cellcept, Pred, Tac	Azithro, TMP/S, Valgan		
Tx14	14	F	ILD	N/N	S-L	1	0	63	Routine	Aza, Pred, Tac	Atovaquone, Keflex, Nystatin, Valcyc		
Tx16	16	M	IPF	N/N	B	7	0	59	Routine	Aza, Pred, Tac	Valcyc, TMP/S		
Tx17	17	M	PCD	P/N	B	4	0	32	Routine / Clinical	Aza, Pred, Tac	Valgan, TMP/S		
Tx18	18	F	COPD	P/P	S-L	2	0	57	Routine	Cellcept, Pred, Tac	Atovaquone, Valgan, Nystatin		C. albicans
Tx19	19	M	RA-ILD	P/N	B	12	0	49	Routine	Aza, Pred, Tac	TMP/S	Type A Haemophilus influenzae	C. albicans
Tx21	21	M	COPD	P/N	B	2	0	41	Routine	Aza, Pred, Tac	Nystatin, Valgan, TMP/S	Pseudomonas	
Tx22	22	M	RA-ILD	P/N	B	5	0	54	Clinical	Cellcept, Pred, Tac	Nystatin, Valgan, TMP/S	Streptococcus pneumoniae	
Tx23	23	F	ILD	N/N	B	6	1	43	Routine	Cellcept, Pred, Tac	Nystatin, Valcyc, TMP/S	Streptococcus constellatus	
Tx27	27	M	ILD	P/N	B	10	0	57	Routine / Clinical	Cellcept, Pred, Tac	TMP/S		
Tx29	29	M	CF	N.D.	B	132	N.D.	46	Clinical	Cellcept, Pred, Tac	Vori, TMP/S, Azithro, Vanco, Meropenem	Escherichia coli, Pseudomonas aeruginosa	
Tx34	34A	M	A1AT	N/P	B	2	2	51	Routine	Cellcept, Pred, Tac	TMP/S, Valgan, Vori, Vanco	Mycobacterium avium-intracellulare	
Tx41	41A	M	Sarc, PHTN	P/P	B-H	1	N.D.	52	Routine	Cellcept, Pred, Tac	Nystatin, Valgan, TMP/S	S. aureus	C. albicans

Definition of abbreviations: A1AT = alpha 1-antitrypsin deficiency, Aza = azathioprine, Azithro = azithromycin; B = bilateral lung transplant, COPD = chronic obstructive pulmonary disease, CF = cystic fibrosis, B-H = bilateral lung transplant and heart transplant, ILD = interstitial lung disease associated with collagen vascular disease, IPF = idiopathic pulmonary fibrosis, Nystatin = nystatin oral swish, PCD = primary cilliary dyskinesia, PHTN = pulmonary hypertension, Pred = prednisone, RA-ILD = rheumatoid arthritis-associated interstitial lung disease, Sarc = sarcoid, S-L = single left lung transplant, S-R = single right lung transplant, Tac = tacrolimus, TMP/S = trimethoprim/sulfamethoxazole, Valcyc = valcyclovir, Valgan = valgancyclovir, Vori = voriconazole

* The consent for subjects Tx1 and Tx2 does not allow for the release of clinical data.
