# Supplementary material for: Improved characterization of medically relevant fungi in the human respiratory tract using next-generation sequencing
Source: Genome Biol. 2014 Oct 25;15(10):487. doi: 10.1186/s13059-014-0487-y (PMC4232682; doi:10.1186/s13059-014-0487-y)
Supplement: Additional file 2: — Analysis of fungal communities. [file 13059_2014_487_MOESM2_ESM.pdf]

Supplemental report: Anaysis of fungal communities

Contents

1 Fungal diversity and community composition **2**

1.1 Number of subjects and study groups . . . 2

1.2 Number of samples included . . . 2

1.3 Number of reads, OTUs, and fungal genera detected . . . 2

1.4 α-diversity of ITS samples . . . 3

1.5 β-diversity of ITS samples . . . 3

1.6 Reproducibility of ITS results in repeat extractions . . . 3

2 Analysis of PicoGreen-corrected OTU abundances **12**

2.1 Genus-specific OTU abundances . . . 12

3 Identifying fungi in oropharyngeal and BAL samples that were unlikely to originate from contamination **13**

3.1 Fungi unlikely to arise from contamination sources, extended plots . . . 13

1 Fungal diversity and community composition

1.1 Number of subjects and study groups

Table 1 shows the subjects in each study group. As described in the main text and Additional file 1, subjects 3D03-3D06 were also sampled in group 3B more than a year previous to the 3D sample, using a two-scope rather than a one-scope bronchoscopy procedure.

Group	N	Subjects
1A	8	1A01, 1A02, 1A03, 1A05, 1A06, 1A07, 1A09, 1A10
1B	3	1B02, 1B03, 1B07
2B	8	2B01, 2B02, 2B05, 2B06, 2B07, 2B09, 2B10, 2B12
3B	6	3B06, 3B07, 3B08, 3B09, 3B10, 3B11
3C	4	3C04, 3C05, 3C06, 3C07
3D	6	3D01, 3D02, 3D03, 3D04, 3D05, 3D06
Pulm	13	Pulm1, Pulm2, Pulm3, Pulm4, Pulm5, Pulm6, Pulm7, Pulm8, Pulm9, Pulm10, Pulm11, Pulm12, Pulm13
Transplant	42	Tx1, Tx2, Tx3, Tx4, Tx5, Tx6, Tx7, Tx8, Tx10, Tx11, Tx12, Tx14, Tx16, Tx17, Tx18, Tx19, Tx21, Tx22, Tx23, Tx24, Tx25, Tx26, Tx27, Tx29, Tx30, Tx31, Tx32, Tx33, Tx34, Tx35, Tx36, Tx38, Tx39, Tx40, Tx41, Tx42, Tx43, Tx44, Tx45, Tx46, Tx47, Tx49

Table 1: Subjects appearing in this study.

1.2 Number of samples included

Table 2 shows the number of each sample type appearing in the study groups. Table 3 shows the number of contamination control samples not associated with a particular subject. Table 4 shows the aggregate number of contamination control, OW, and BAL samples, counting across all study groups.

	1A	1B	2B	3B	3C	3D	Pulm	Transplant
Scope 1 pre-wash	2	3	0	0	–	5	6	46
Scope 2 pre-wash	8	3	7	6	–	–	–	–
Oral wash	8	3	8	6	4	6	11	23
Scope 1 post-wash	2	3	0	0	–	–	–	–
BAL (single sample)	–	–	–	–	–	–	13	47
BAL A 1st return	8	3	8	6	–	6	–	–
BAL A 2nd return	8	2	8	6	–	6	–	–
BAL B	7	2	7	6	–	6	–	–

Table 2: Sample types included for each study group.

1.3 Number of reads, OTUs, and fungal genera detected

Over 355 samples in the main study, we collected a median value of 798 reads per sample. A total of 1801 OTUs were formed, representing 277 fungal genera. Table 5 summarizes the taxonomic assignments for non-fungal OTUs. Table 6 breaks down the taxonomic assignments, showing the percentage of fungal OTUs assigned at each rank from phylum to genus.

To summarize the fungal porportions in the main text, we plot the OW and BAL samples for all non-transplant subjects. Where multiple BAL samples are available, we show the BAL A 2nd

	Sample type
Sterile swab	8
Room	8
PCR water	4
Water	17
Saline	9

Table 3: Number of contamination control sample types.

	Number of samples
OW	69
BAL	149
Contamination control	132

Table 4: Aggregate number of BAL, OW, and contamination control samples.

return. To represent the contamination control samples, we show all contamination control samples excluding those collected from bronchoscope pre-washes (included samples are listed in Table 3). Genera represented by more than 100 reads in this sample set are shown in the heatmap.

To supplement the fungal proportions shown in the main text, we produce a set of figures here showing the proportions in each study group. Figures 1–8 show the proportions fungi in each sample of the study, summarized at the genus level. In each table, genera shown are represented by more than 100 reads.

1.4 α-diversity of ITS samples

Table 7 lists the median number of fungal species observed per sample in each body site, at a sampling depth of 300 reads per sample.

The Shannon diversity of fungal communities is plotted for each sample in Figure 9. Above a value of 100 reads/sample, the value is approximately constant.

1.5 β-diversity of ITS samples

To compare the composition of fungi in BAL, OW, and contamination control samples, we computed the Jaccard and Bray-Curtis distance between each pair of samples. Jaccard distance measures the percentage of OTUs in common between two samples, while Bray-Curtis distance measures the normalized difference in OTU abundances between samples.

Figure 10 shows the Jaccard distances between samples, ordinated by principal coordinates analysis (PCoA). A PERMANOVA test for difference in group centroid resulted in a significant difference for OW samples vs. contamination controls (Table 8), but no significant difference in centroid for BAL vs. contamination controls (Table 9).

A PCoA plot of Bray-Curtis distance is shown in Figure 11. A PERMANOVA test of Bray-Curtis distance yielded a significant difference between OW and contamination control samples (Table 10) but not between BAL and contamination control samples (Table 11).

1.6 Reproducibility of ITS results in repeat extractions

To investigate the reproducibility of ITS sequencing results across repeat extractions from the same source material, we re-extracted, re-amplified, and re-sequenced material from 18 samples. The sample types, listed in Table 12, include bronchoscope pre-wash, oropharyngeal wash, and BAL.

The proportions of fungal genera recovered in repeat extractions are shown in Figure 12. The abundance of each genus is shown in Figure 13 after conversion to PicoGreen-corrected abundance.

For each set of replicate samples, we asked if OTUs appearing with more than 50% proportion in one replicate were present across all replicate samples. The results of this analysis are listed in

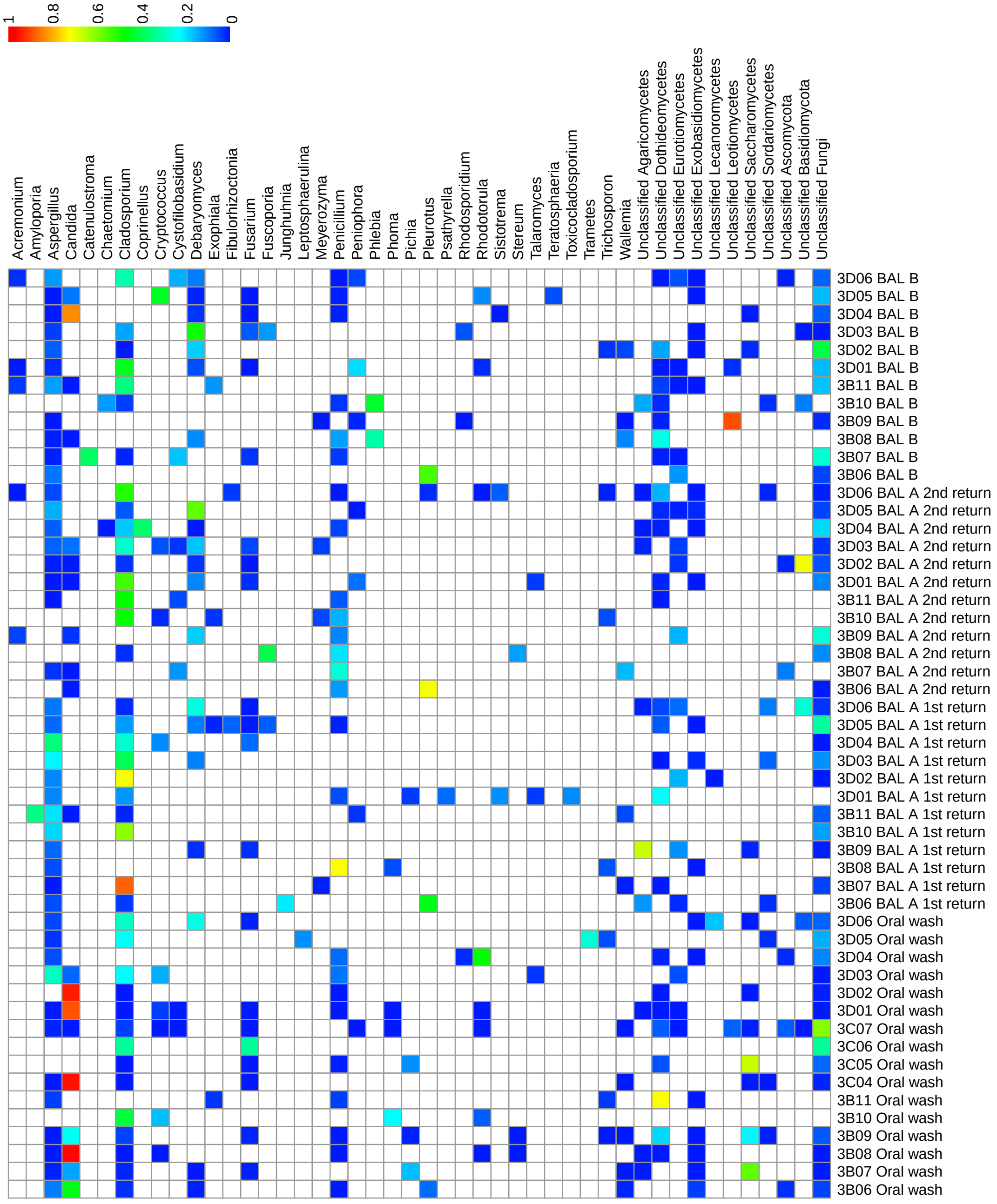

Figure 1: Observed proportions of fungal genera in healthy subjects.

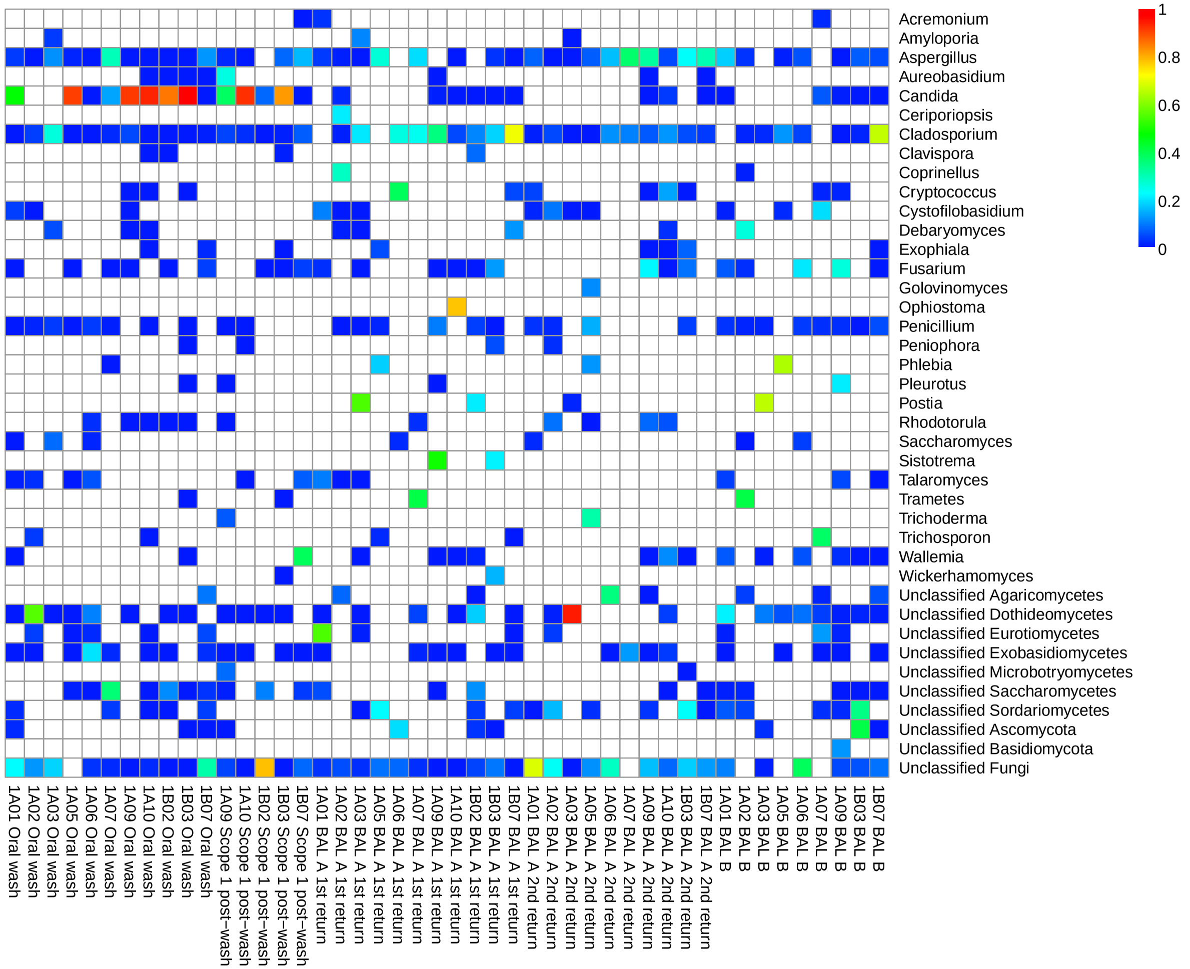

Figure 2: Observed proportions of fungal genera in HIV+ subjects not on ART.

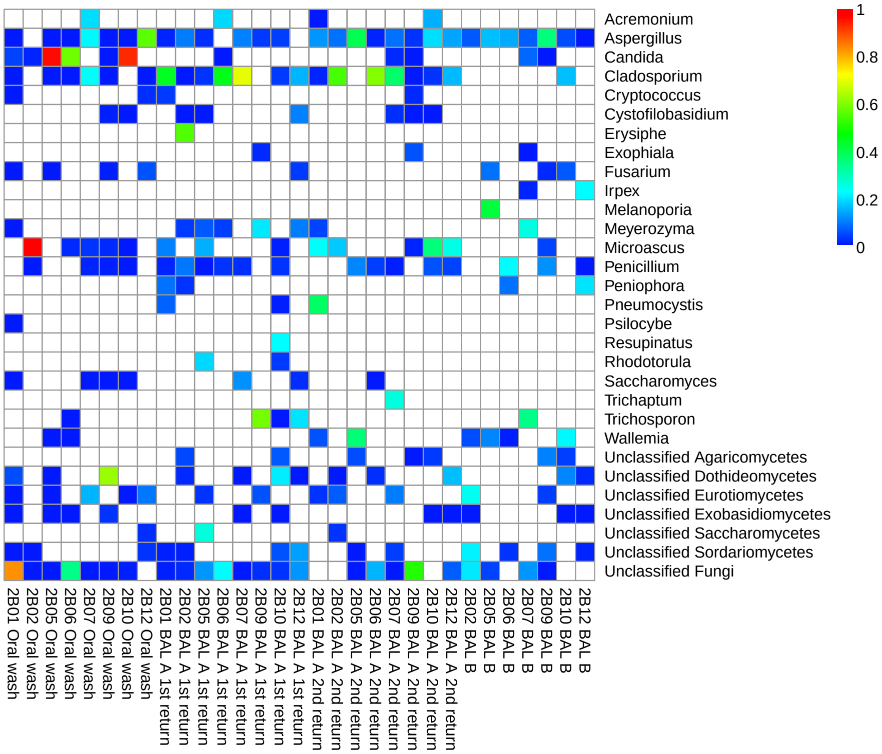

Figure 3: Observed proportions of fungal genera in HIV+ subjects currently on ART.

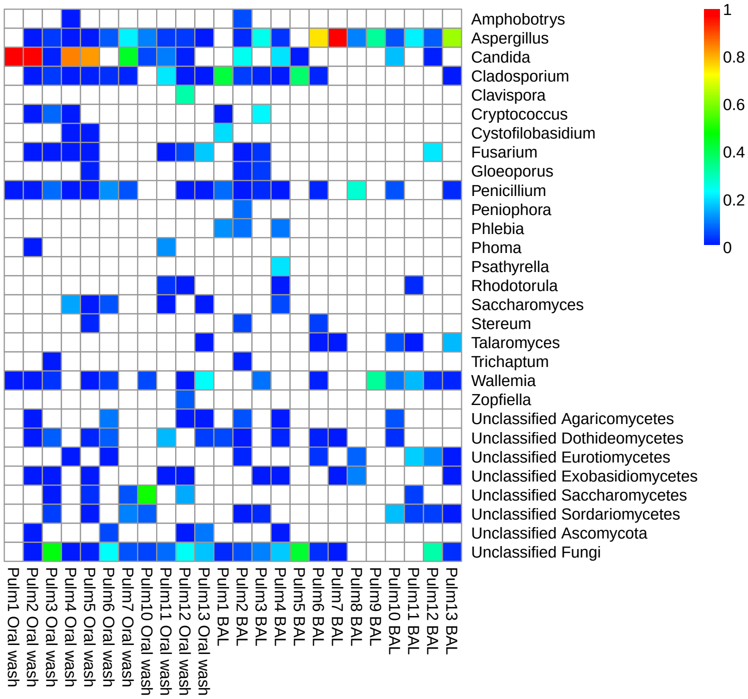

Figure 4: Observed proportions of fungal genera in subjects undergoing bronchoscopy for a variety of purposes.

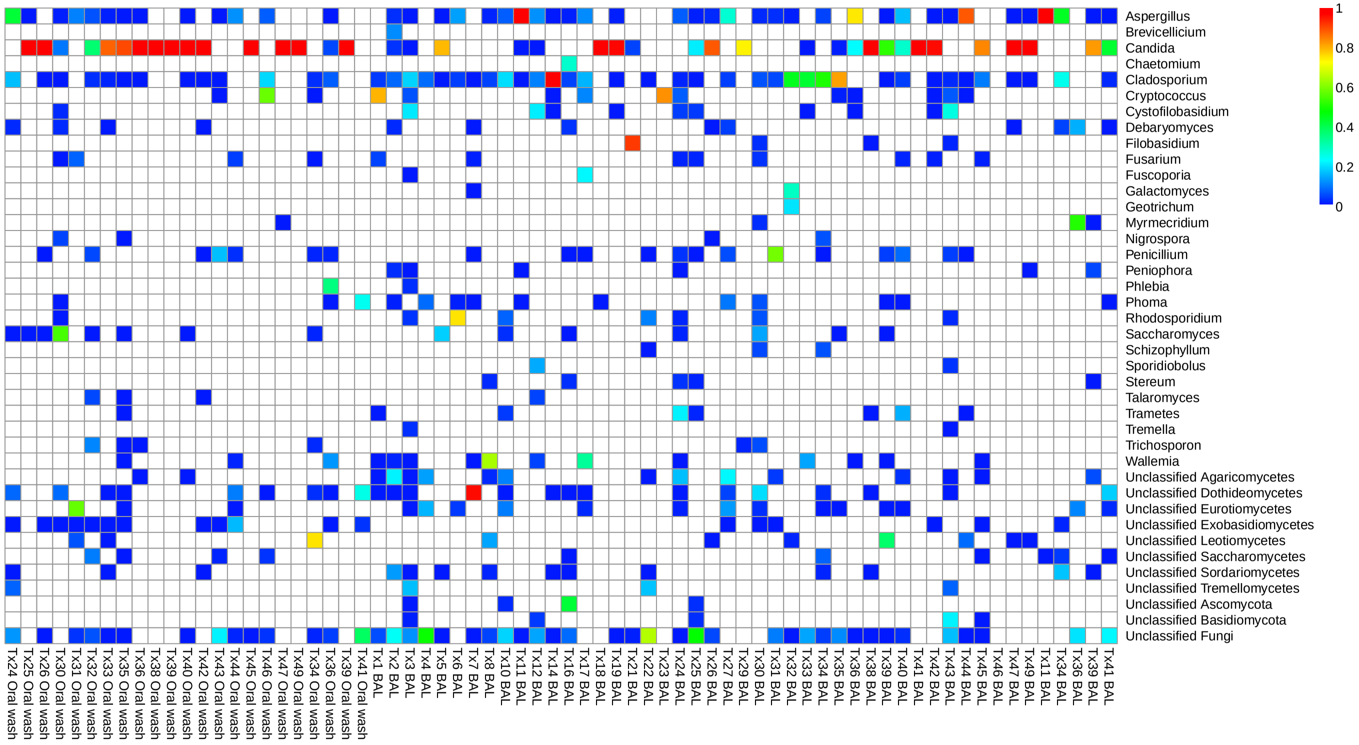

Figure 5: Observed proportions of fungal genera in lung transplant recipients.

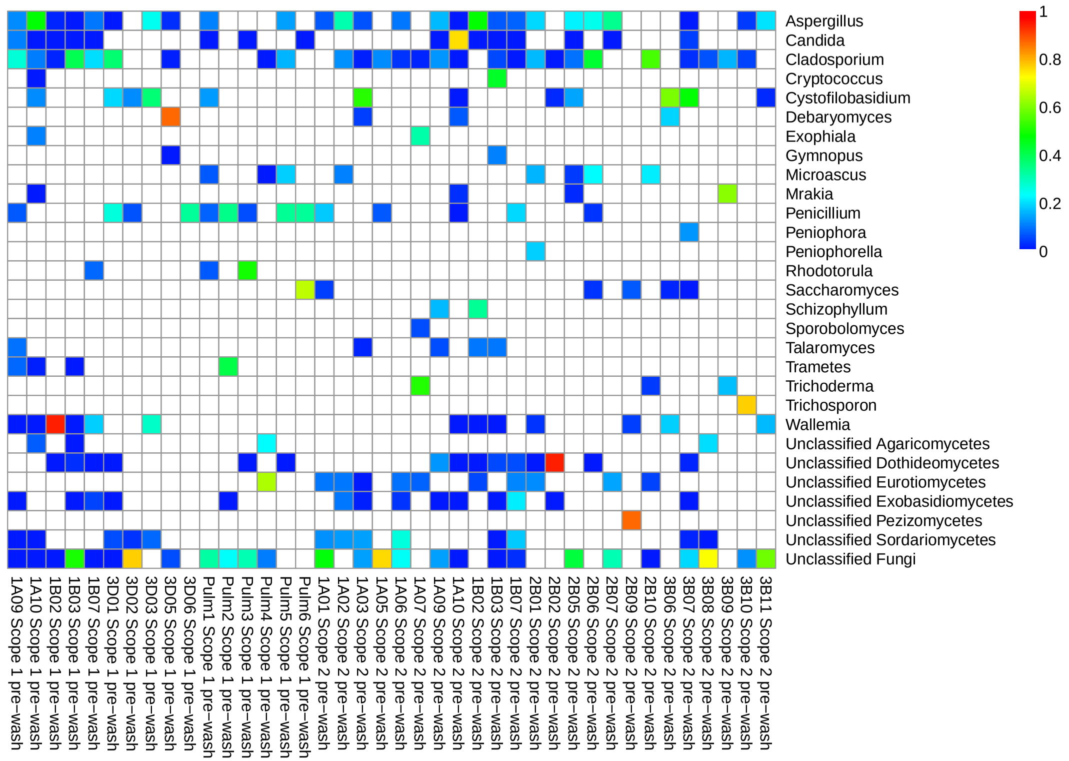

Figure 6: Observed proportions of fungal genera in contamination control samples from bronchoscope pre-wash (non-transplant subjects).

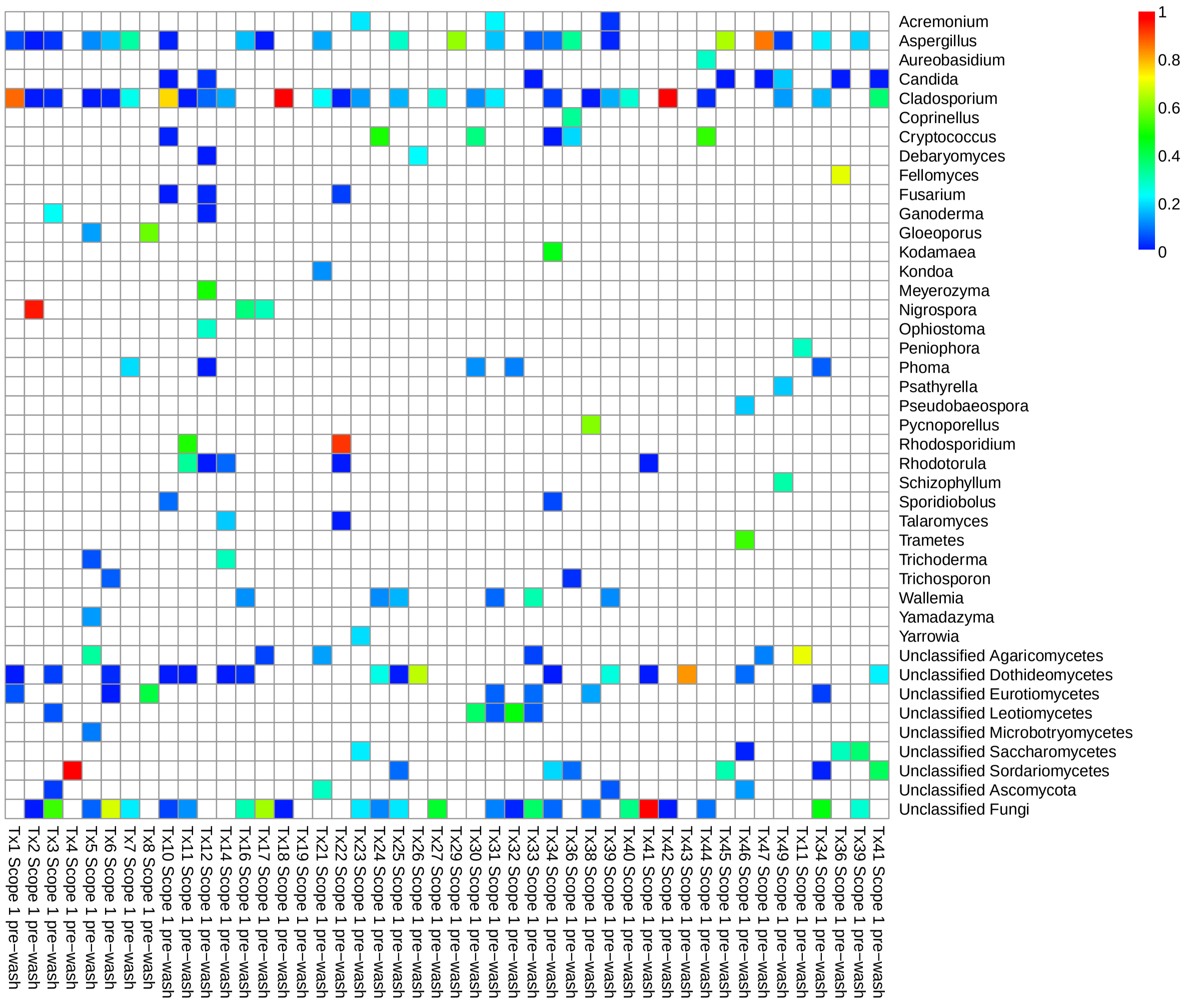

Figure 7: Observed proportions of fungal genera in contamination control samples from bronchoscope pre-wash (transplant subjects).

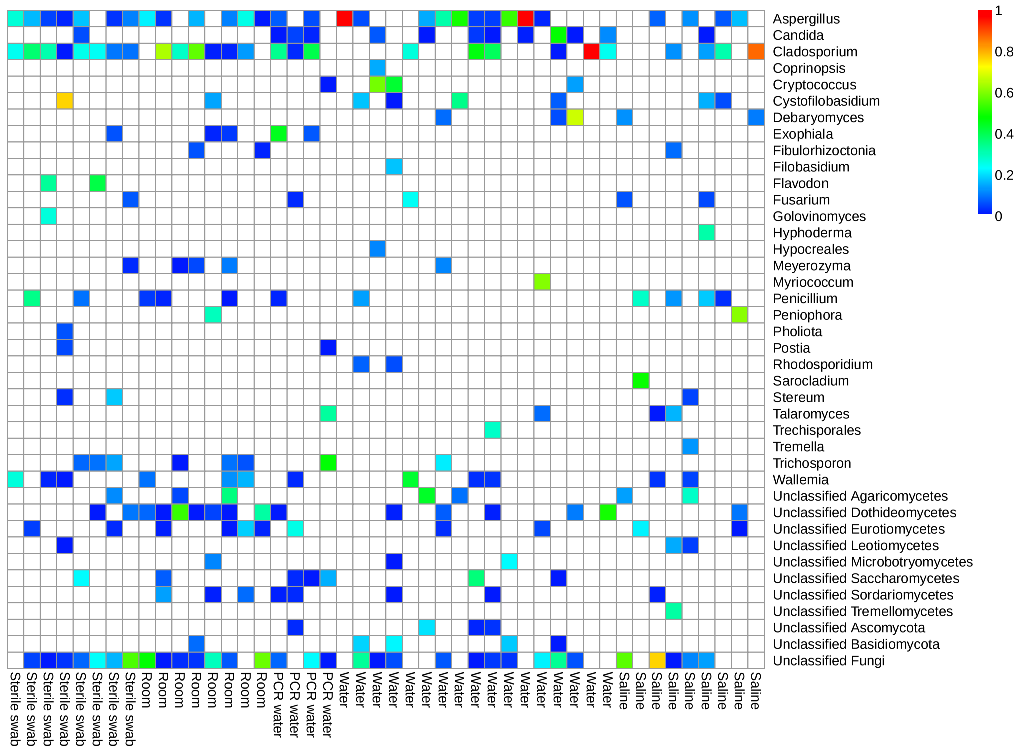

Figure 8: Observed proportions of fungal genera in contamination control samples from environmental sources.

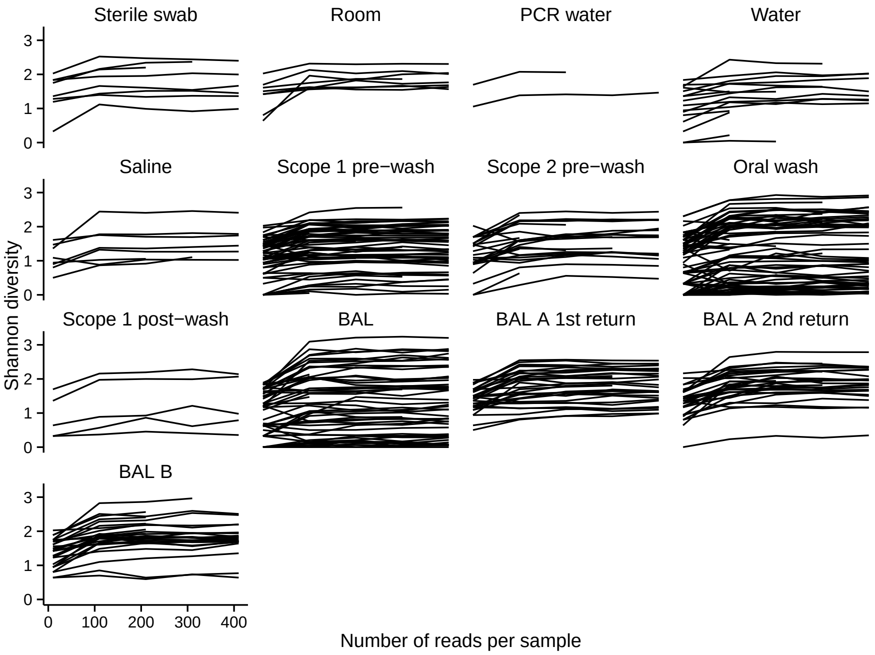

Figure 9: Shannon diversity of fungal communities as a function of sequencing depth.

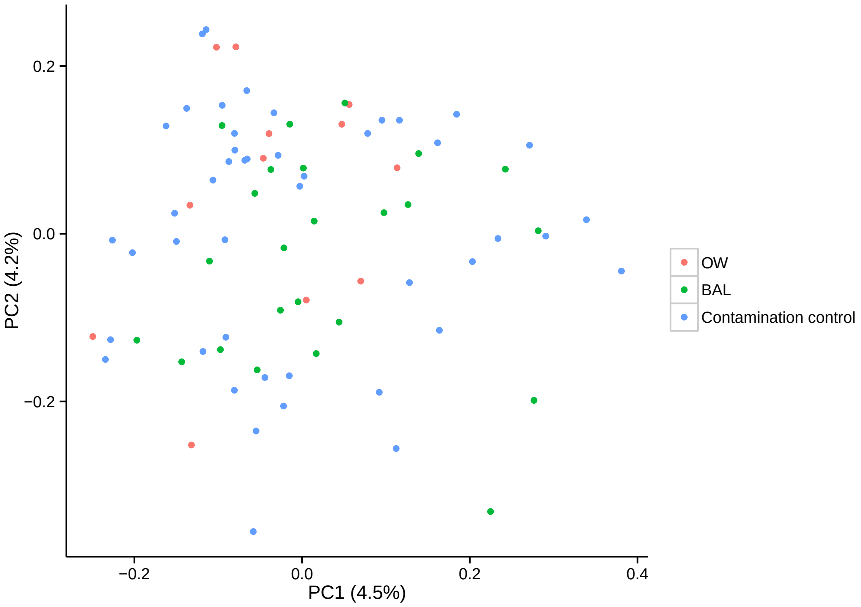

Figure 10: Principal coordinate analysis of Jaccard distances between samples.

	Number of OTUs
No BLAST hits found in NCBI nt database	15
All hits were filtered for low quality	29
Abundance of low coverage hits (possible chimera)	77
No consensus at domain level (no classification)	1
Bacteria	20
Unclassified Eukaryota	9
Human	53
Termite	1
Viridiplantae	8
Fungi	1588

Table 5: Summary of non-fungal OTU assignments.

	Percentage of fungal OTUs
Phylum	90.49
Class	83.07
Order	79.33
Family	74.80
Genus	65.12
Species	36.01

Table 6: Percentage of fungal OTUs classified at each taxonomic rank.

Table 13.

We followed this analysis by modeling the probability of obseriving OTUs across repeat extractions as a function of OTU proportion or PicoGreen-corrected abundance. Figure 14 shows the best fit for each predictor, using a Generalized Additive Model with cubic spline smoothing. The model parameters are listed in Table 14 and Table 15. The PicoGreen-corrected abundance model provides a better fit to the observed data.

To provide further insight on the ability of OTU proportion or PicoGreen-corrected abundance to predict the appearance of OTUs across replicate extractions, we generated an ROC curve for each predictor. The curve is plotted in Figure 15. The area under the curve (AUC) is 0.71 for proportions and 0.78 for PicoGreen-corrected abundance, indicating that the latter quantity is a better predictior for observing OTUs in repeat fungal extractions.

Scatterplots of the OTU proportions for each pair of replicate samples are given in Figures 16-33.

2 Analysis of PicoGreen-corrected OTU abundances

2.1 Genus-specific OTU abundances

We next created genus-specific contamination thresholds for some of the more common genera observed in contamination control samples, to see if they would be compatible with the global threshold derived from all genera together. Figure 34 shows the nonzero PicoGreen-corrected abundances for genera appearing in at least 10 contamination control samples.

For each genus appearing in the figure, we estimated a 95% abundance threshold and computed confidence intervals using a bootstrap approach. The results are listed in Table 16. For every genus but one, the genus-specific contamination threshold was consistent with the global threshold. For Cladosporium, the genus-specific threshold was found to be about 3 times higher than the global threshold. Cladosporium was the most commonly occurring genus in contamination control samples, which allowed us to generate a relatively narrow confidence interval.

Figure 35 shows the level of agreement between ITS sequencing and clinical culture results, as evaluated by Cohen's Kappa. The value obtained, about 0.65, was not sensitive to the abundance

body_site	Median richness
OW	14.27
BAL	14.00
Contamination control	9.48

Table 7: Median number of species observed in OW, BAL, and contamination control samples.

	df	SS	MS	F	R2	P
Body site	1	0.61	0.61	1.33	0.02	0.006
Residuals	56	25.61	0.46		0.98	
Total	57	26.21			1.00	

Table 8: PERMANOVA test of Jaccard distance between fungal comminities in healthy subjects: OW vs. contamination control.

threshold selected.

3 Identifying fungi in oropharyngeal and BAL samples that were unlikely to originate from contamination

3.1 Fungi unlikely to arise from contamination sources, extended plots

Figures 36-39 show the PicoGreen-corrected abundance for OTUs exceeding the 95% contamination threshold, summarized by genus.

	df	SS	MS	F	R2	P
Body site	1	0.50	0.50	1.10	0.02	0.172
Residuals	68	30.91	0.45		0.98	
Total	69	31.41			1.00	

Table 9: PERMANOVA test of Jaccard distance between fungal comminities in healthy subjects: BAL vs. contamination control.

	df	SS	MS	F	R2	P
Body site	1	1.00	1.00	2.22	0.04	0.001
Residuals	56	25.39	0.45		0.96	
Total	57	26.39			1.00	

Table 10: PERMANOVA test of Bray-Curtis distance between fungal comminities in healthy subjects: OW vs. contamination control.

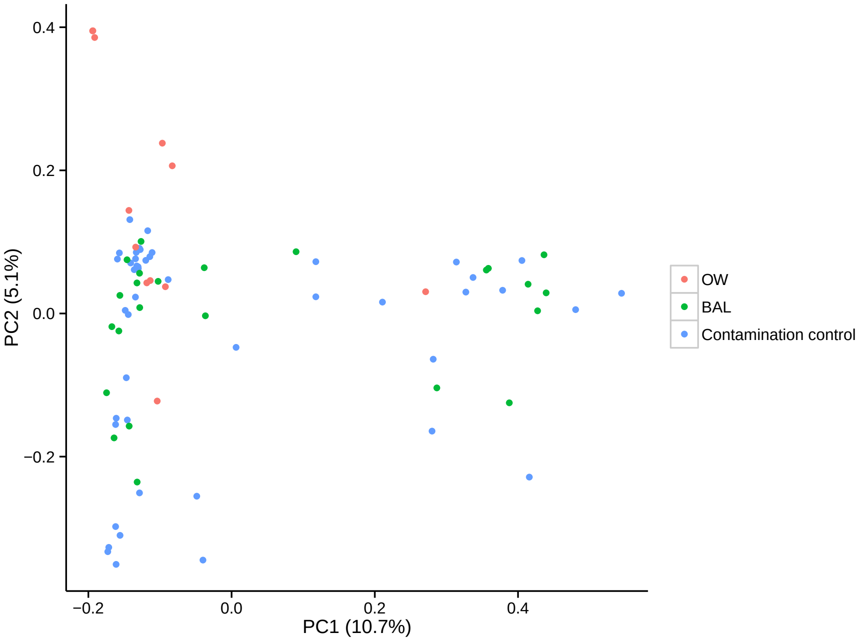

Figure 11: Principal coordinate analysis of Bray-Curtis distances between samples.

	df	SS	MS	F	R2	P
Body site	1	0.59	0.59	1.31	0.02	0.077
Residuals	68	30.57	0.45		0.98	
Total	69	31.16			1.00	

Table 11: PERMANOVA test of Bray-Curtis distance between fungal comminities in healthy subjects: BAL vs. contamination control.

	Number of replicates
Tx44 Scope 1 pre-wash	2
Tx45 Scope 1 pre-wash	2
Tx36 Scope 1 pre-wash	2
Tx39 Scope 1 pre-wash	2
Pulm5 Scope 1 pre-wash	2
Pulm6 Scope 1 pre-wash	2
Tx44 Oral wash	4
Tx45 Oral wash	4
Tx36 Oral wash	4
Tx39 Oral wash	4
Pulm5 Oral wash	4
Pulm6 Oral wash	4
Tx44 BAL	4
Tx45 BAL	4
Tx36 BAL	4
Tx39 BAL	4
Pulm5 BAL	3
Pulm6 BAL	4

Table 12: Number of repeat extractions for samples in reproducibility analysis.

Sample	Number of replicates	OTUs with >50% proportion detected in all samples
Tx44 Scope 1 pre-wash	2	FALSE
Tx45 Scope 1 pre-wash	2	FALSE
Tx36 Scope 1 pre-wash	2	FALSE
Pulm5 Scope 1 pre-wash	2	FALSE
Tx45 Oral wash	4	TRUE
Tx36 Oral wash	4	FALSE
Tx39 Oral wash	4	TRUE
Pulm5 Oral wash	4	TRUE
Pulm6 Oral wash	4	FALSE
Tx44 BAL	4	TRUE
Tx45 BAL	4	TRUE
Tx36 BAL	4	FALSE
Tx39 BAL	4	TRUE
Pulm5 BAL	3	FALSE
Pulm6 BAL	4	TRUE

Table 13: Repeat extraction samples where at least one OTU was detected with a proportion of 50%.

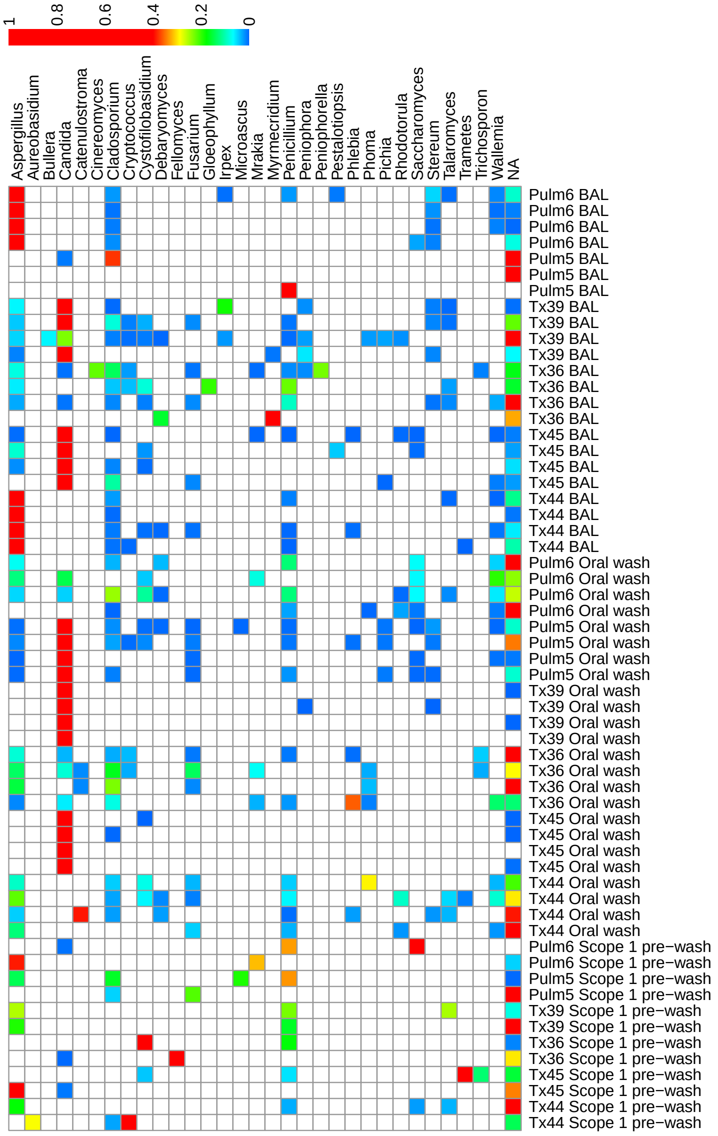

Figure 12: Heatmap of fungal proportions in repeat extractions.

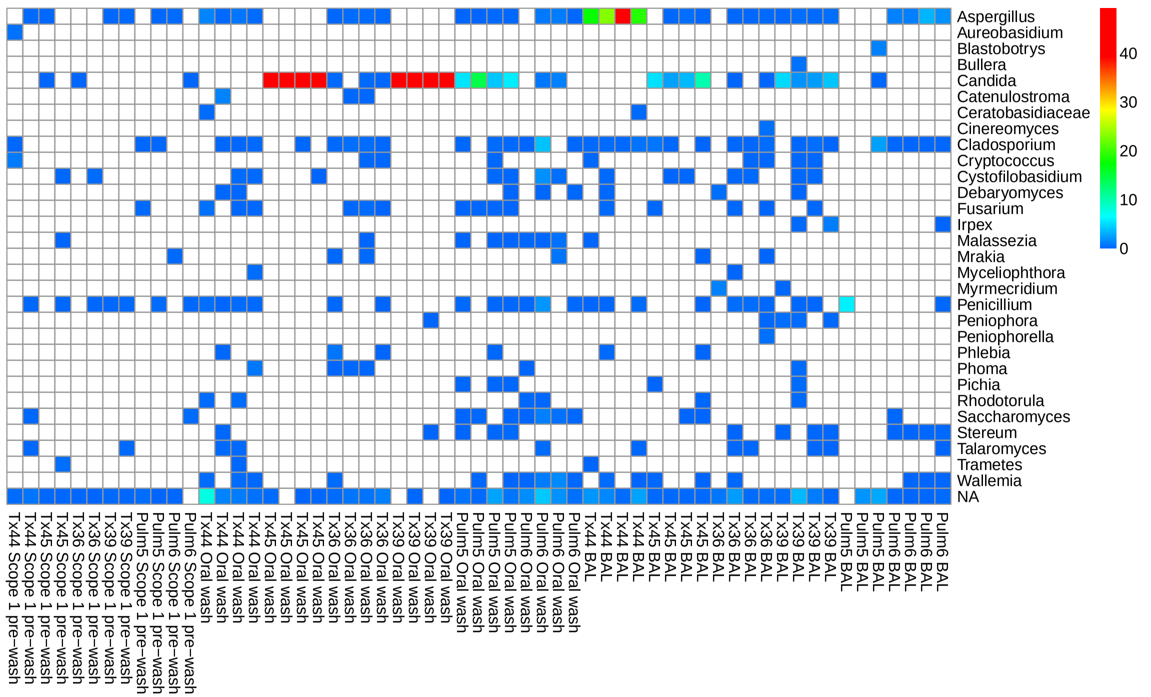

Figure 13: Heatmap of PicoGreen-corrected fungal abundance in repeat extractions.

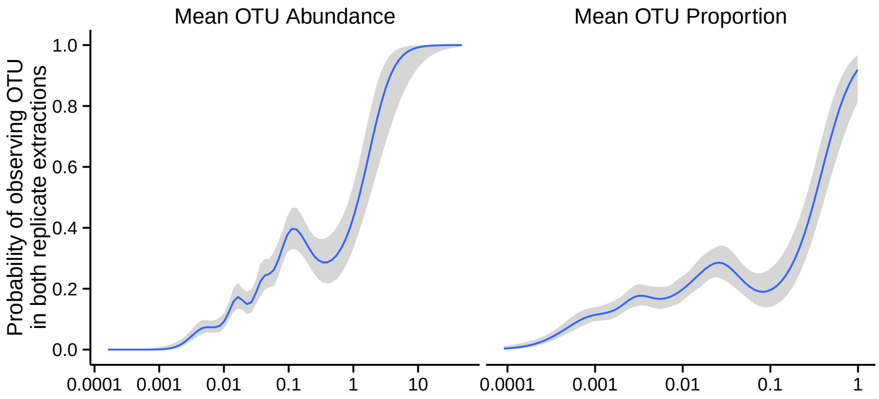

Figure 14: Probability of observing an OTU in repeat extractions as a function of OTU proportion or PicoGreen-corrected OTU abundance.

Family: binomial
Link function: logit
Parametric coefficients:

	Estimate	Std. Error	z value	Pr(>|z|)
(Intercept)	-2.08	0.08	-27.06	0.00

Approximate significance of smooth terms:

	edf	Ref.df	Chi.sq	p-value
s(log10(Mean_OTU_proportion))	7.36	8.22	192.46	0.00

Adjusted R-squared: 0.11
Deviance explained: 12.2

Table 14: Generalized additive model for observation of OTUs in repeat extractions, based on OTU proportion.

Family: binomial
Link function: logit
Parametric coefficients:

	Estimate	Std. Error	z value	Pr(>|z|)
(Intercept)	-2.44	0.16	-15.39	0.00

Approximate significance of smooth terms:

	edf	Ref.df	Chi.sq	p-value
s(log10(Mean_OTU_abundance))	8.55	8.93	250.69	0.00

Adjusted R-squared: 0.17
Deviance explained: 18.6

Table 15: Generalized additive model for observation of OTUs in repeat extractions, based on PicoGreen-corrected OTU abundance.

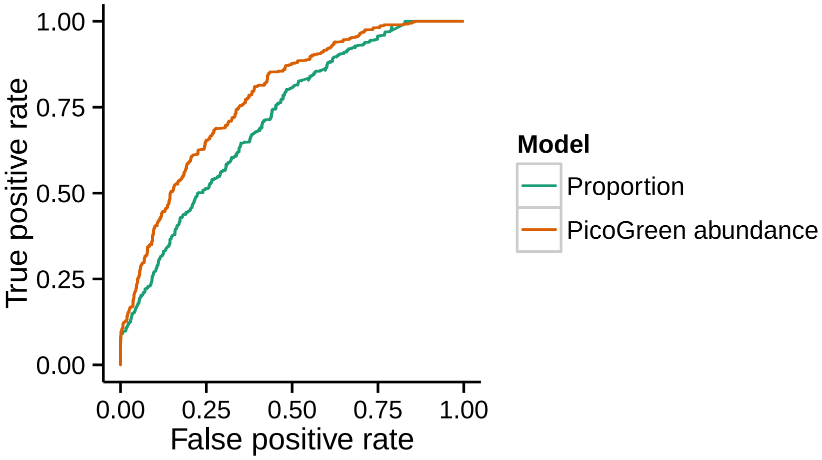

Figure 15: ROC curve for prediction of OTU occurrence in replicate sample pairs as modeled by OTU proportion or PicoGreen-corrected abundance.

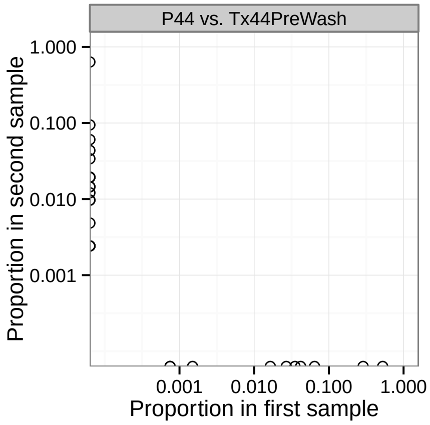

Figure 16: Proportion of fungal OTUs in repeat extractions of sample Tx44 Scope 1 pre-wash (log scale). OTUs absent in one sample are shown along the axis.

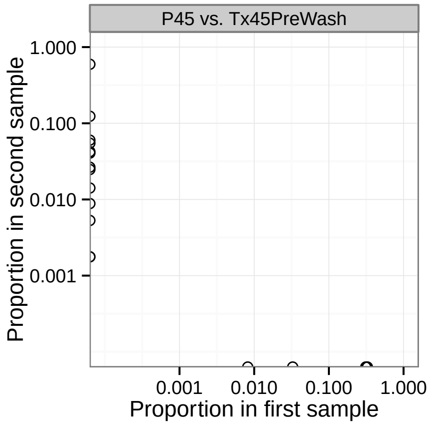

Figure 17: Proportion of fungal OTUs in repeat extractions of sample Tx45 Scope 1 pre-wash (log scale). OTUs absent in one sample are shown along the axis.

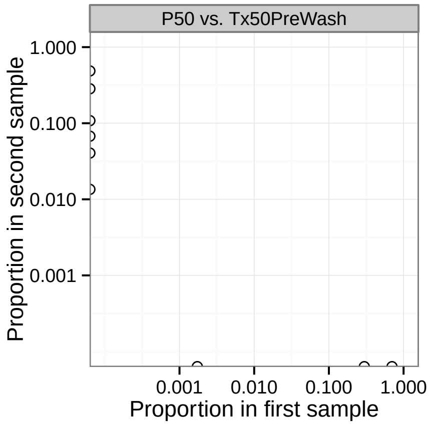

Figure 18: Proportion of fungal OTUs in repeat extractions of sample Tx36 Scope 1 pre-wash (log scale). OTUs absent in one sample are shown along the axis.

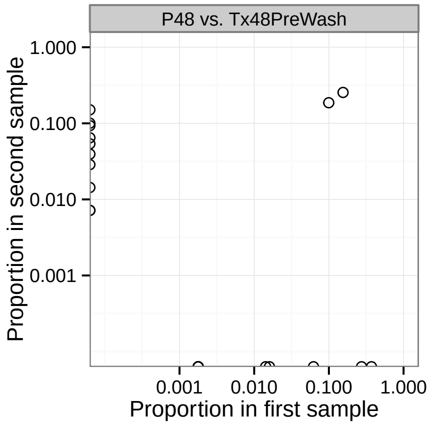

Figure 19: Proportion of fungal OTUs in repeat extractions of sample Tx39 Scope 1 pre-wash (log scale). OTUs absent in one sample are shown along the axis.

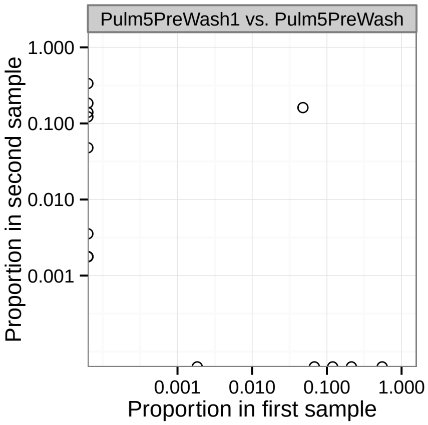

Figure 20: Proportion of fungal OTUs in repeat extractions of sample Pulm5 Scope 1 pre-wash (log scale). OTUs absent in one sample are shown along the axis.

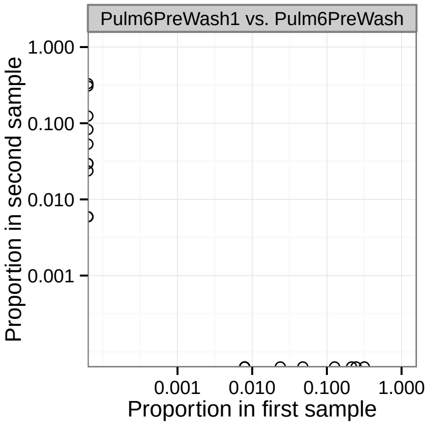

Figure 21: Proportion of fungal OTUs in repeat extractions of sample Pulm6 Scope 1 pre-wash (log scale). OTUs absent in one sample are shown along the axis.

Genus	Abundance threshold	95% CI, lower bound	upper bound
Aspergillus	0.329	0.221	0.399
Candida	0.355	0.044	3.268
Cladosporium	0.809	0.616	1.163
Cryptococcus	1.196	0.226	1.545
Cystofilobasidium	0.534	0.280	5.907
Debaryomyces	1.091	0.120	1.428
Fusarium	0.244	0.053	0.497
Penicillium	0.304	0.155	0.581
Saccharomyces	0.275	0.039	0.371
Talaromyces	0.093	0.064	0.109
Trichosporon	0.320	0.176	0.385
Wallemia	0.232	0.135	1.718

Table 16: Genus-specific abundance thresholds.

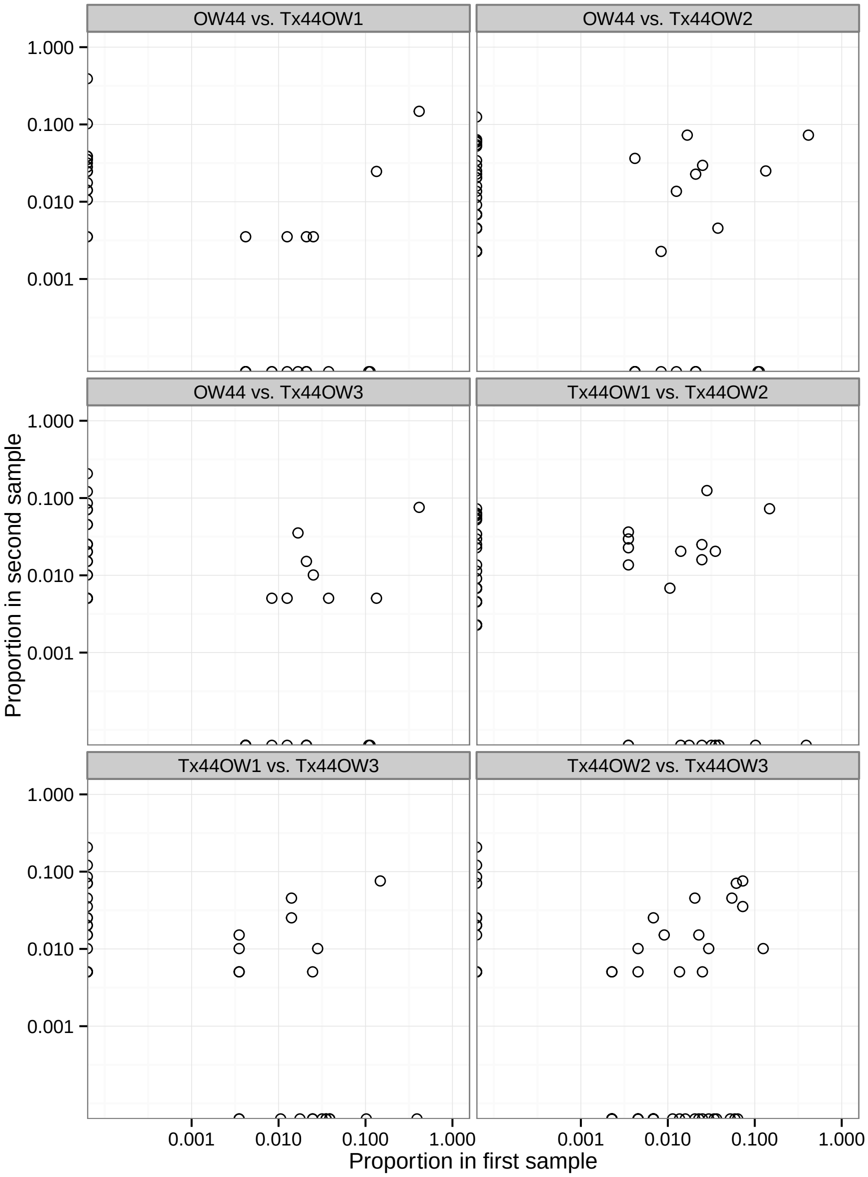

Figure 22: Proportion of fungal OTUs in repeat extractions of sample Tx44 Oral wash (log scale). OTUs absent in one sample are shown along the axis.

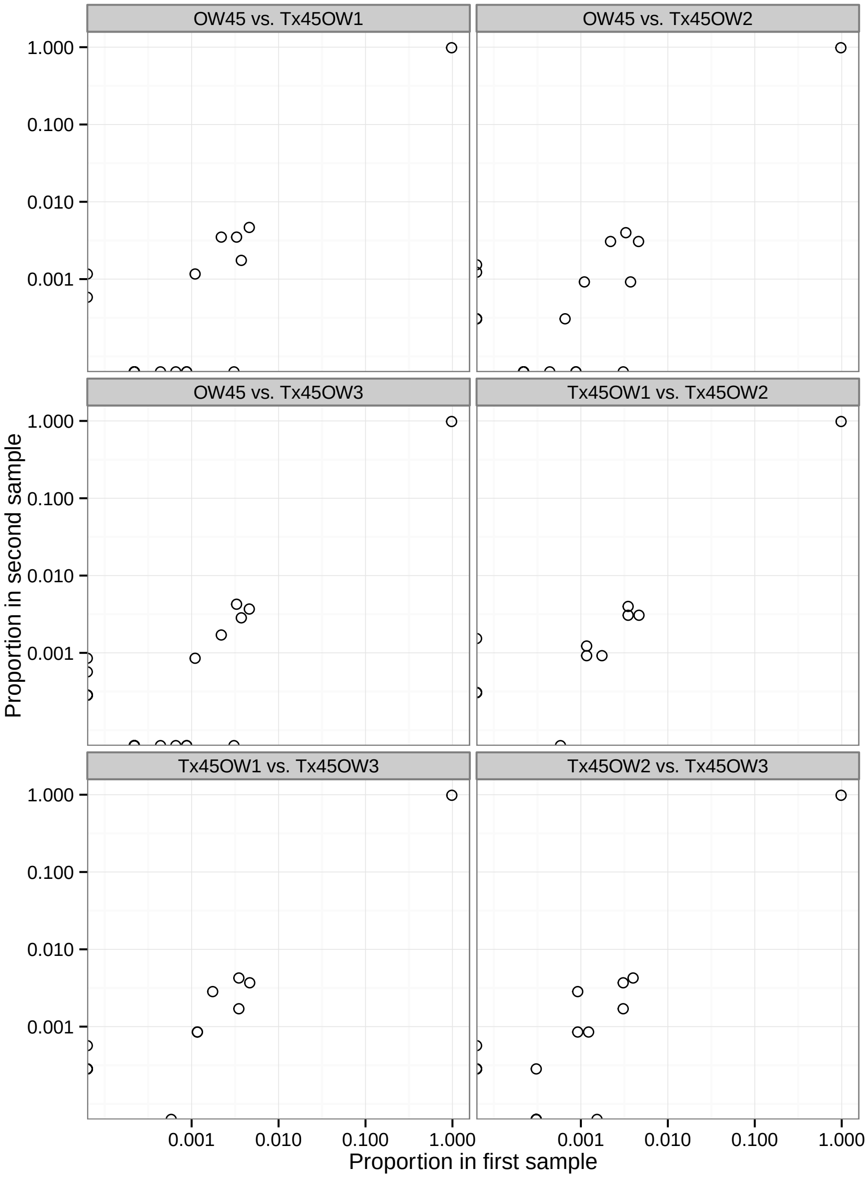

Figure 23: Proportion of fungal OTUs in repeat extractions of sample Tx45 Oral wash (log scale). OTUs absent in one sample are shown along the axis.

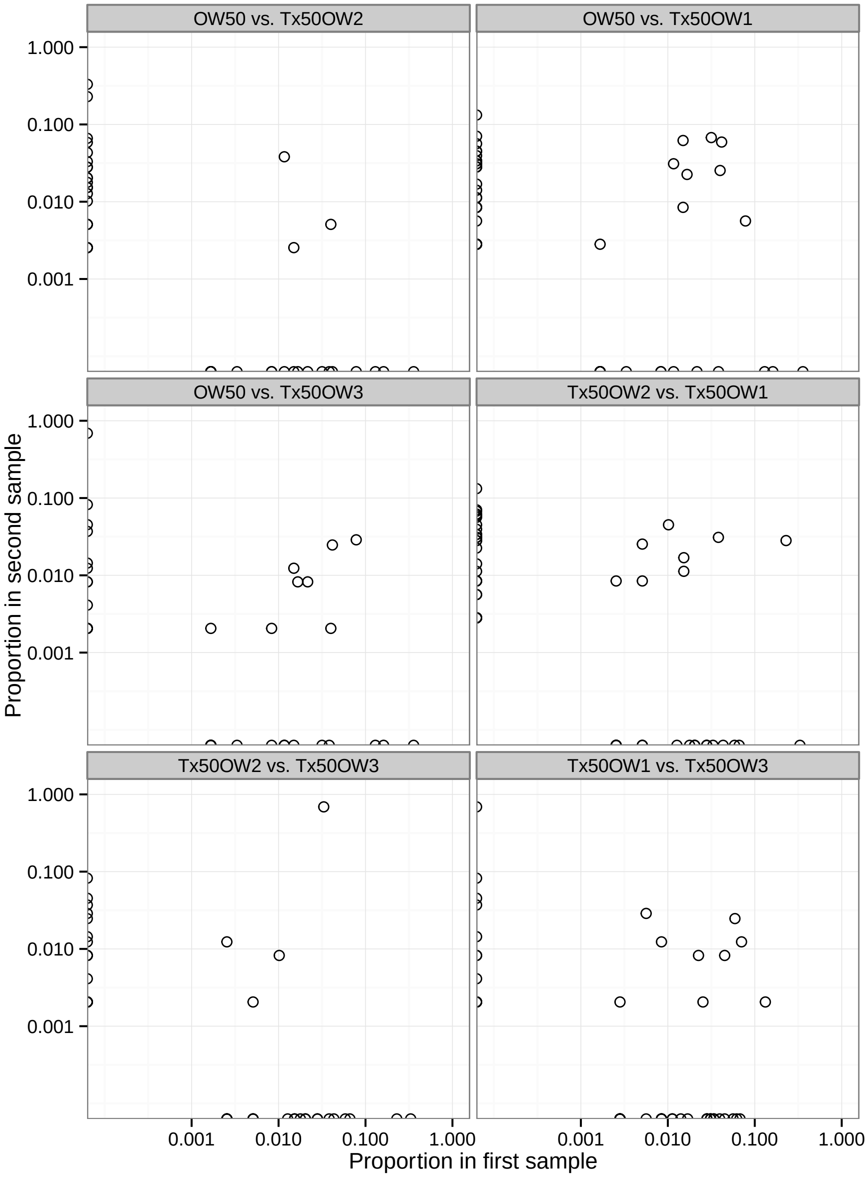

Figure 24: Proportion of fungal OTUs in repeat extractions of sample Tx36 Oral wash (log scale). OTUs absent in one sample are shown along the axis.

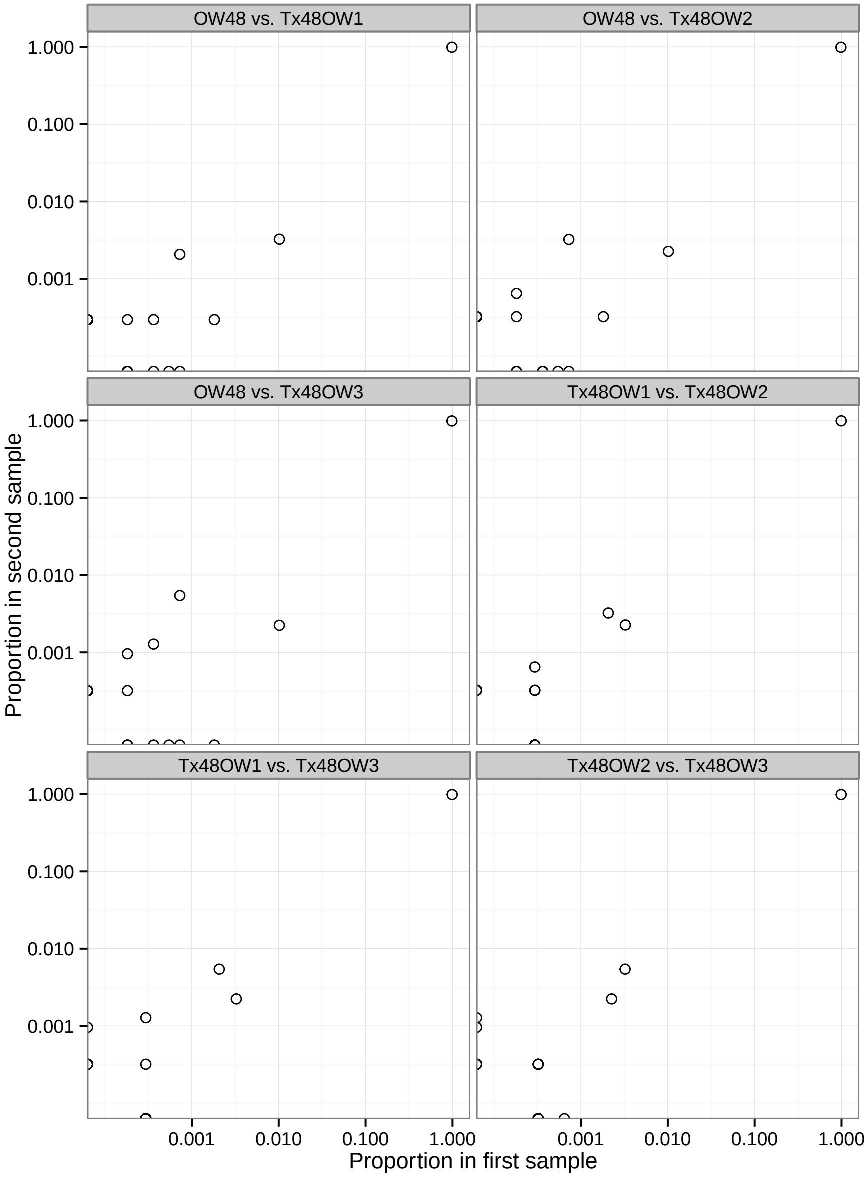

Figure 25: Proportion of fungal OTUs in repeat extractions of sample Tx39 Oral wash (log scale). OTUs absent in one sample are shown along the axis.

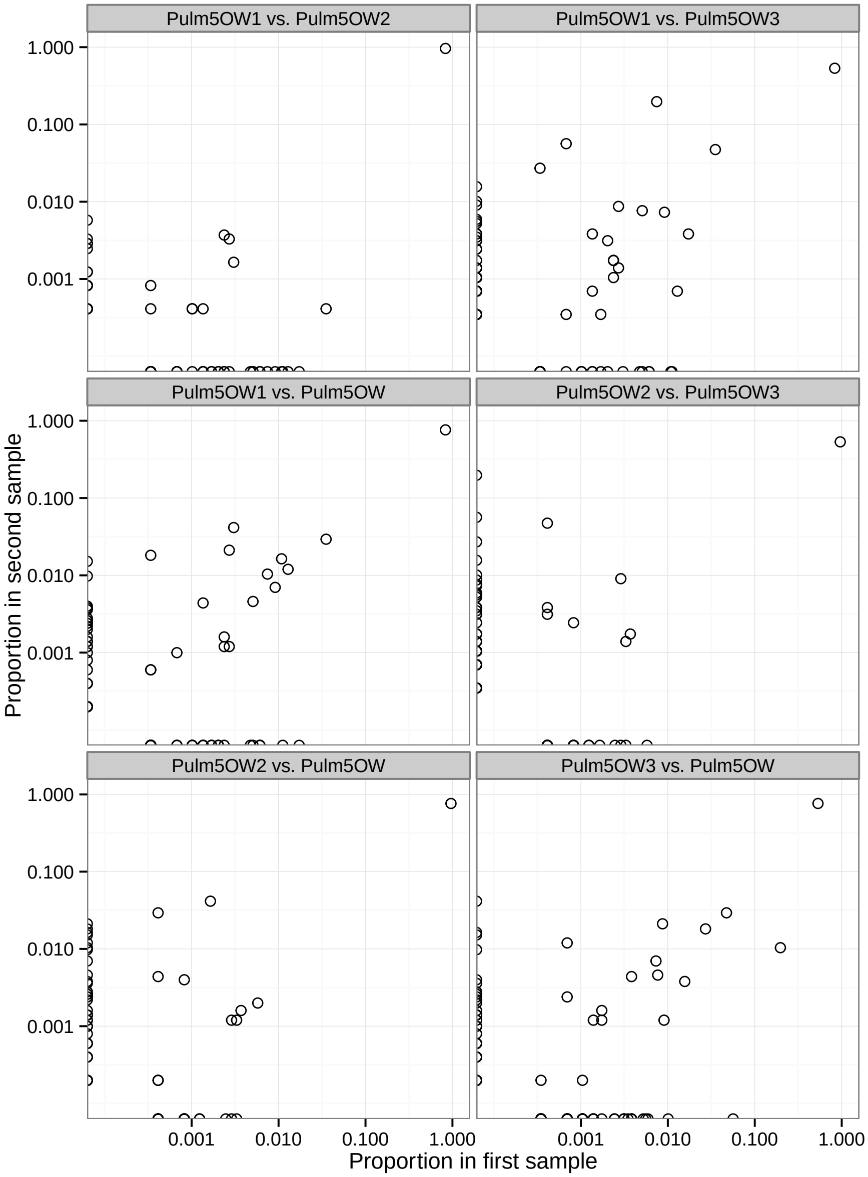

Figure 26: Proportion of fungal OTUs in repeat extractions of sample Pulm5 Oral wash (log scale). OTUs absent in one sample are shown along the axis.

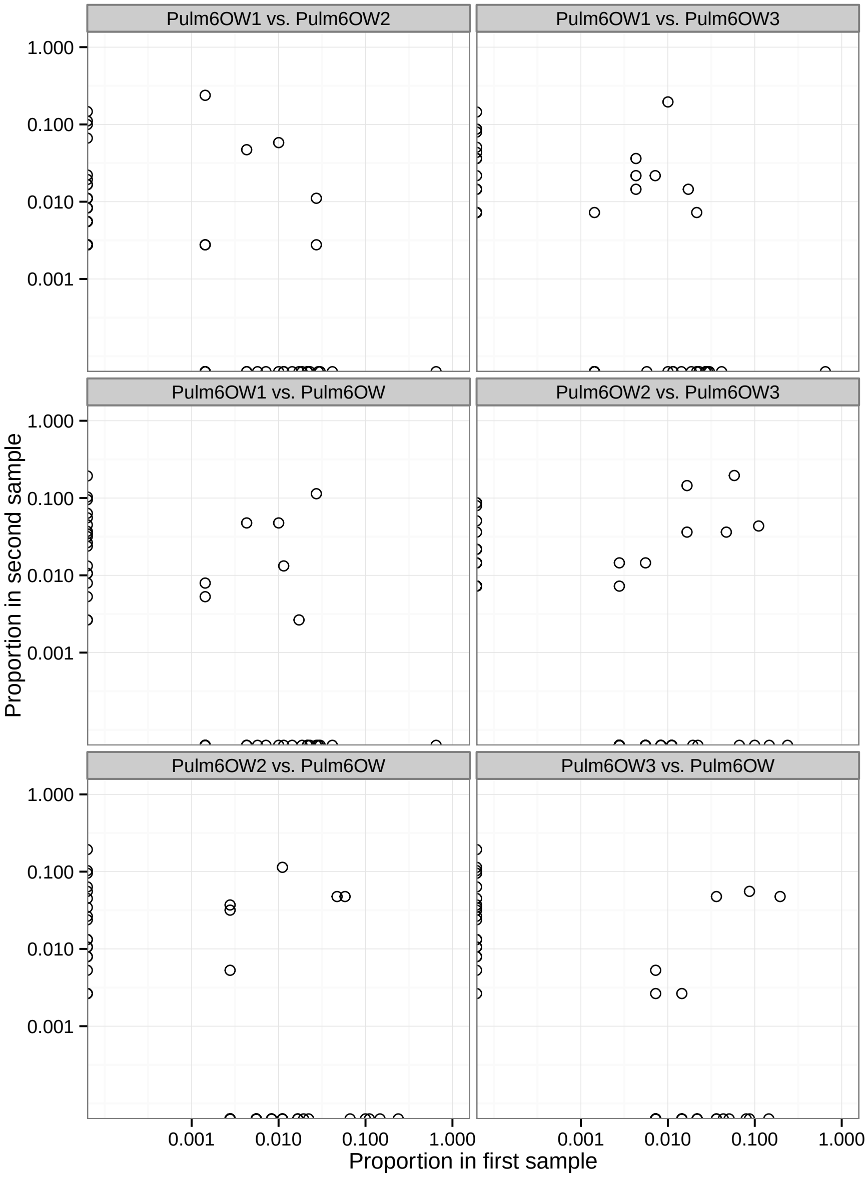

Figure 27: Proportion of fungal OTUs in repeat extractions of sample Pulm6 Oral wash (log scale). OTUs absent in one sample are shown along the axis.

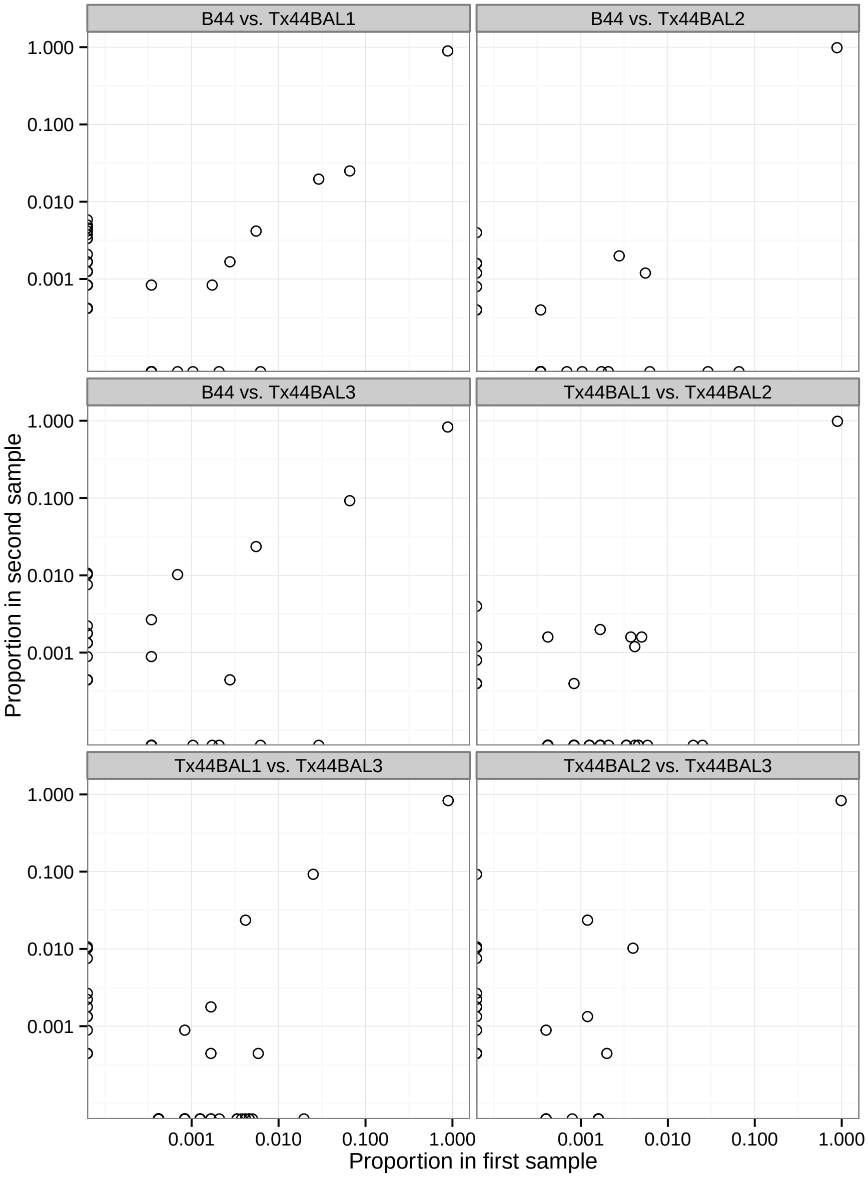

Figure 28: Proportion of fungal OTUs in repeat extractions of sample Tx44 BAL (log scale). OTUs absent in one sample are shown along the axis.

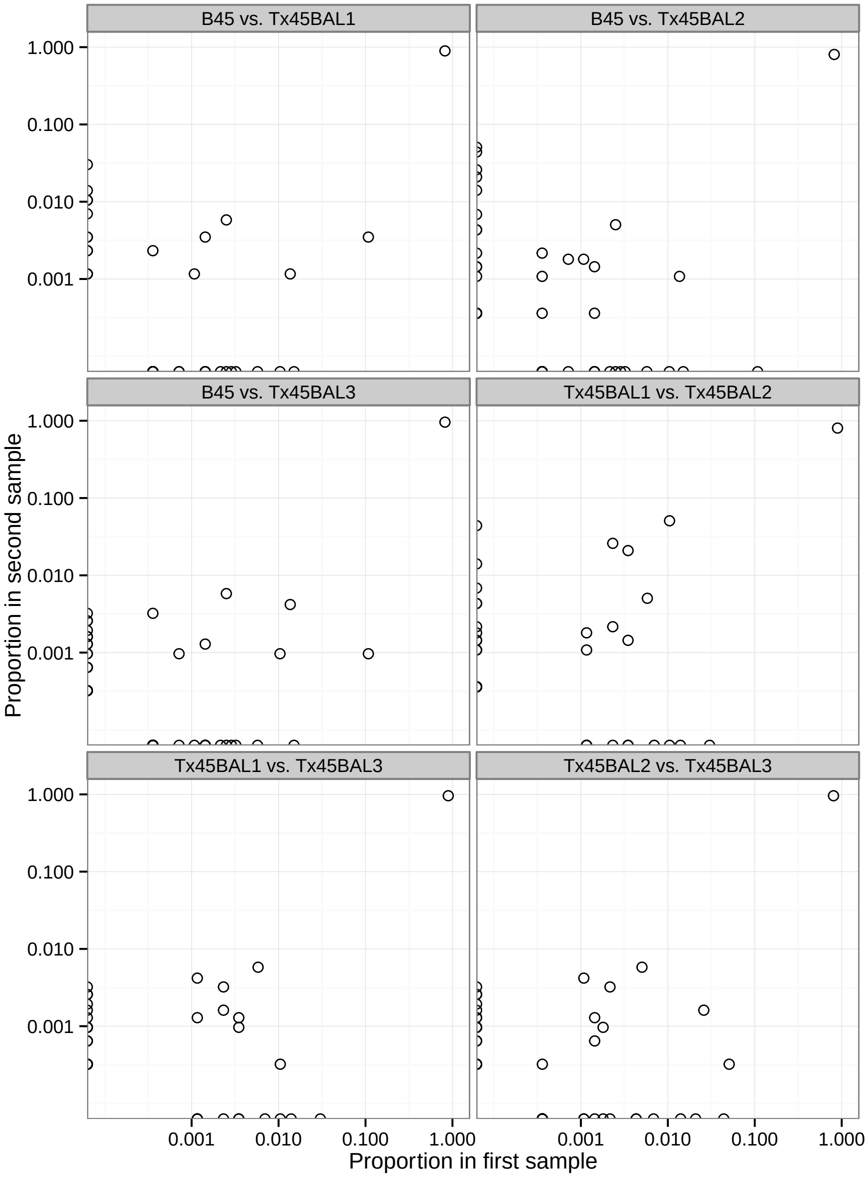

Figure 29: Proportion of fungal OTUs in repeat extractions of sample Tx45 BAL (log scale). OTUs absent in one sample are shown along the axis.

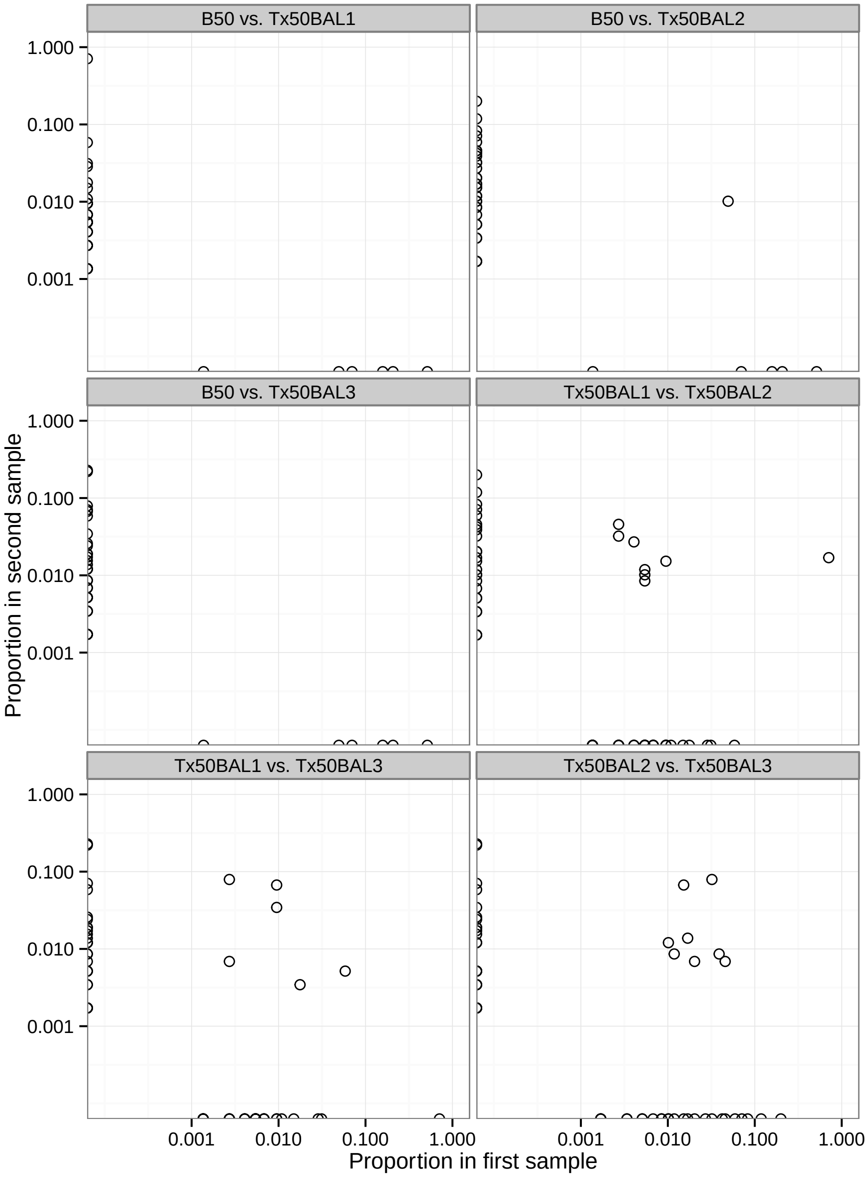

Figure 30: Proportion of fungal OTUs in repeat extractions of sample Tx36 BAL (log scale). OTUs absent in one sample are shown along the axis.

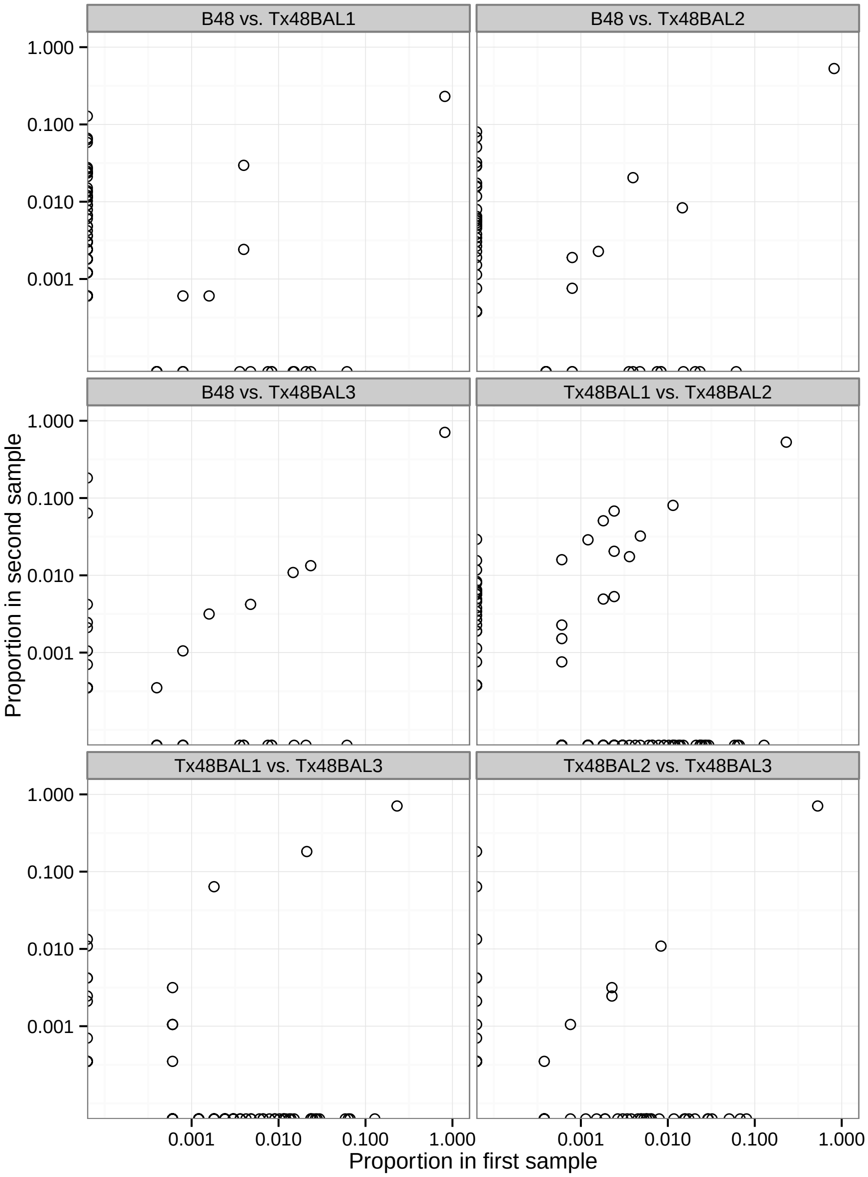

Figure 31: Proportion of fungal OTUs in repeat extractions of sample Tx39 BAL (log scale). OTUs absent in one sample are shown along the axis.

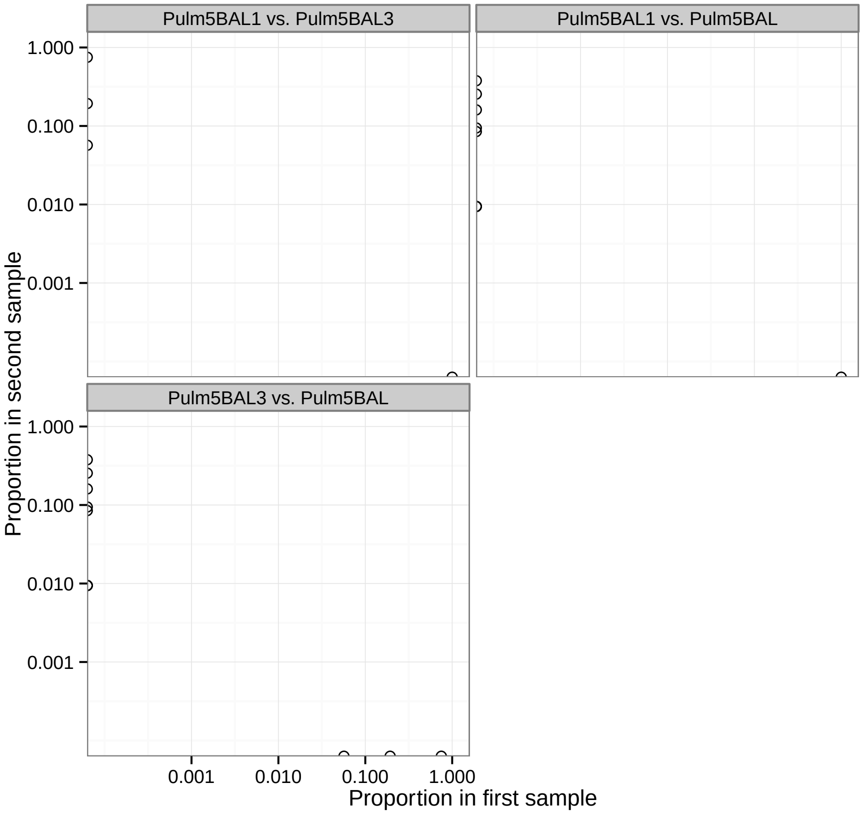

Figure 32: Proportion of fungal OTUs in repeat extractions of sample Pulm5 BAL (log scale). OTUs absent in one sample are shown along the axis.

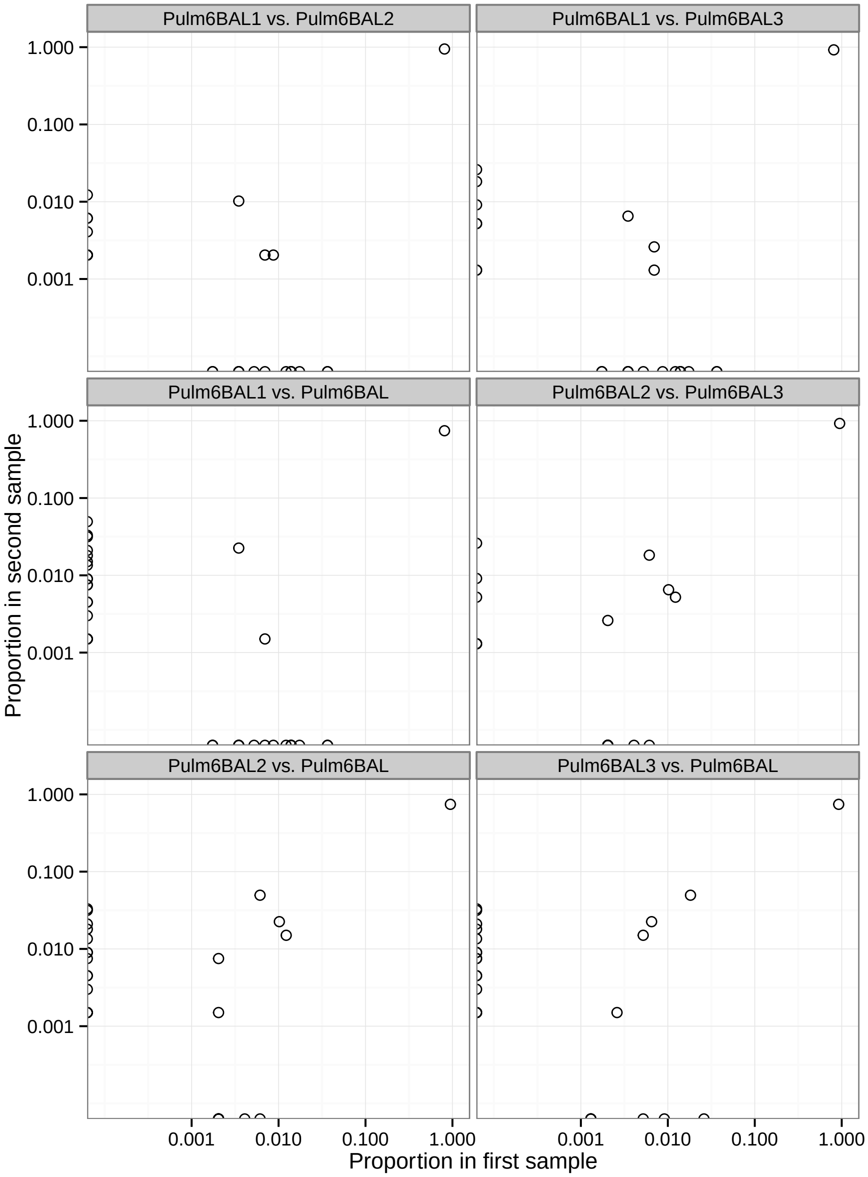

Figure 33: Proportion of fungal OTUs in repeat extractions of sample Pulm6 BAL (log scale). OTUs absent in one sample are shown along the axis.

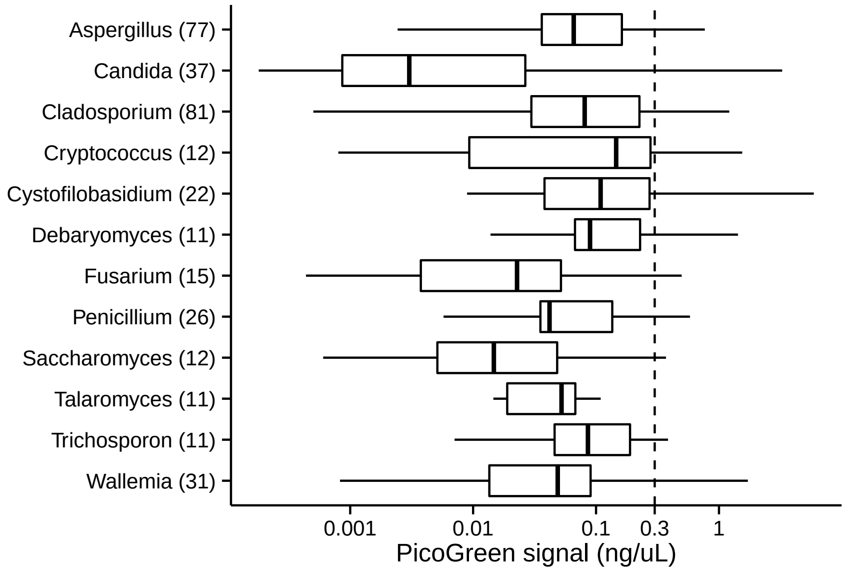

Figure 34: Non-zero OTU abundacnes for genera observed in 1 or more contamination control samples (log scale).

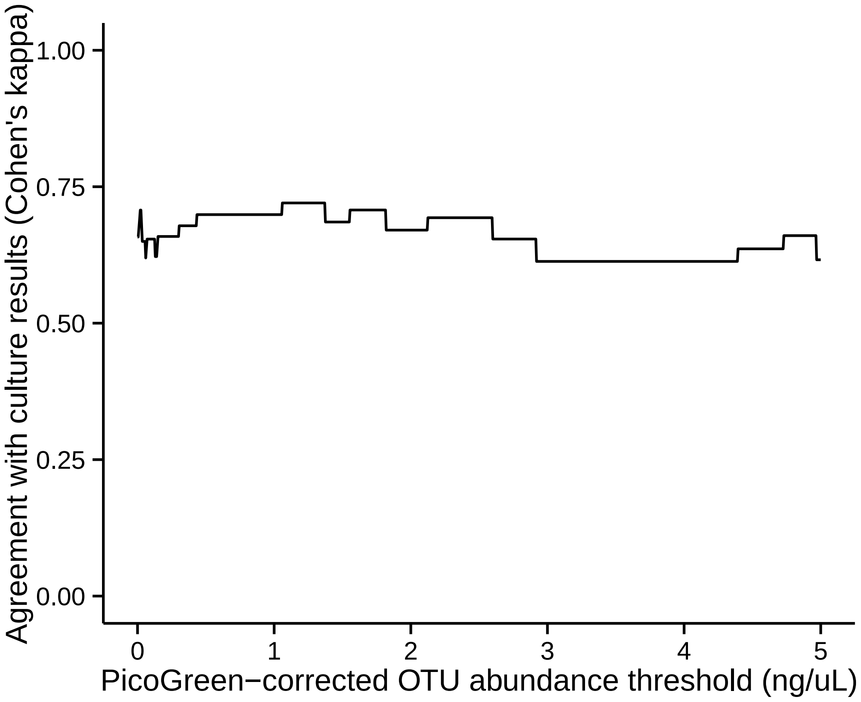

Figure 35: Agreement between culture results and PicoGreen-corrected ITS sequencing results is nearly constant over a wide range of thresholds.

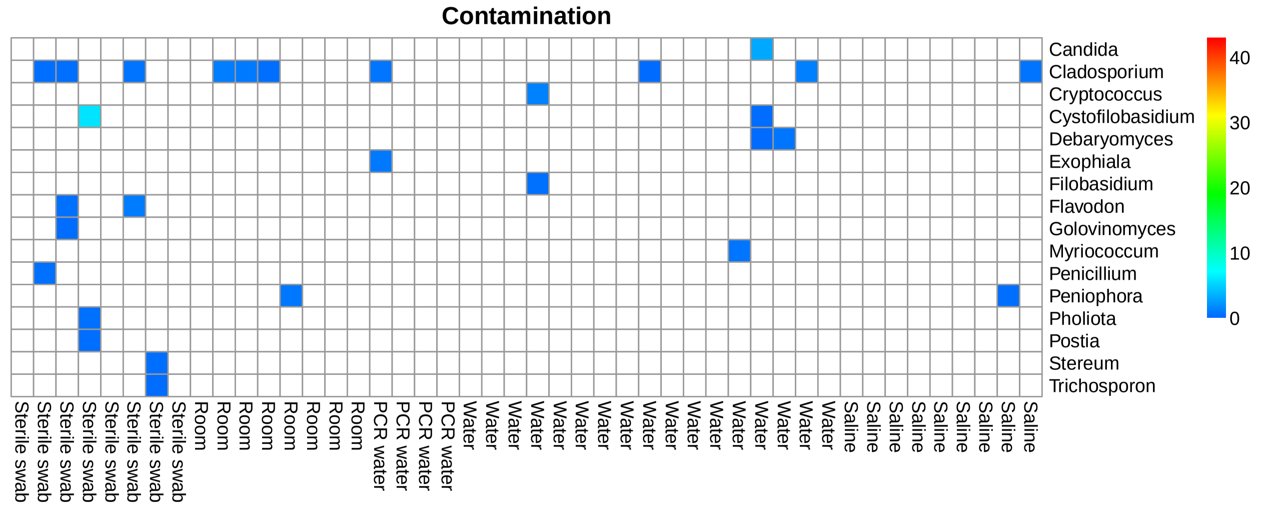

Figure 36: PicoGreen-corrected abundance of fungal genera exceeding the threshold value in Contamination samples.

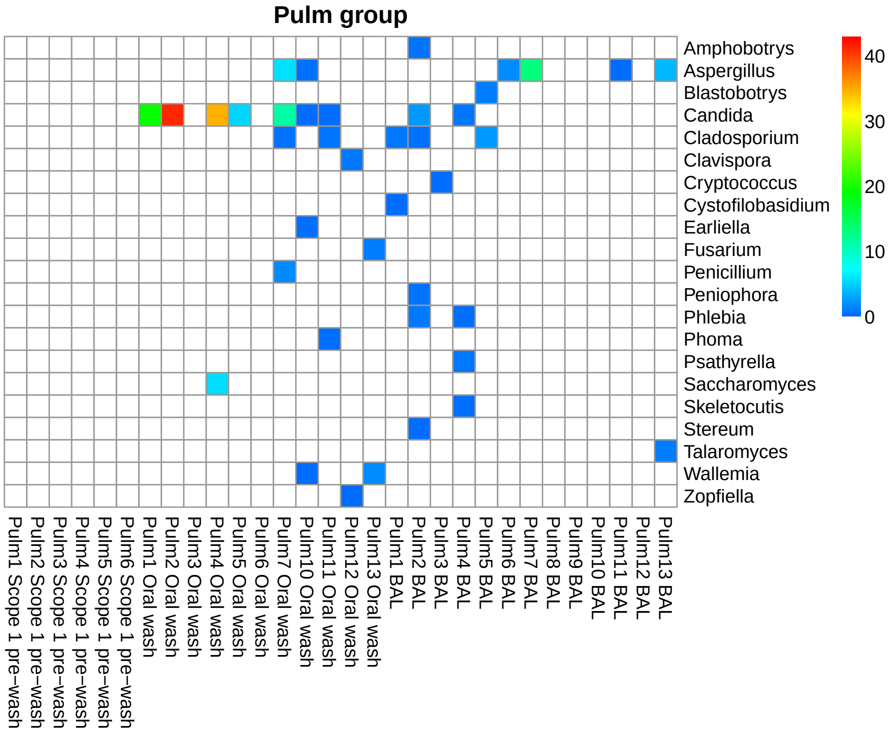

Figure 37: PicoGreen-corrected abundance of fungal genera exceeding the threshold value in Pulm group samples.

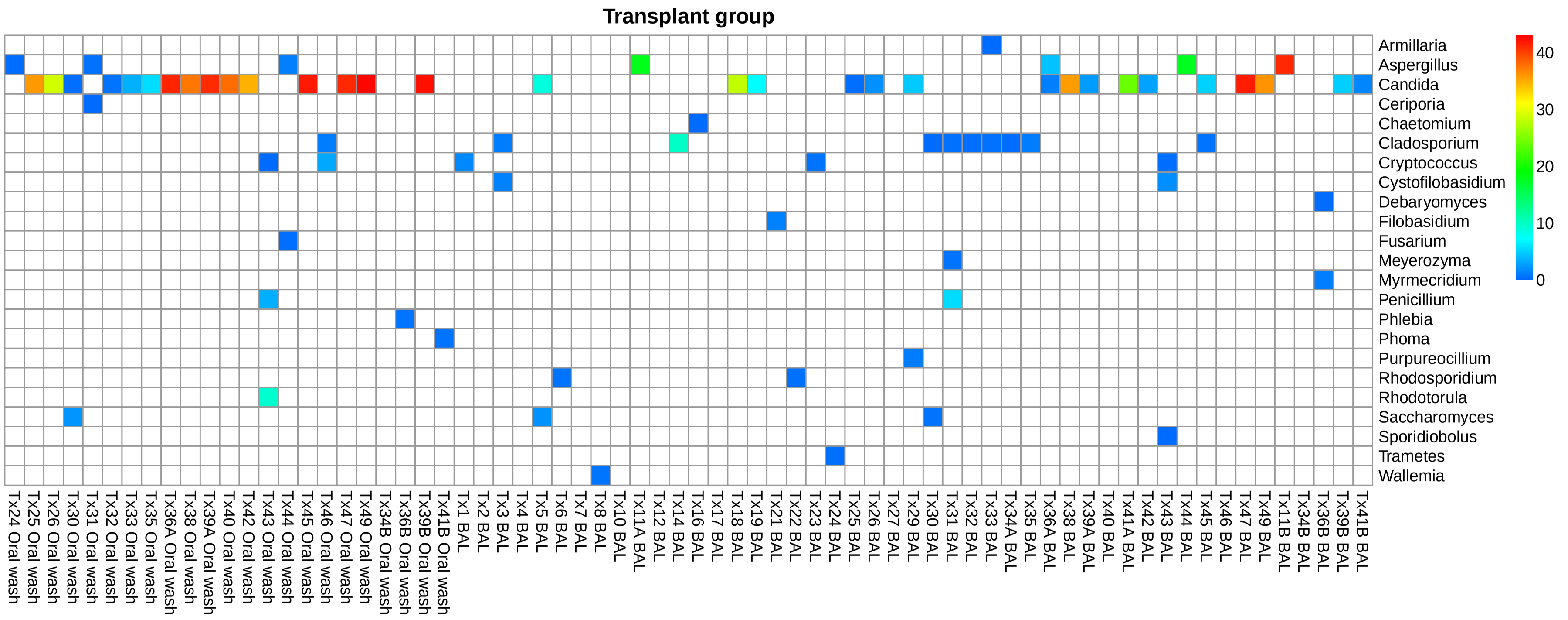

Figure 38: PicoGreen-corrected abundance of fungal genera exceeding the threshold value in Transplant group samples.

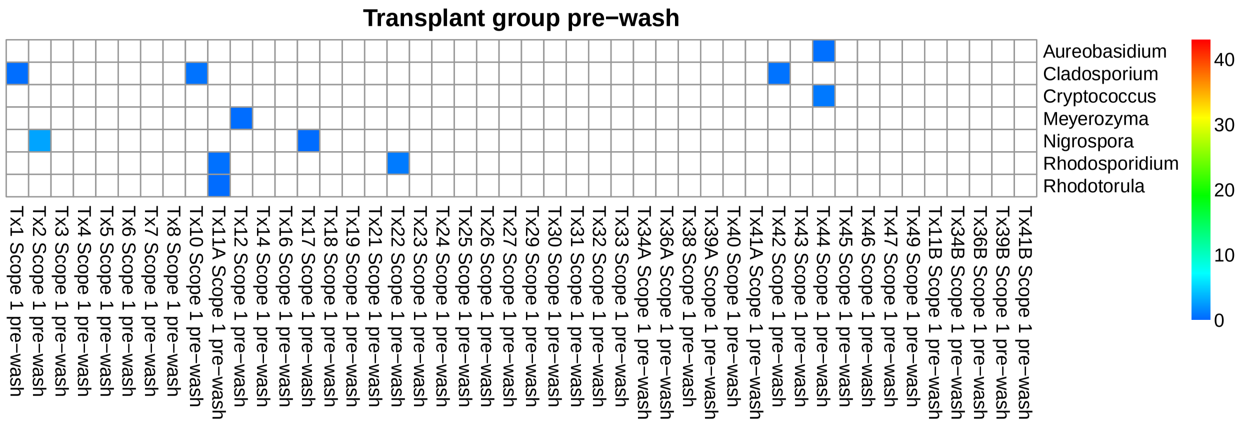

Figure 39: PicoGreen-corrected abundance of fungal genera exceeding the threshold value in Transplant group pre-wash samples.

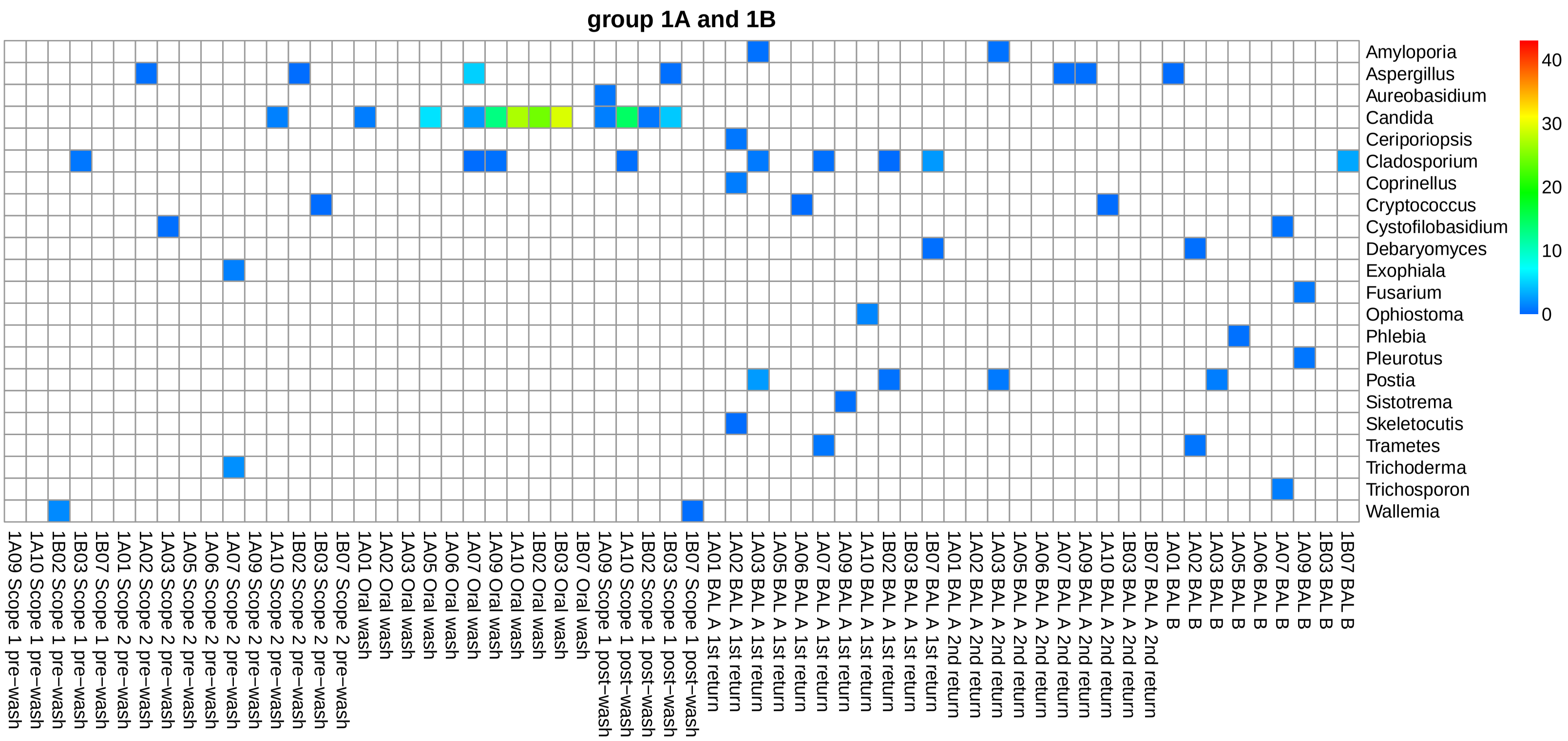

Figure 40: PicoGreen-corrected abundance of fungal genera exceeding the threshold value in group 1A and 1B samples.

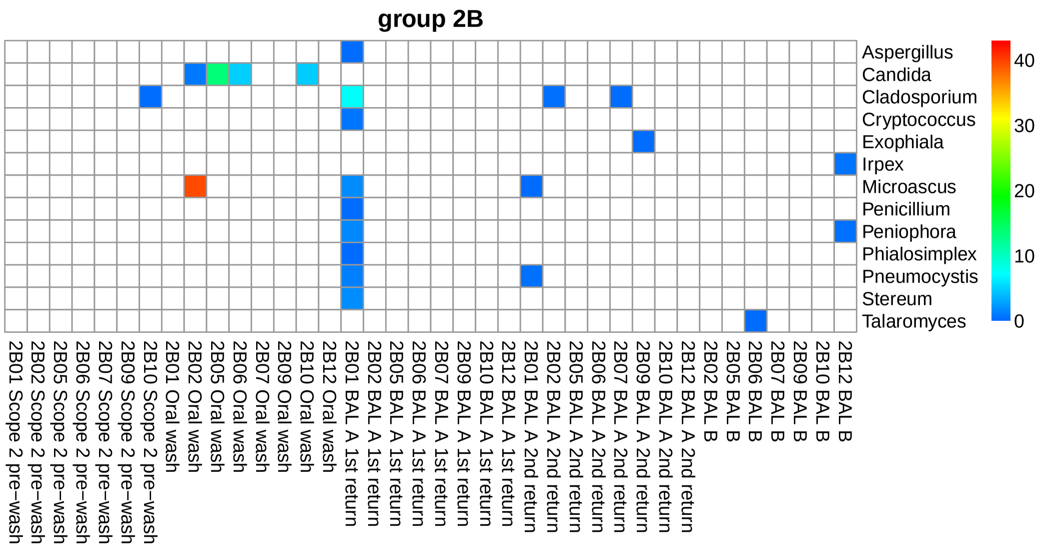

Figure 41: PicoGreen-corrected abundance of fungal genera exceeding the threshold value in group 2B samples.

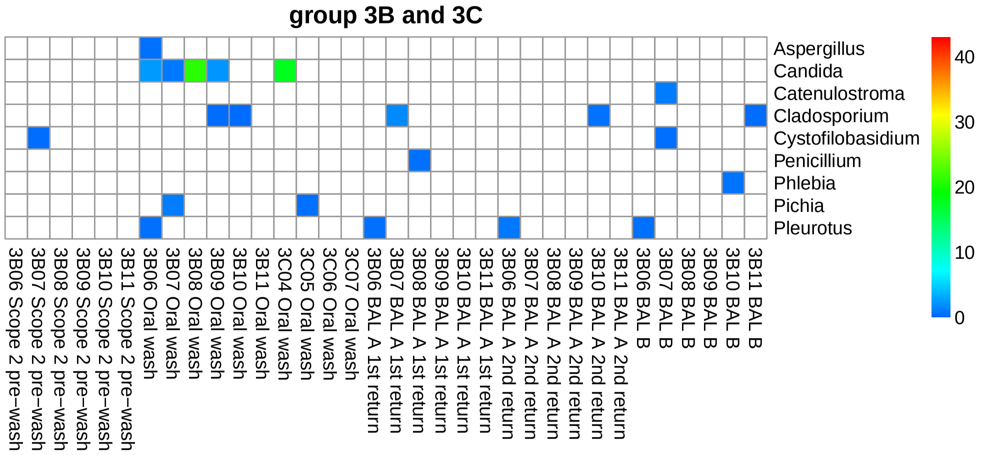

Figure 42: PicoGreen-corrected abundance of fungal genera exceeding the threshold value in group 3B and 3C samples.

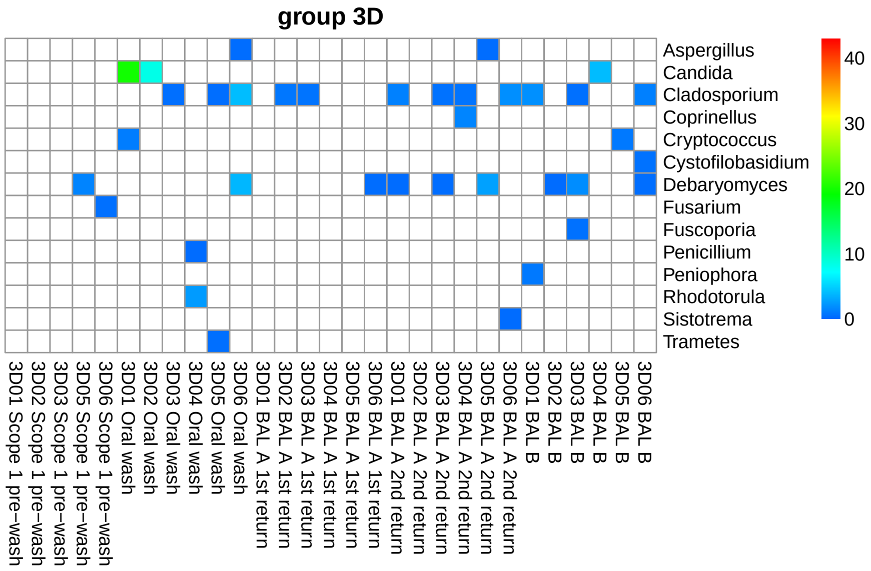

Figure 43: PicoGreen-corrected abundance of fungal genera exceeding the threshold value in group 3D samples.
