# Supplementary material for: Improved characterization of medically relevant fungi in the human respiratory tract using next-generation sequencing
Source: Genome Biol. 2014 Oct 25;15(10):487. doi: 10.1186/s13059-014-0487-y (PMC4232682; doi:10.1186/s13059-014-0487-y)
Supplement: Additional file 3: — Analysis of bacterial communities, covariation with Candida . [file 13059_2014_487_MOESM3_ESM.pdf]

Supplemental report: Anaysis of bacterial communities, covariation with *Candida*

Contents

1 Analysis of bacterial communities **2**
1.1 16S contamination control samples . . . 2
1.2 Oropharyngeal wash samples, all groups . . . 5
1.3 Oropharyngeal wash samples, HIV+ vs. healthy . . . 5
1.4 BAL samples, all groups . . . 8
1.5 BAL samples, HIV+ vs. healthy . . . 11

2 *Candida* co-variation with bacteria **11**
2.1 Effect of *Candida* on bacterial community composition . . . 11
2.2 Correlation with bacterial genera . . . 13
2.3 Correlation with Streptococcus OTUs . . . 13

1 Analysis of bacterial communities

1.1 16S contamination control samples

Table 1 lists the total number of samples for OW, BAL, and contamination control. Table 2 lists the number of each contamination control sample type. Bronchoscope pre-wash samples were obtained by drawing 10 mL of saline into the channel of a bronchoscope prior to bronchoscopy Water samples consisted of laboratory water used at the location where DNA extraction was performed. Saline samples were taken from the sources used for the bronchoalveolar lavage procedures. PSB surface samples were derived from protected specimen brushes used in the bronchoscopy procedure. Sterile swab samples were obtained from clean swabs used to sample the oropharyngeal and nasopharyngeal cavities (oropharyngeal and nasopharyngeal samples not included in this data set).

General sample type	N
Oral wash	95
BAL	98
Contamination control	151

Table 1: Number of OW, BAL, and contamination control samples included.

Contamination control source	N
Bronchoscope pre-wash	87
Water	32
Saline	22
PSB surface	2
Sterile swab	8

Table 2: Number of contamination control samples included by source.

Figure 1 shows weighted UniFrac distances between BAL, OW, and contamination control samples. Table 3 lists the results of a PERMANOVA test for difference in bacterial community composition between experimental (BAL and OW) vs. contamination control samples. A similar analysis was carred out for unweighted UniFrac distances, with results presented in Figure 2 and Table 4. Both distance measures yielded a highly significant difference in community composition between experimental and contamination control samples, with large effect sizes observed ($R^2 = 0.32$ and 0.19, respectively).

	df	SS	MS	F	R-squared	P
Experimental v. contamination	1	15.47	15.47	161.09	0.32	0.001
Residuals	342	32.84	0.10		0.68	
Total	343	48.31			1.00	

Table 3: PERMANOVA test for difference in group centroid between experimental (OW, BAL) and contamination control samples, weighted UniFrac distance.

	df	SS	MS	F	R-squared	P
Experimental v. contamination	1	19.85	19.85	78.11	0.19	0.001
Residuals	342	86.90	0.25		0.81	
Total	343	106.75			1.00	

Table 4: PERMANOVA test for difference in group centroid between experimental (OW, BAL) and contamination control samples, unweighted UniFrac distance.

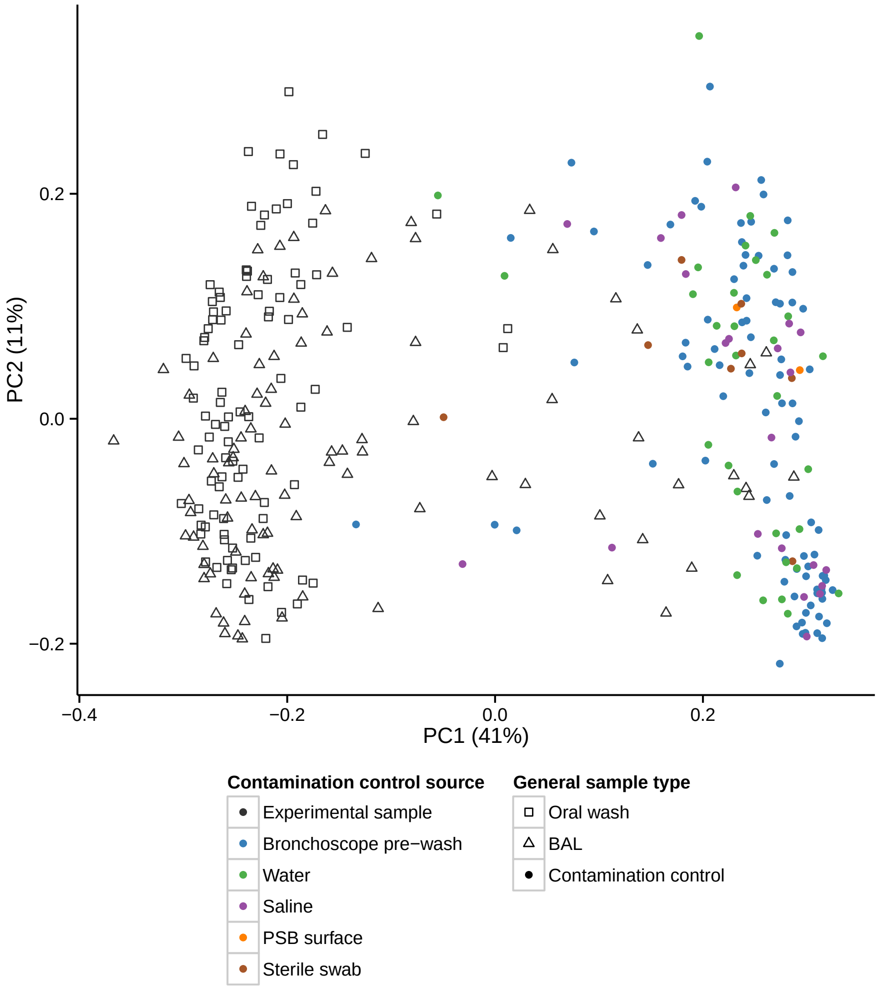

Figure 1: Principal Coordinates Analysis of weighted UniFrac distances between experimental and contamination control samples.

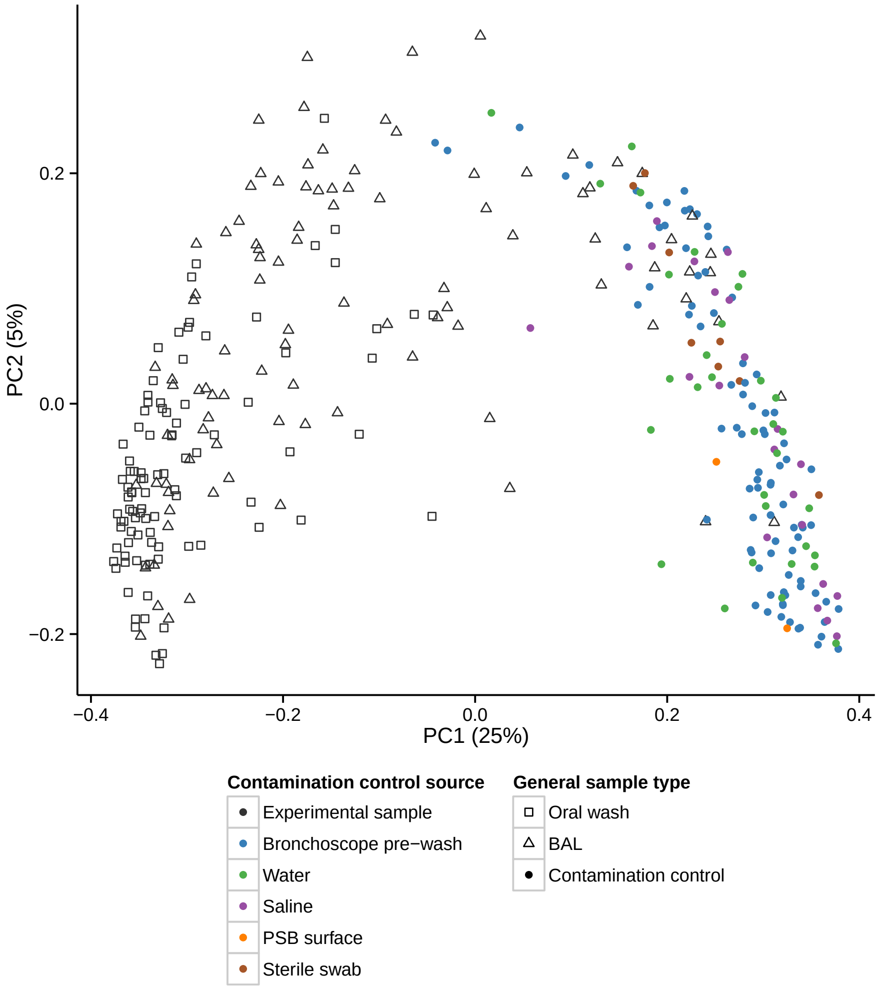

Figure 2: Principal Coordinates Analysis of unweighted UniFrac distances between experimental and contamination control samples.

1.2 Oropharyngeal wash samples, all groups

We examined 61 oropharyngeal wash (OW) samples to detect differences between Healthy (groups 3B, 3C, and 3D), HIV+ (groups 1A, 1B, and 2B), Pulm, and Transplant subjects. If subjects were sampled more than once, only the first time point was used (34 OW samples removed due to repeat sampling). Table 5 shows the number of OW samples included for each disease state.

disease_status	N
HIV+	19
Healthy	12
Pulm	11
Transplant	19

Table 5: Number of OW samples included for each disease state

We tested for differences in community composition between disease states using a PERMANOVA test, which takes a distance matrix as input and detects differences in group centroids. Figure 3 shows the weighted UniFrac distances between samples after ordination by principal coordinates (PCoA). The PERMANOVA test results are given in Table 6. Figure 4 shows the unweighted UniFrac distances between samples after PCoA ordination, and Table 7 gives the PERMANOVA test results. We found significant differences between disease states using both distance metrics.

	df	SS	MS	F	R-squared	P
disease status	3	0.44	0.15	2.32	0.11	0.005
Residuals	57	3.60	0.06		0.89	
Total	60	4.04			1.00	

Table 6: PERMANOVA test for difference in group centroid between Healthy, HIV+, Pulm, and Transplant oropharyngeal wash samples, weighted UniFrac distance.

	df	SS	MS	F	R-squared	P
disease status	3	1.51	0.50	2.12	0.10	0.001
Residuals	57	13.60	0.24		0.90	
Total	60	15.11			1.00	

Table 7: PERMANOVA test for difference in group centroid between Healthy, HIV+, Pulm, and Transplant oropharyngeal wash samples, unweighted UniFrac distance.

1.3 Oropharyngeal wash samples, HIV+ vs. healthy

Having found overall differences in bacterial community composition between disease states, we next tested for differences between healthy and HIV+ OW samples. Figure 5 shows a PCoA ordination of weighted UniFrac distances. A PERMANOVA test, detects no significant difference in group centroids using a weighted UniFrac distance (results in Table 8). Figure 6 shows the unweighted UniFrac distances between samples after PCoA ordination, and Table 9 gives the associated PERMANOVA test results. Using the unweighted UniFrac metric, we found significant differences between healthy and HIV+ OW communities.

We examined the OTU abundances in OW samples to see if any OTUs distinguished the healthy subjects and from HIV+ subjects. We used a Kruskal-Wallis test to test for a difference in median proportion of each OTU appearing in at least 5 samples, then adjusted the p-values for multiple comparison using the method of Benjamini and Hochberg. Table 10 lists the 20 OTUs having the most significantly different proportions in OW samples of healthy vs. HIV+ subjects. All of the abundant oral genera appear in the table, including *Streptococcus*, *Veionella*, *Prevotella*,

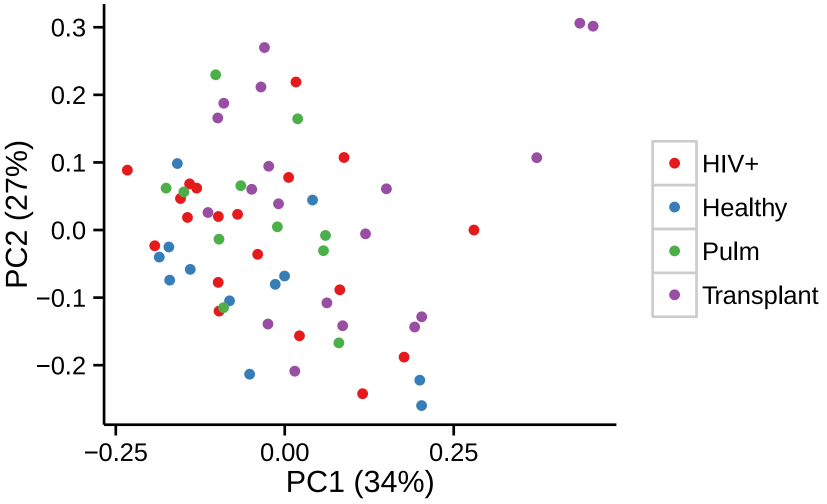

Figure 3: Principal Coordinates Analysis of weighted UniFrac distances between oropharyngeal wash samples.

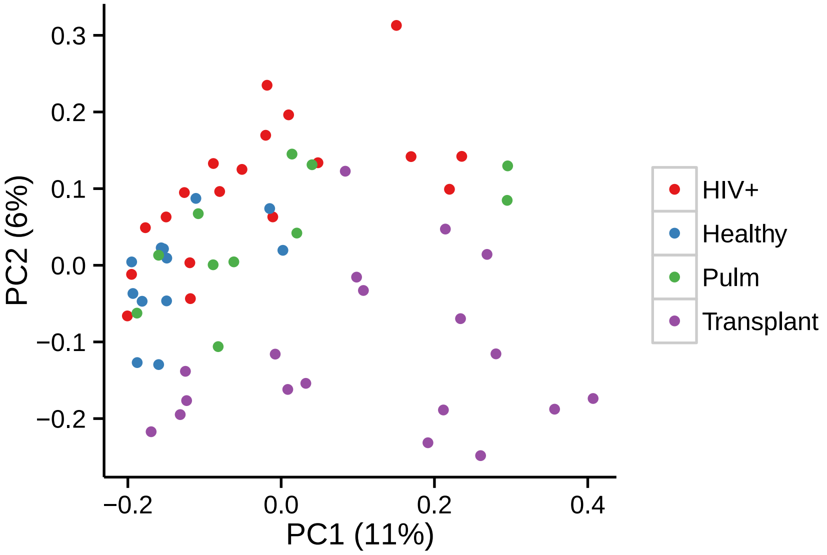

Figure 4: Principal Coordinates Analysis of unweighted UniFrac distances between oropharyngeal wash samples.

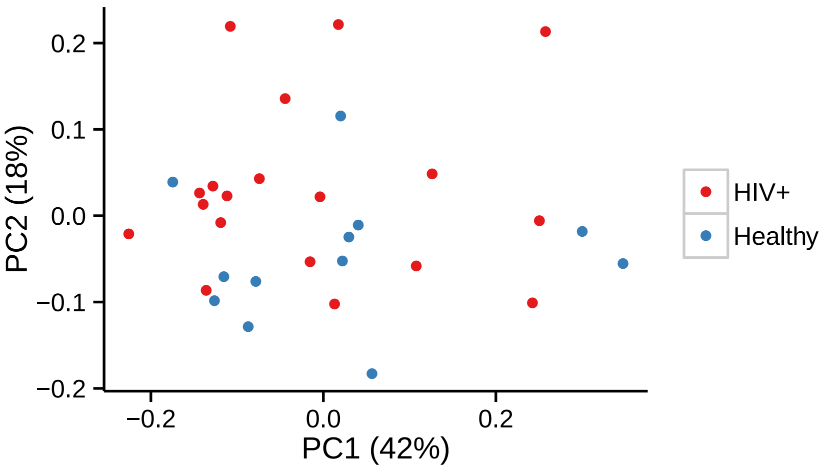

Figure 5: Principal Coordinates Analysis of weighted UniFrac distances between oropharyngeal wash samples, HIV+ vs. healthy.

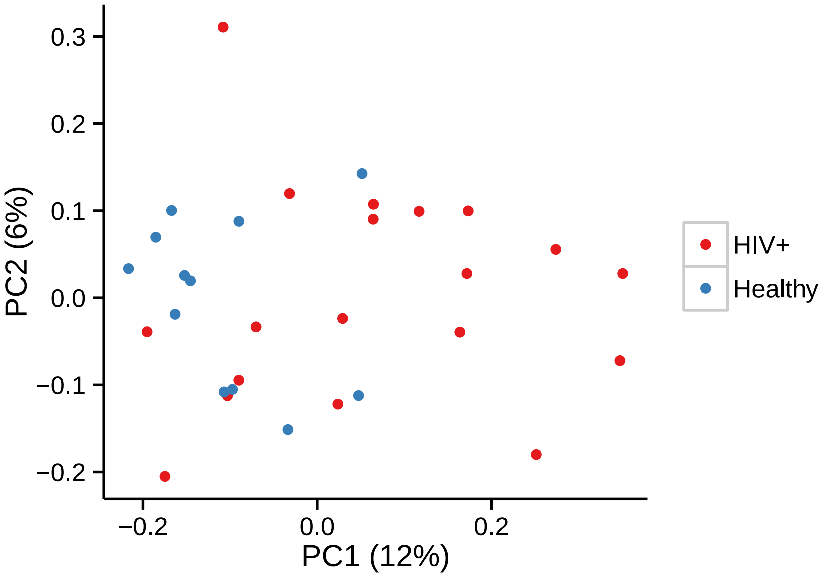

Figure 6: Principal Coordinates Analysis of unweighted UniFrac distances between oropharyngeal wash samples, HIV+ vs. healthy.

	df	SS	MS	F	R-squared	P
disease status	1	0.06	0.06	1.22	0.04	0.280
Residuals	29	1.54	0.05		0.96	
Total	30	1.60			1.00	

Table 8: PERMANOVA test for difference in group centroid between Healthy and HIV+ oropharyngeal wash samples, weighted UniFrac distance.

	df	SS	MS	F	R-squared	P
disease status	1	0.37	0.37	1.74	0.06	0.002
Residuals	29	6.16	0.21		0.94	
Total	30	6.53			1.00	

Table 9: PERMANOVA test for difference in group centroid between Healthy and HIV+ oropharyngeal wash samples, unweighted UniFrac distance.

Haemophilus, and *Neissera*. Notably, the proportion of all but 1 OTU is higher in healthy samples. Seven OTUs appearing in the table are completely absent in HIV+ samples.

	Assignment	H	P-value	FDR	HIV+ mean	Healthy mean
denovo21853	Bacteroidetes [Prevotella]	12.3	0.0005	0.02	8.524e-05	3.681e-04
denovo5434	Unassigned	11.6	0.0007	0.02	2.588e-05	2.582e-04
denovo4370	Proteobacteria Campylobacter	11.5	0.0007	0.02	2.435e-03	6.047e-03
denovo3305	Proteobacteria Neisseria	11.3	0.0008	0.02	0.000e+00	7.055e-05
denovo20194	Bacteroidetes Prevotella	11.2	0.0008	0.02	7.529e-05	5.392e-04
denovo15434	Firmicutes Streptococcus	10.9	0.0010	0.02	2.343e-03	1.320e-04
denovo17293	Bacteroidetes Prevotella	10.9	0.0010	0.02	1.222e-05	1.403e-04
denovo15744	Firmicutes Streptococcus	10.8	0.0010	0.02	5.574e-06	1.347e-04
denovo13709	Proteobacteria Haemophilus	9.7	0.0018	0.02	6.403e-04	2.499e-03
denovo16051	Bacteroidetes Capnocytophaga	9.3	0.0023	0.02	2.349e-04	1.054e-03
denovo7255	Proteobacteria Haemophilus	9.2	0.0024	0.02	4.508e-05	3.106e-04
denovo8815	Bacteroidetes [Prevotella]	9.1	0.0025	0.02	0.000e+00	5.387e-05
denovo16140	Firmicutes Streptococcus	9.1	0.0026	0.02	0.000e+00	6.396e-05
denovo1921	Firmicutes Veillonella	9.1	0.0026	0.02	0.000e+00	8.208e-05
denovo6514	Proteobacteria Haemophilus	9.1	0.0026	0.02	0.000e+00	9.618e-05
denovo3427	Bacteroidetes Prevotella	9.1	0.0026	0.02	0.000e+00	1.651e-04
denovo10821	Firmicutes Veillonella	9.1	0.0026	0.02	0.000e+00	8.722e-05
denovo18038	Firmicutes Veillonella	8.8	0.0030	0.02	1.340e-05	8.688e-04
denovo17328	Unassigned	8.6	0.0035	0.02	1.314e-05	1.593e-04
denovo19570	Proteobacteria Haemophilus	8.5	0.0036	0.02	8.956e-06	1.028e-04

Table 10: OTUs having significantly different abundance in OW samples between healthy and HIV+ subjects. The columns list the taxonomic assignment (phylum and genus), the Kruskal-Wallis Chi-square statistic (H), the unadjusted p-value from the test, the FDR-adjusted value, the mean proportion in healthy subjects, and the mean proportion in HIV+ subjects.

Only one OTU, a *Streptococcus* species (denovo15434), appears with greater mean proportion in HIV+ samples. Figure 7 shows a histogram of the proportions detected in each group. The OTU is absent in 8 of 12 healthy samples, but present in all 17 of 19 HIV+ samples.

1.4 BAL samples, all groups

We next examined 89 bronchoalveolar lavage fluid (BAL) samples to detect differences between Healthy (groups 3B, 3C, and 3D), HIV+ (groups 1A, 1B, and 2B), Pulm, and Transplant subjects.

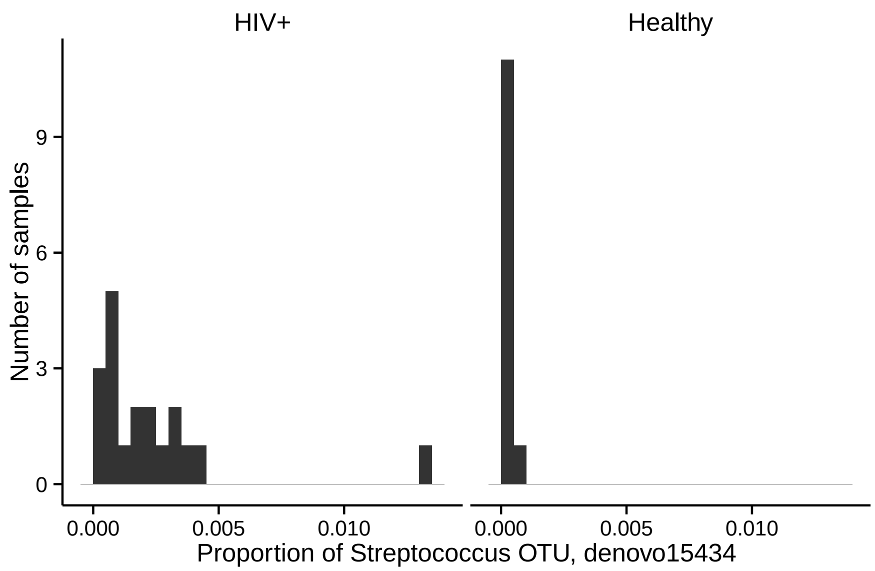

Figure 7: Histogram showing the proportions of OTU denovo15434 detected in OW samples from healthy and HIV+ subjects.

Again, only the first time point was used if subjects were sampled more than once. Where multiple BAL samples were available (groups 1A, 1B, 2B, 3B, 3C, and 3D), the BAL A 2nd return sample was used in the analysis. Table 11 shows the number of BAL samples included for each disease state.

disease_status	N
HIV+	18
Healthy	8
Pulm	19
Transplant	44

Table 11: Number of BAL samples included for each disease state

Figure 8 shows the a PCoA ordination of weighted UniFrac distances between BAL samples after ordination by principal coordinates (PCoA). The PERMANOVA test results are given in Table 12. Figure 9 shows the unweighted UniFrac distances between samples after PCoA ordination, and Table 13 gives the PERMANOVA test results. We found significant differences between disease states using both distance metrics.

	df	SS	MS	F	R-squared	P
disease status	3	0.89	0.30	2.46	0.08	0.002
Residuals	85	10.18	0.12		0.92	
Total	88	11.07			1.00	

Table 12: PERMANOVA test for difference in group centroid between Healthy, HIV+, Pulm, and Transplant BAL samples, weighted UniFrac distance.

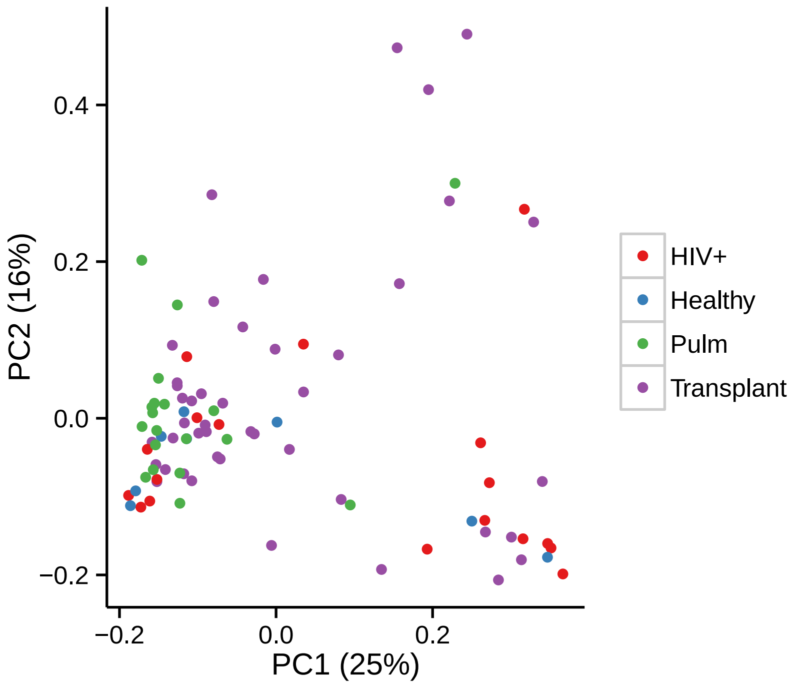

Figure 8: Principal Coordinates Analysis of weighted UniFrac distances between BAL samples.

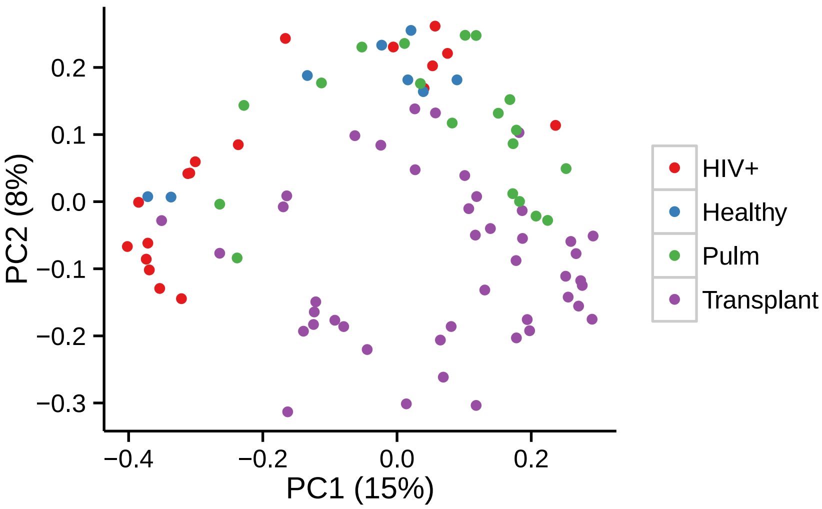

Figure 9: Principal Coordinates Analysis of unweighted UniFrac distances between BAL samples.

	df	SS	MS	F	R-squared	P
disease status	3	2.42	0.81	3.10	0.10	0.001
Residuals	85	22.12	0.26		0.90	
Total	88	24.54			1.00	

Table 13: PERMANOVA test for difference in group centroid between Healthy, HIV+, Pulm, and Transplant BAL samples, unweighted UniFrac distance.

1.5 BAL samples, HIV+ vs. healthy

We next tested for differences between healthy and HIV+ BAL samples. Figure 10 shows a PCoA ordination of weighted UniFrac distances; PERMANOVA test results are listed in Table 14. Figure 11 shows a PCoA ordination of unweighted UniFrac distances between samples, and Table 15 gives the associated PERMANOVA test results. We found no significant differences between healthy and HIV+ bacterial communities in BAL samples.

	df	SS	MS	F	R-squared	P
disease status	1	0.11	0.11	1.08	0.04	0.318
Residuals	24	2.48	0.10		0.96	
Total	25	2.59			1.00	

Table 14: PERMANOVA test for difference in group centroid between Healthy and HIV+ BAL samples, weighted UniFrac distance.

	df	SS	MS	F	R-squared	P
disease status	1	0.27	0.27	1.14	0.05	0.226
Residuals	24	5.64	0.24		0.95	
Total	25	5.91			1.00	

Table 15: PERMANOVA test for difference in group centroid between Healthy and HIV+ BAL samples, unweighted UniFrac distance.

2 *Candida* co-variation with bacteria

2.1 Effect of *Candida* on bacterial community composition

We examined the association between PicoGreen-corrected *Candida* abundance and bacterial community composition in OW samples by two methods. First, we divided the samples into low and high *Candida* abundance using the 75% quertile as a cutoff. Figure 12 shows a PCoA plot of OW samples using the weighted UniFrac metric. Low vs. high *Candida* abundance had a sgnificant effect on community composition (Table 16). An analysis of unweighted UniFrac distances is presented in Figure 13 and Table 17. We also found a significant effect in unweighted UniFrac.

	df	SS	MS	F	R-squared	P
CandidaHigh	1	0.28	0.28	4.26	0.07	0.003
Residuals	57	3.75	0.07		0.93	
Total	58	4.04			1.00	

Table 16: PERMANOVA test for difference in group centroids, high vs. low Candida abundance in OW samples, weighted UniFrac metric.

As a second approach, we used the PicoGreen-corrected abundance of *Candida* to test for association with UniFrac distance, without dividing the samples into low and high *Candida* abundance

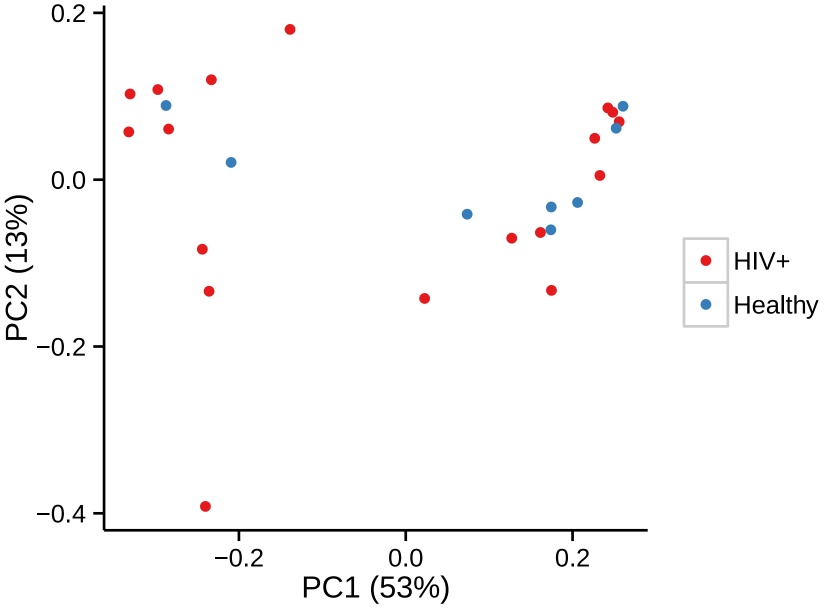

Figure 10: Principal Coordinates Analysis of weighted UniFrac distances between BAL samples, HIV+ vs. healthy.

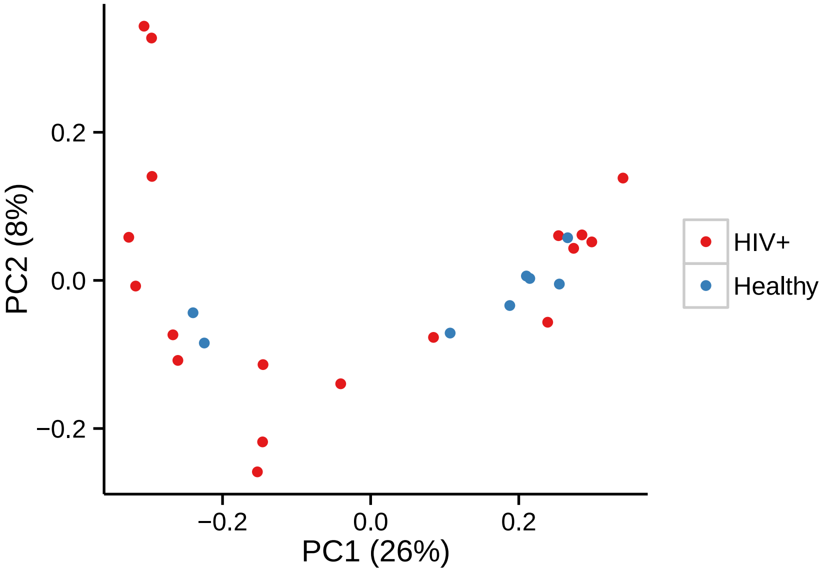

Figure 11: Principal Coordinates Analysis of unweighted UniFrac distances between BAL samples, HIV+ vs. healthy.

	df	SS	MS	F	R-squared	P
CandidaHigh	1	0.59	0.59	2.31	0.04	4e-04
Residuals	57	14.53	0.25		0.96	
Total	58	15.12			1.00	

Table 17: PERMANOVA test for difference in group centroids, high vs. low Candida abundance in OW samples, unweighted UniFrac metric.

groups. The test used is analagous to Anderson's PERMANOVA test, and is implemented in the `adonis` function provided by the *vegan* package for R. We call this test ADONIS to distinguish it from Anderson's original test, which was formulated for discrete groups only.

Figure 14 shows a PCoA plot of weighted UniFrac distances, where samples are colored by PicoGreen-corrected abundance of *Candida*. The test, summarized in Table 18, indicates a significant association. The unweighted UniFrac analysis is presented in Figure 15 and Table 19, and also reports a significant effect.

	df	SS	MS	F	R-squared	P
CandidaPG	1	0.32	0.32	4.87	0.08	0.002
Residuals	57	3.72	0.07		0.92	
Total	58	4.04			1.00	

Table 18: ADONIS test for dependence of weighted UniFrac distance on PicoGreen-corrected Candida abundance in OW samples.

	df	SS	MS	F	R-squared	P
CandidaPG	1	0.63	0.63	2.46	0.04	4e-04
Residuals	57	14.49	0.25		0.96	
Total	58	15.12			1.00	

Table 19: ADONIS test for dependence of unweighted UniFrac distance on PicoGreen-corrected Candida abundance in OW samples.

2.2 Correlation with bacterial genera

Having found significant associations in OW samples at the community level, we next investigated the top 10 bacterial genera to look for association of qPCR-corrected genus abundance with *Candida* abundance. We used a one-sided test of Spearman correlation to assess positive covariation of each genus with *Candida*. The test results are listed in Table 20. We found that the abundance of *Streptococcus* was highly correlated with *Candida* abundance. A plot of ranked bacterial genus abundance vs. *Candida* abundance is shown in Figure 16. Two other genera, *Rothia* and *Veillonella*, were found to be associated with *Candida* after adjusting for multiple comparisons.

2.3 Correlation with Streptococcus OTUs

We next investigated *Streptococcus* at the OTU level to see if the overall correlation with *Candida* abundance could be traced to individual species. We tested the top 20 OTUs, ranking by median abundance in all OW samples. The OTU rank-transformed OTU abundances are plotted against ranked *Candida* abundance in Figure 17.

Table 21 lists the results of a one-sided test of Spearman correlation for each OTU. We found that the proportions of 10 of the 20 OTUs tested were positively correlated with *Candida*, including the three most abundant OTUs. Of the OTUs positively correlated, all but two were in the *S. mitis* group. Both exceptions were assigned to the species *S. salivarius*.

	Median abundance	Spearman correlation	P-value	FDR
Rothia	1.39E+06	0.42	0.0005	0.003
Streptococcus	3.76E+06	0.41	0.0006	0.003
Veillonella	1.76E+06	0.29	0.0130	0.043
Granulicatella	4.16E+05	0.24	0.0322	0.081
Prevotella	3.11E+06	0.18	0.0908	0.177
Actinomyces	2.43E+05	0.16	0.1065	0.177
Haemophilus	1.95E+05	-0.03	0.5770	0.764
Fusobacterium	3.73E+05	-0.05	0.6525	0.764
Neisseria	2.62E+05	-0.08	0.7143	0.764
[Prevotella]	2.12E+05	-0.10	0.7644	0.764

Table 20: Tests of positive correlation between qPCR-corrected bacterial genus abundance and PicoGreen-corrected Candida abundance in OW samples.

	Species	Median abundance	P-value	FDR
denovo13840	S. parasanguinus	4.44E+04	0.0004	0.005
denovo10659	S. salivarius	1.04E+05	0.0005	0.005
denovo1048	S. parasanguinus	2.21E+05	0.0012	0.008
denovo15434	S. gordonii	6.49E+03	0.0027	0.014
denovo22088	S. parasanguinus	1.37E+04	0.0043	0.015
denovo22729	S. salivarius	4.10E+03	0.0045	0.015
denovo2114	S. oralis	5.46E+02	0.0052	0.015
denovo18769	S. mitis	1.04E+06	0.0077	0.019
denovo4966	S. oralis	7.69E+04	0.0095	0.021
denovo10339	S. parasanguinus	2.44E+04	0.0136	0.027
denovo15201	S. australis	6.99E+04	0.1026	0.187
denovo6346	S. gordonii	1.61E+03	0.2204	0.367
denovo671	S. sanguinus	4.44E+04	0.2772	0.426
denovo9656	S. infantis	4.05E+04	0.4366	0.574
denovo12769	S. peroris	1.48E+03	0.4555	0.574
denovo13464	S. mitis	7.21E+03	0.4591	0.574
denovo17469	S. parasanguinus	1.38E+04	0.5174	0.609
denovo10931	S. mitis	5.30E+04	0.7686	0.854
denovo16703	S. cristatus	7.78E+03	0.8316	0.875
denovo18015	S. mitis	7.30E+02	0.9836	0.984

Table 21: Tests of positive correlation between Streptococcus OTU abundance and PicoGreen-corrected Candida abundance in OW samples.

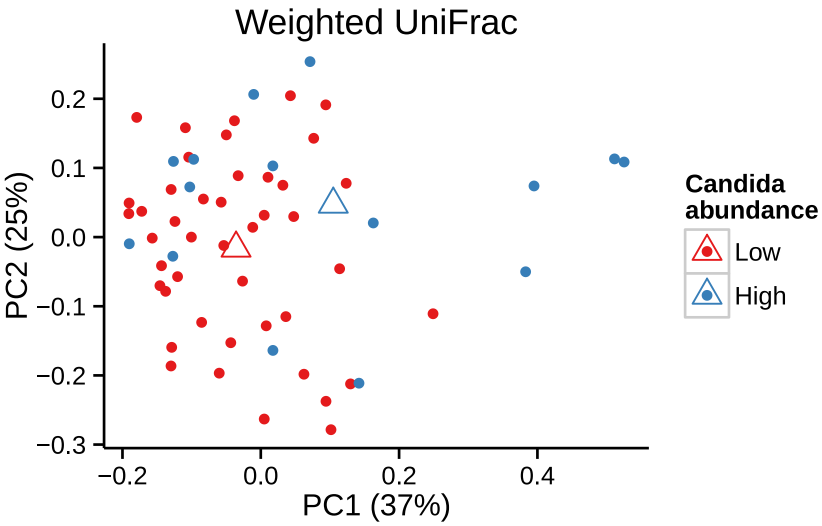

Figure 12: Weighted UniFrac distance between OW samples plotted after ordination with PCoA. PicoGreen-corrected *Candida* abundance is defined as high if it exceeds the 75% quantile for OW samples. The open triangles indicate group centroids.

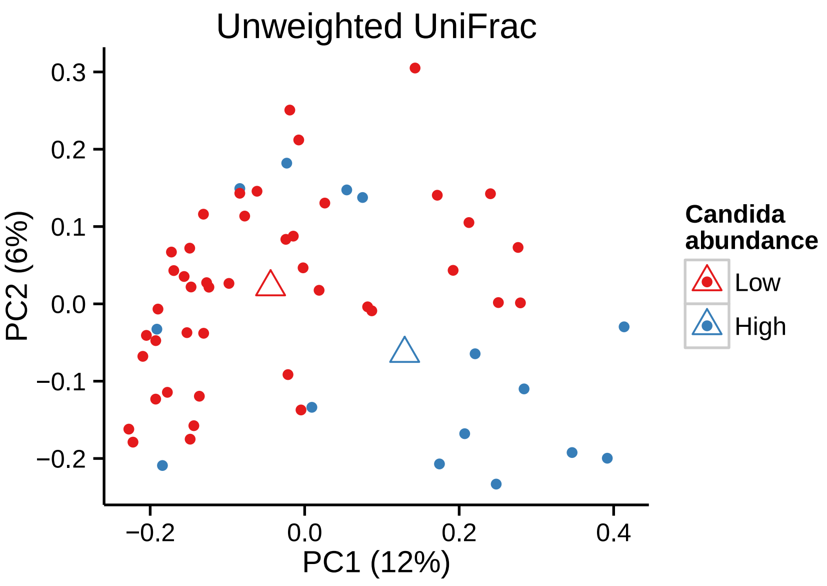

Figure 13: Unweighted UniFrac distance between OW samples plotted after ordination with PCoA. PicoGreen-corrected *Candida* abundance is defined as high if it exceeds the 75% quantile for OW samples. The open triangles indicate group centroids.

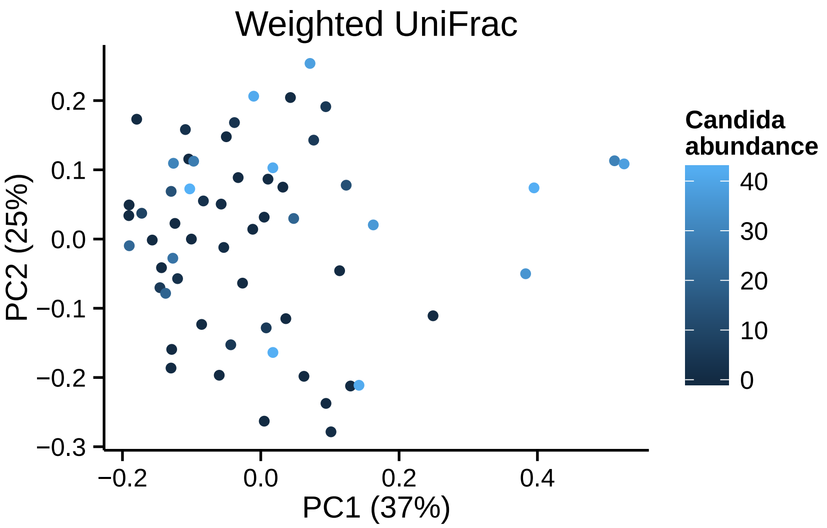

Figure 14: Weighted UniFrac distance between OW samples plotted after ordination with PCoA. PicoGreen-corrected *Candida* abundance is indicated by the color of each point.

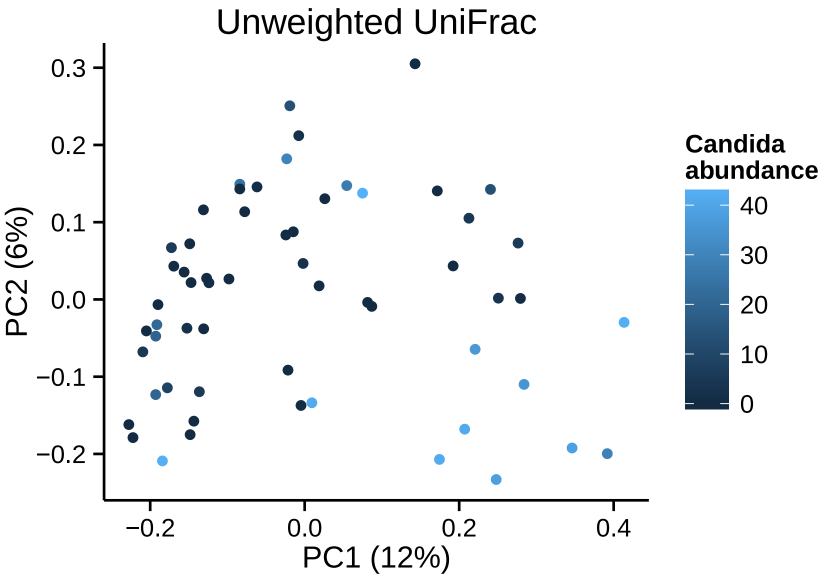

Figure 15: Unweighted UniFrac distance between OW samples plotted after ordination with PCoA. PicoGreen-corrected *Candida* abundance is indicated by the color of each point.

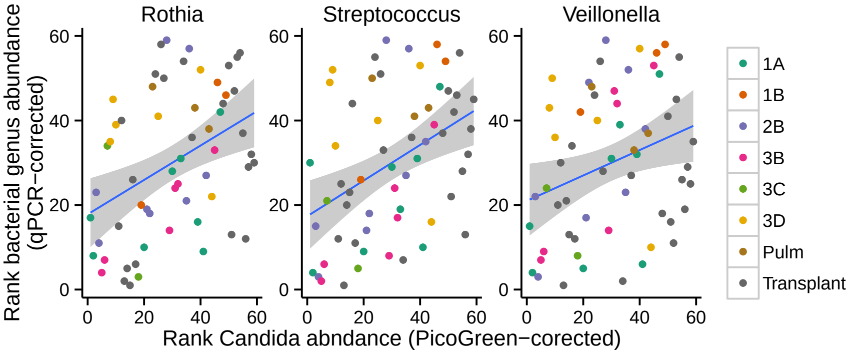

Figure 16: Abundance of bacterial genera Rothia, Streptococcus, and Veillonella plotted against PicoGreen-corrected *Candida* abundance in OW samples.

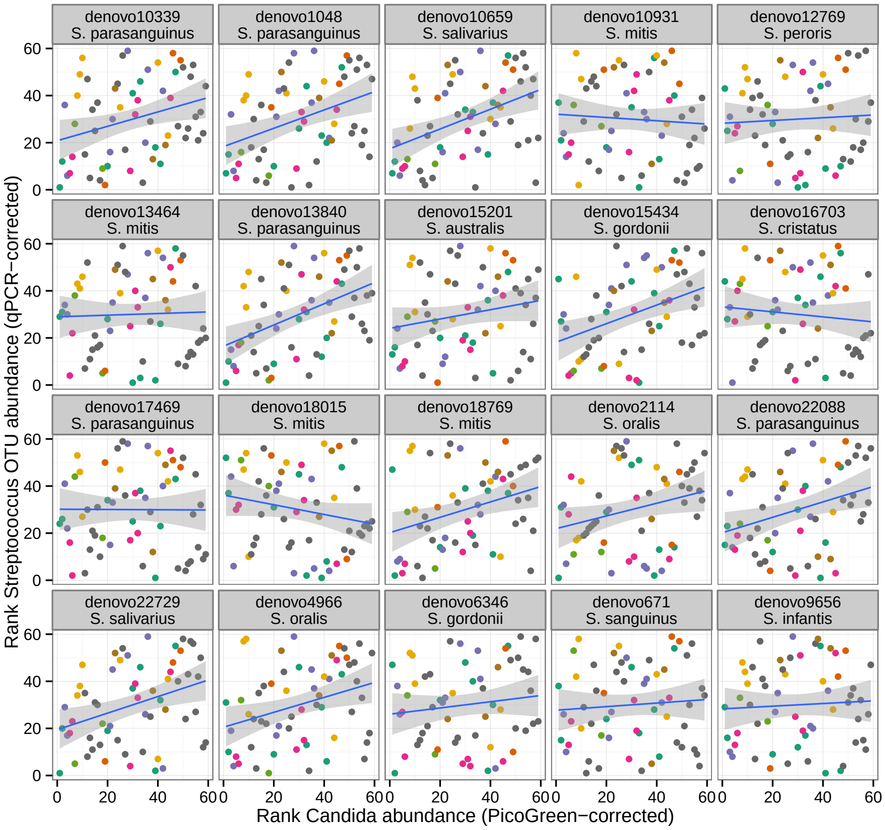

Figure 17: Abundance of Streptococcus OTUs plotted against PicoGreen-corrected *Candida* abundance in OW samples.
